# Supplementary material for: Archaeal histone-based chromatin structures regulate transcription elongation rates
Source: Commun Biol. 2024 Feb 27;7:236. doi: 10.1038/s42003-024-05928-w (PMC10899632; doi:10.1038/s42003-024-05928-w)
Supplement: Supplementary file 2 — Supplemental Materials [file 42003_2024_5928_MOESM2_ESM.pdf]

Supplementary Information for:

Archaeal histone-based chromatin structures regulate transcription elongation rates.

Breanna R. Wenck[1], Robert L. Vickerman[1], Brett W. Burkhart[1], and Thomas J. Santangelo[1]*

[1] Department of Biochemistry and Molecular Biology, Colorado State University, Fort Collins, Colorado, 80523, USA

* - Correspondence: thomas.santangelo@colostate.edu; phone +1-970-491-3150

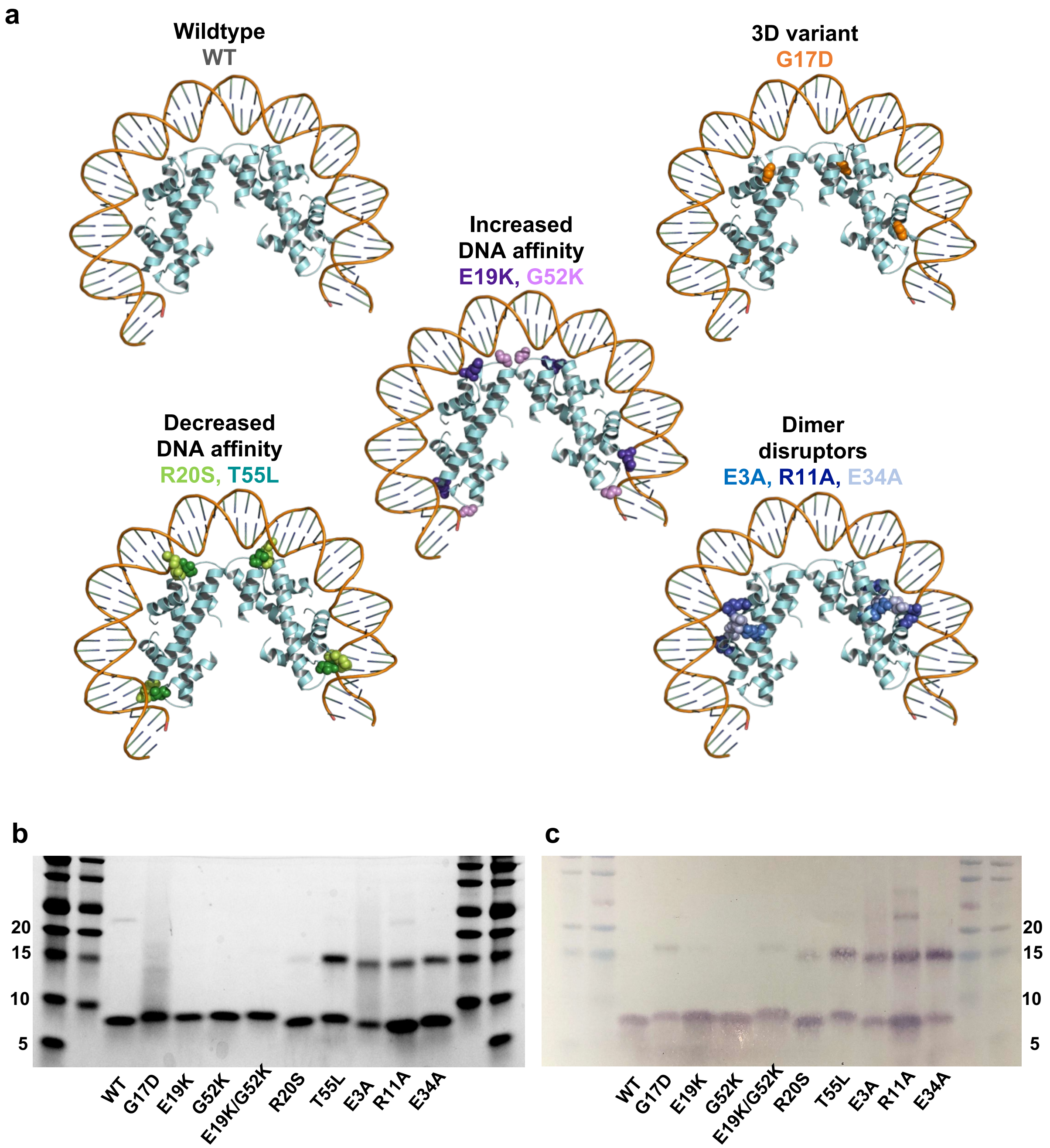

Supplementary Figure 1. Substitution of select histone residues provides a platform to evaluate the impacts of histone-based chromatin on transcription elongation rates and RNAP processivity.

(**a**) Cartoon representations of archaeal histone-based chromatin structures formed with HTkA^{WT} or HTkA^{variants} tetramers bound to ~60 bp of DNA. HTkA-variants were designed to

hinder formation of a tightly packed 3D structure of archaeal-histone based chromatin (HTkA^{G17D}), increase (HTkA^{E19K}, HTkA^{G52K}, and HTkA$^{E19K/G52K)}$ or decrease (HTkA^{R20S} and HTkA^{T55L}) histone-DNA affinity, or interfere with internal dimer, or dimer-dimer interactions supporting tetrasome formation (HTkA^{E3A}, HTkA^{R11A}, and HTkA^{E34A}). (**b**) Denaturing gels reveal the expected migration of recombinantly expressed and purified HTkA proteins primarily as monomers although some variants resolved as dimers or higher-order complexes during SDS-PAGE visualized with Coomassie brilliant blue R-250. (**c**) A Western blot employing polyclonal anti-HTkA antibodies confirms that higher order structures revealed by SDS-PAGE are multimeric histone complexes. Protein molecular weight markers provide standards in kDa.

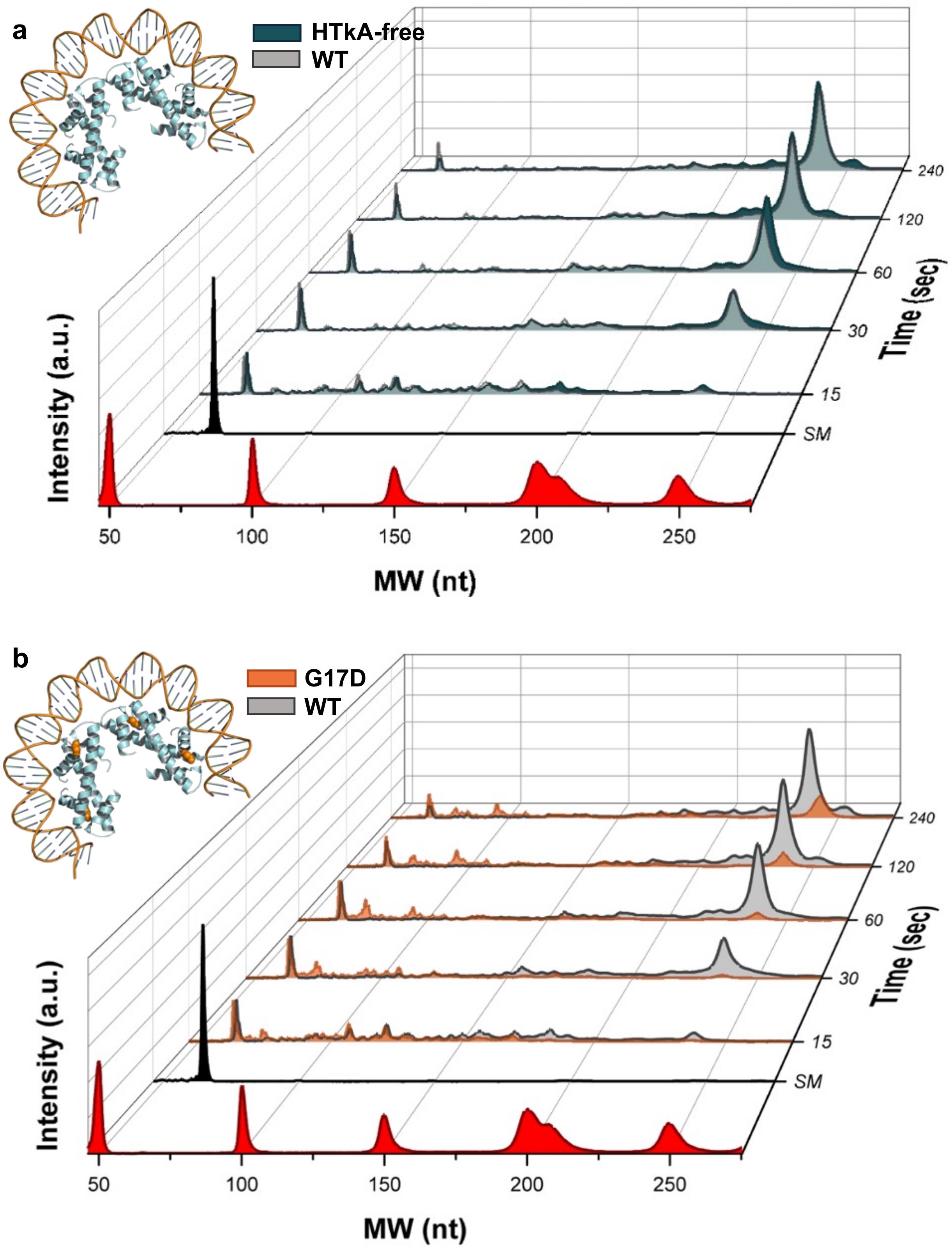
a
HTkA-free
WT
Intensity (a.u.)
MW (nt)
Time (sec)
SM
15
30
60
120
240
50
100
150
200
250
b
G17D
WT
Intensity (a.u.)
MW (nt)
Time (sec)
SM
15
30
60
120
240
50
100
150
200
250

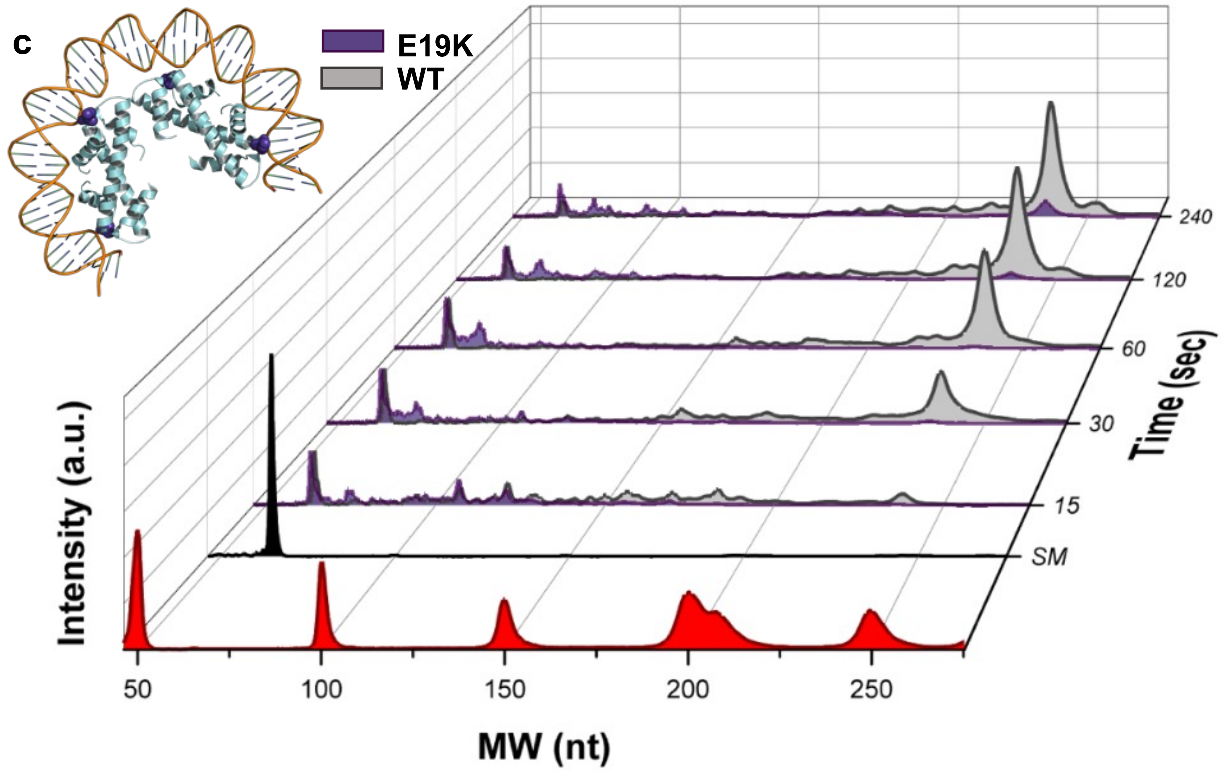
c
E19K
WT
Intensity (a.u.)
240
120
60
30
15
SM
Time (sec)
50
100
150
200
250
MW (nt)

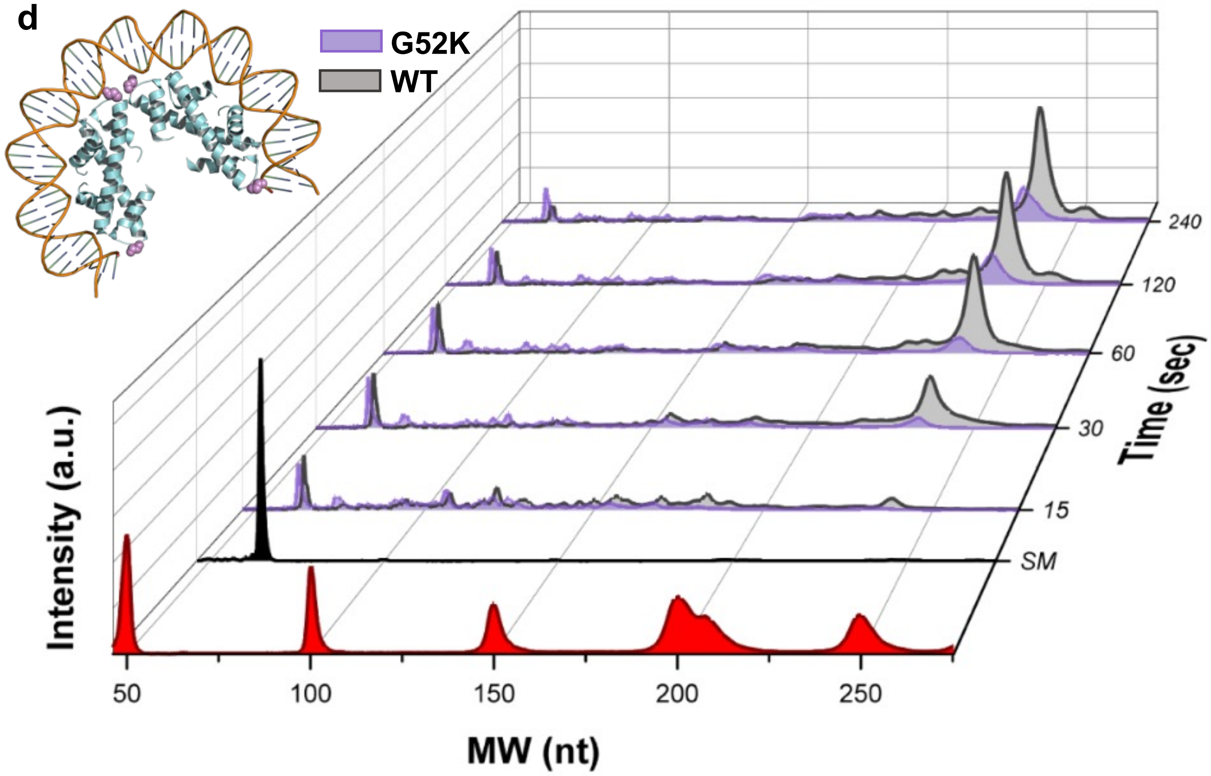
d
G52K
WT
Intensity (a.u.)
240
120
60
30
15
SM
Time (sec)
50
100
150
200
250
MW (nt)

29

e

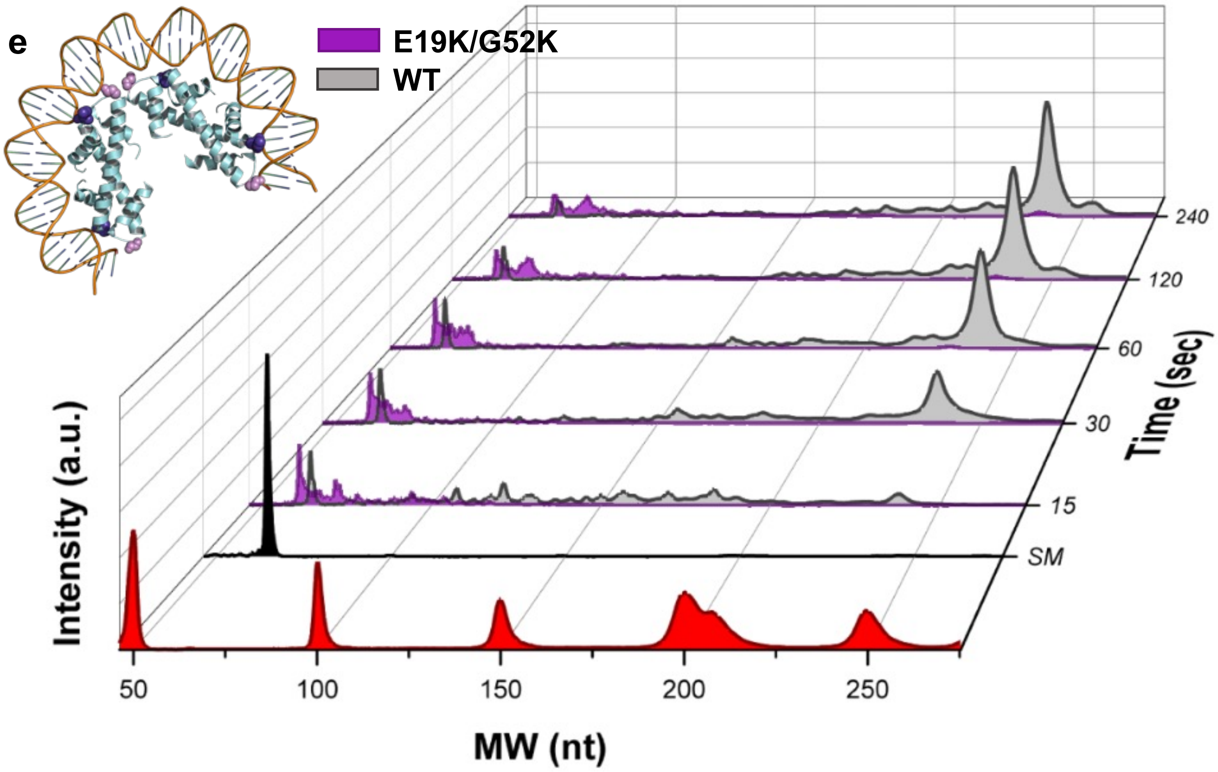

E19K/G52K
WT
Intensity (a.u.)
MW (nt)
50
100
150
200
250
Time (sec)
240
120
60
30
15
SM

f

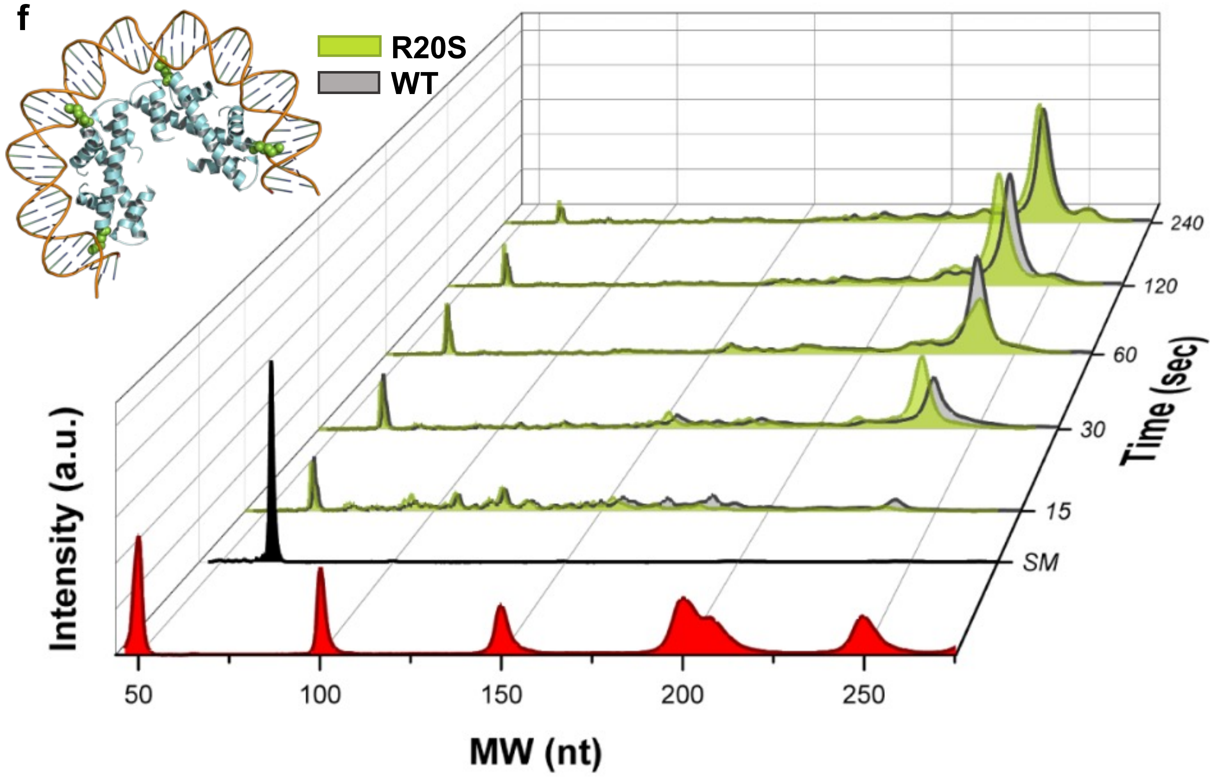

R20S
WT
Intensity (a.u.)
MW (nt)
50
100
150
200
250
Time (sec)
240
120
60
30
15
SM

30

g

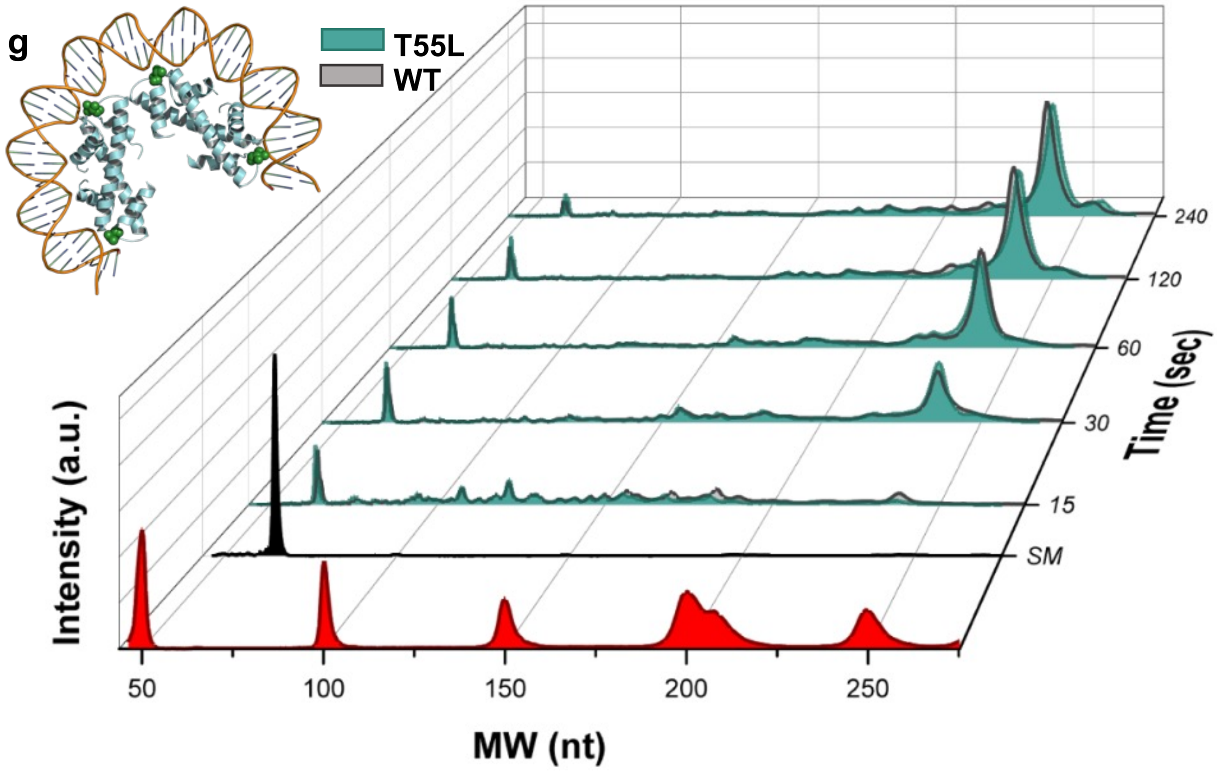
T55L
WT
Intensity (a.u.)
240
120
60
30
15
SM
Time (sec)
50
100
150
200
250
MW (nt)

h

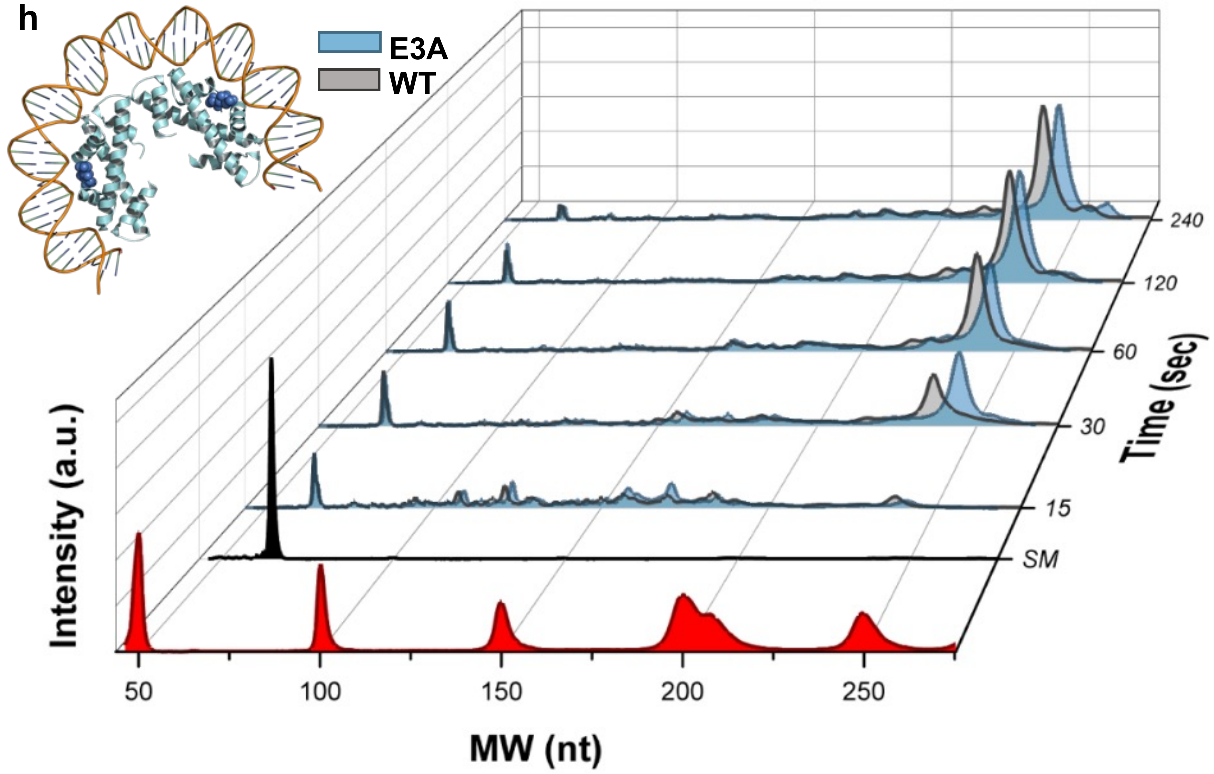
E3A
WT
Intensity (a.u.)
240
120
60
30
15
SM
Time (sec)
50
100
150
200
250
MW (nt)

31

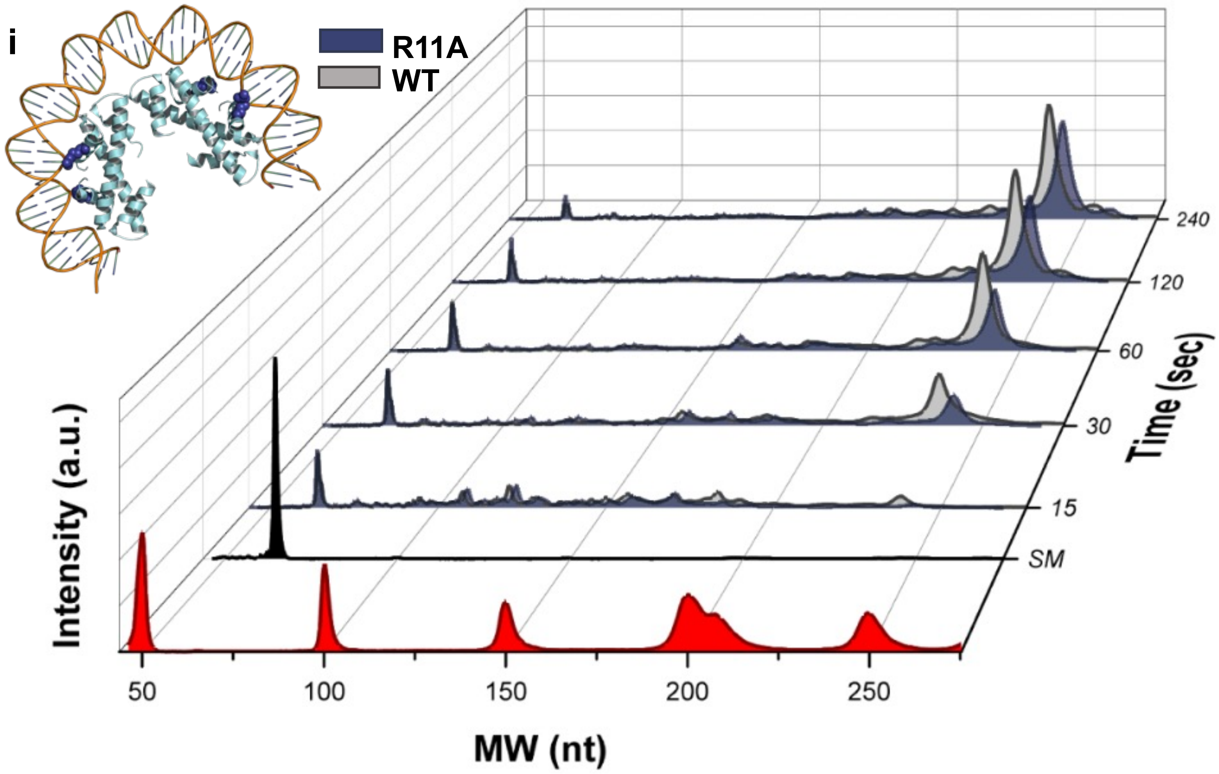
i
R11A
WT
Intensity (a.u.)
MW (nt)
50
100
150
200
250
Time (sec)
240
120
60
30
15
SM

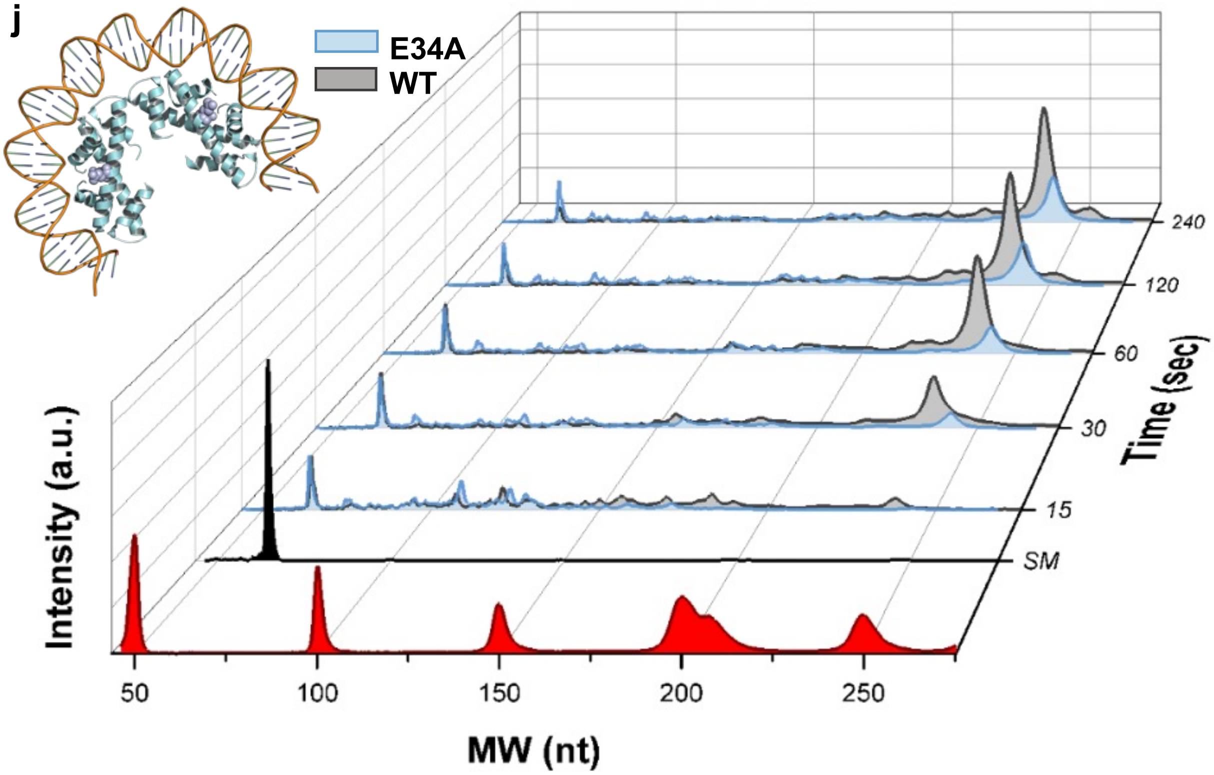
j
E34A
WT
Intensity (a.u.)
MW (nt)
50
100
150
200
250
Time (sec)
240
120
60
30
15
SM

32

Supplementary Figure 2. TEC progression is differentially impacted by distinct archaeal histone-based chromatin landscapes.

Waterfall plots permit quantification of the distribution of nascent transcript lengths over time. The relative intensity of different transcript lengths was normalized to the sum of the counts in the starting material (SM) within each lane. Transcript abundance is compared for (**a**) histone-free (HTkA-free) and wildtype ($HTkA^{WT}$), or between (**b – j**) $HTkA^{WT}$ and $HTkA^{variants}$.

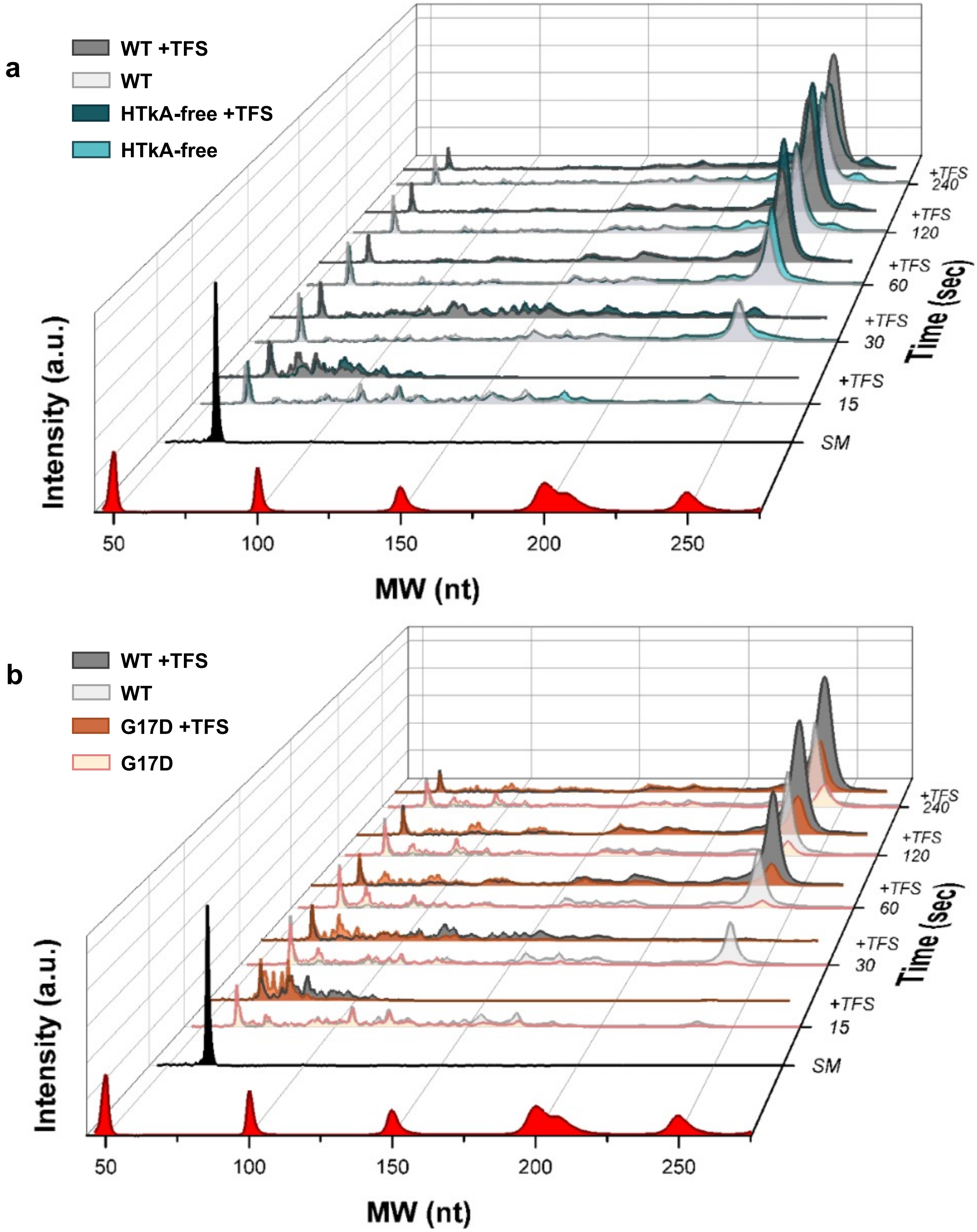
a
WT +TFS
WT
HTkA-free +TFS
HTkA-free
Intensity (a.u.)
MW (nt)
50
100
150
200
250
Time (sec)
SM
+TFS
15
+TFS
30
+TFS
60
+TFS
120
+TFS
240
b
WT +TFS
WT
G17D +TFS
G17D
Intensity (a.u.)
MW (nt)
50
100
150
200
250
Time (sec)
SM
+TFS
15
+TFS
30
+TFS
60
+TFS
120
+TFS
240

40

c

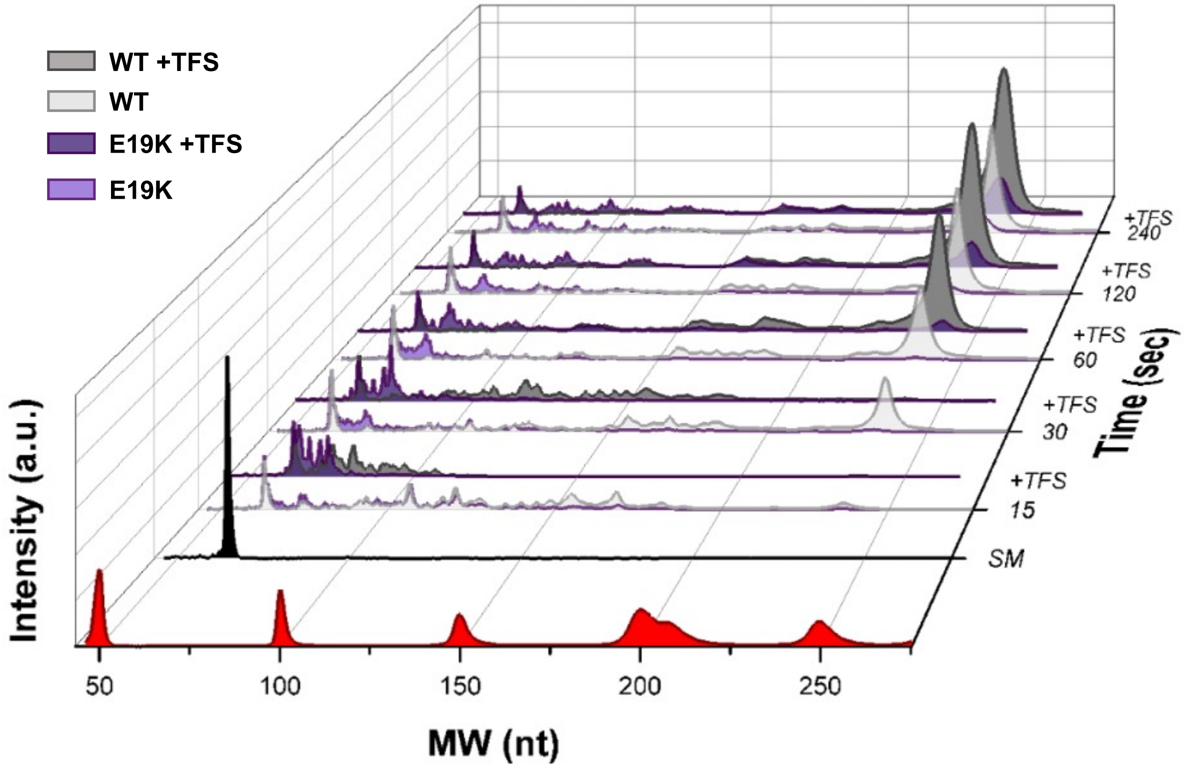
WT +TFS
WT
E19K +TFS
E19K
Intensity (a.u.)
50
100
150
200
250
MW (nt)
+TFS
240
+TFS
120
+TFS
60
+TFS
30
+TFS
15
SM
Time (sec)

d

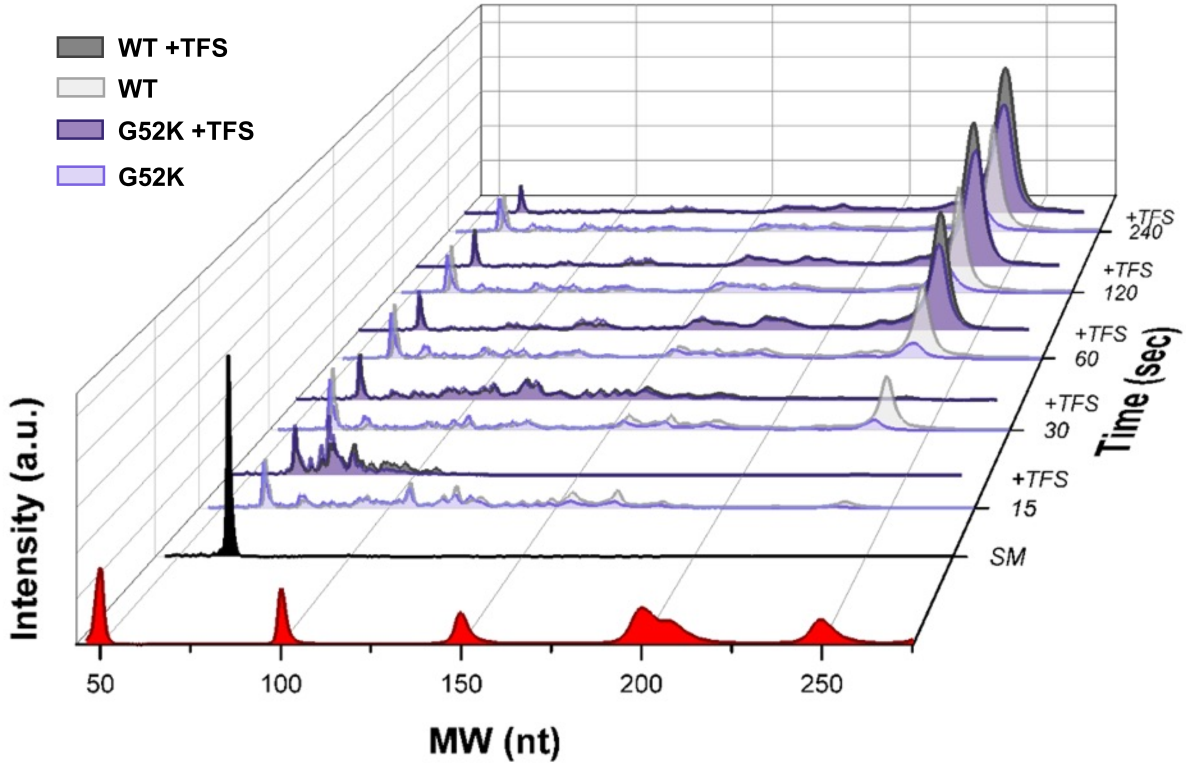
WT +TFS
WT
G52K +TFS
G52K
Intensity (a.u.)
50
100
150
200
250
MW (nt)
+TFS
240
+TFS
120
+TFS
60
+TFS
30
+TFS
15
SM
Time (sec)

41

e

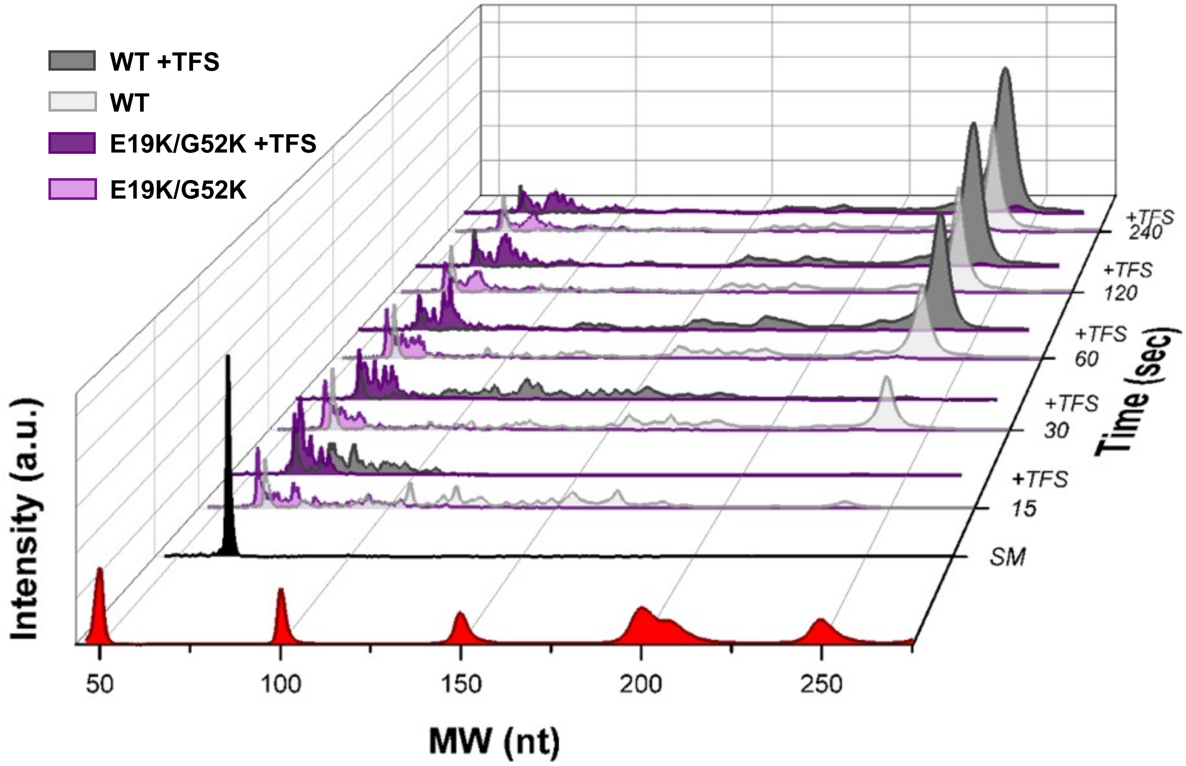
WT +TFS
WT
E19K/G52K +TFS
E19K/G52K
Intensity (a.u.)
50
100
150
200
250
MW (nt)
Time (sec)
SM
+TFS
15
+TFS
30
+TFS
60
+TFS
120
+TFS
240

f

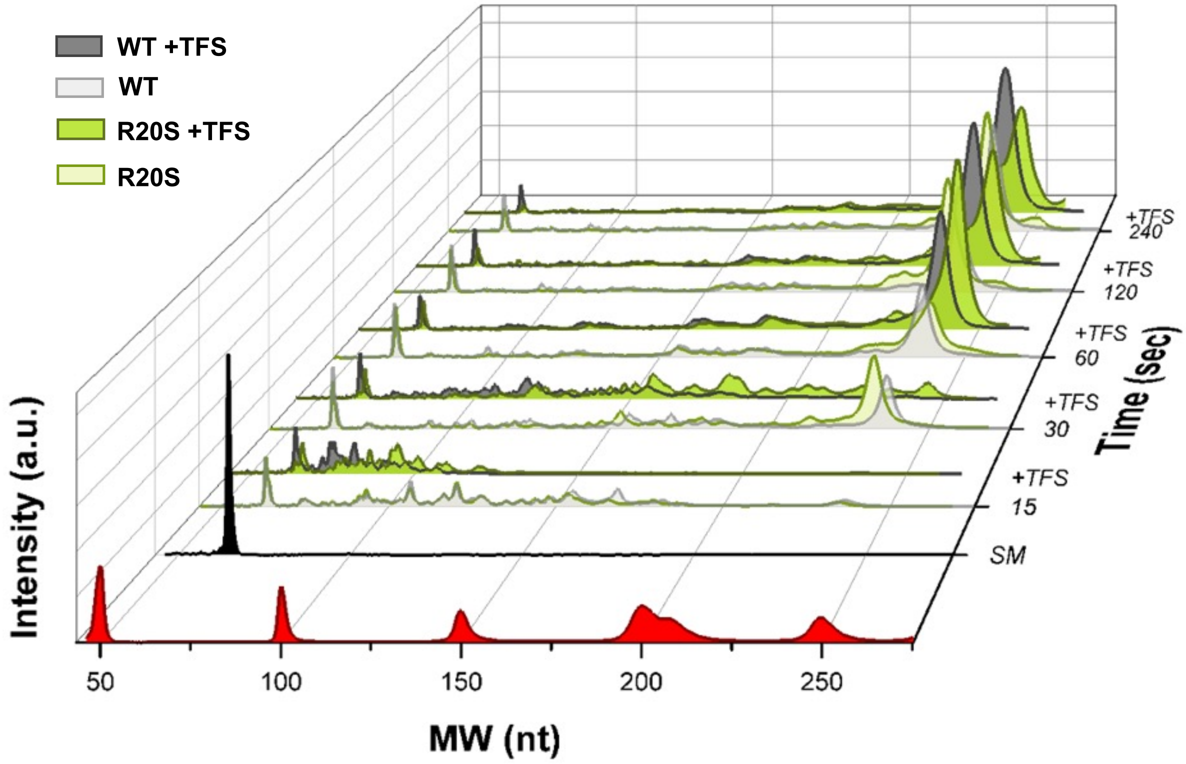
WT +TFS
WT
R20S +TFS
R20S
Intensity (a.u.)
50
100
150
200
250
MW (nt)
Time (sec)
SM
+TFS
15
+TFS
30
+TFS
60
+TFS
120
+TFS
240

42

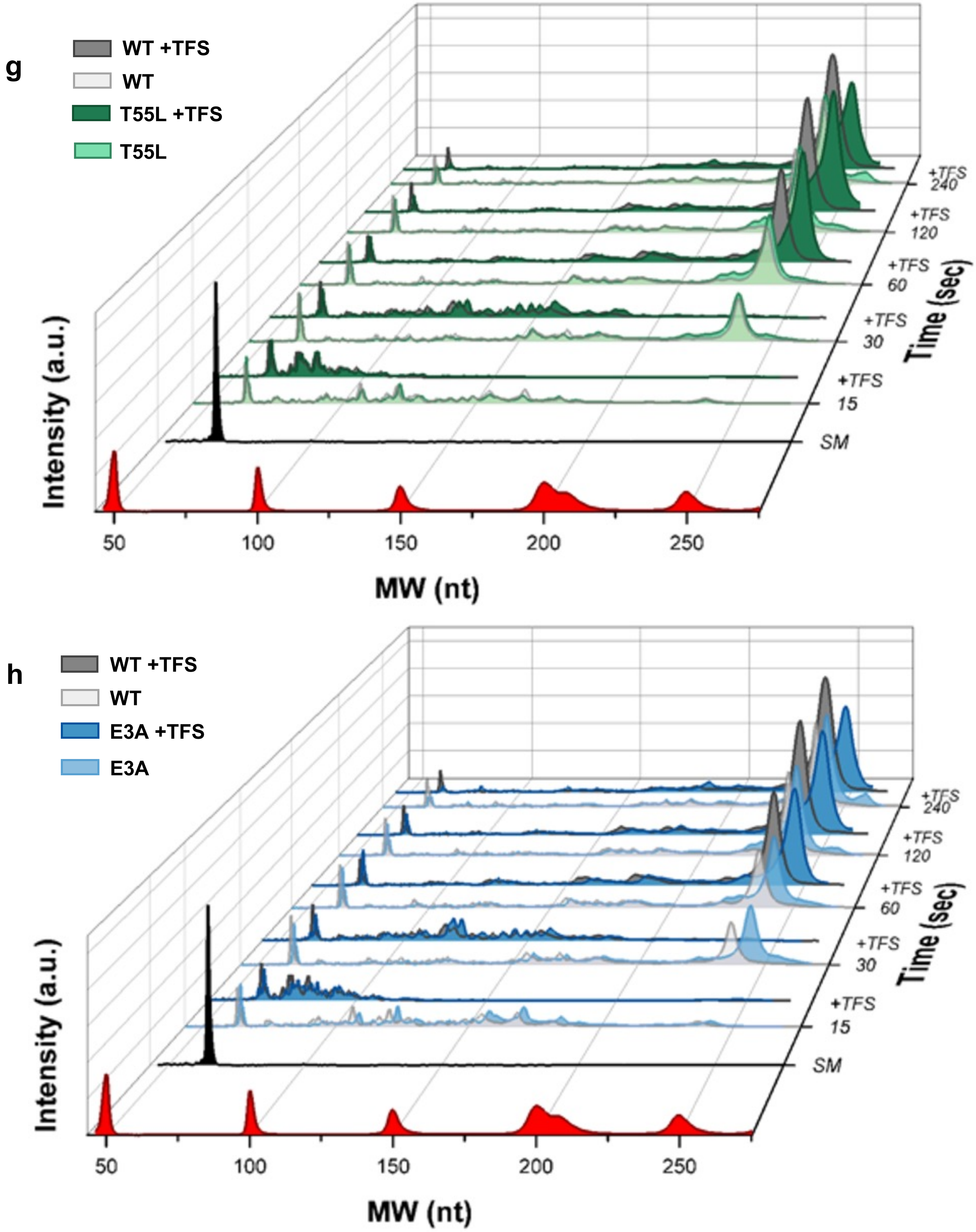
g
WT +TFS
WT
T55L +TFS
T55L
Intensity (a.u.)
MW (nt)
50
100
150
200
250
Time (sec)
SM
+TFS
15
+TFS
30
+TFS
60
+TFS
120
+TFS
240
h
WT +TFS
WT
E3A +TFS
E3A
Intensity (a.u.)
MW (nt)
50
100
150
200
250
Time (sec)
SM
+TFS
15
+TFS
30
+TFS
60
+TFS
120
+TFS
240

i

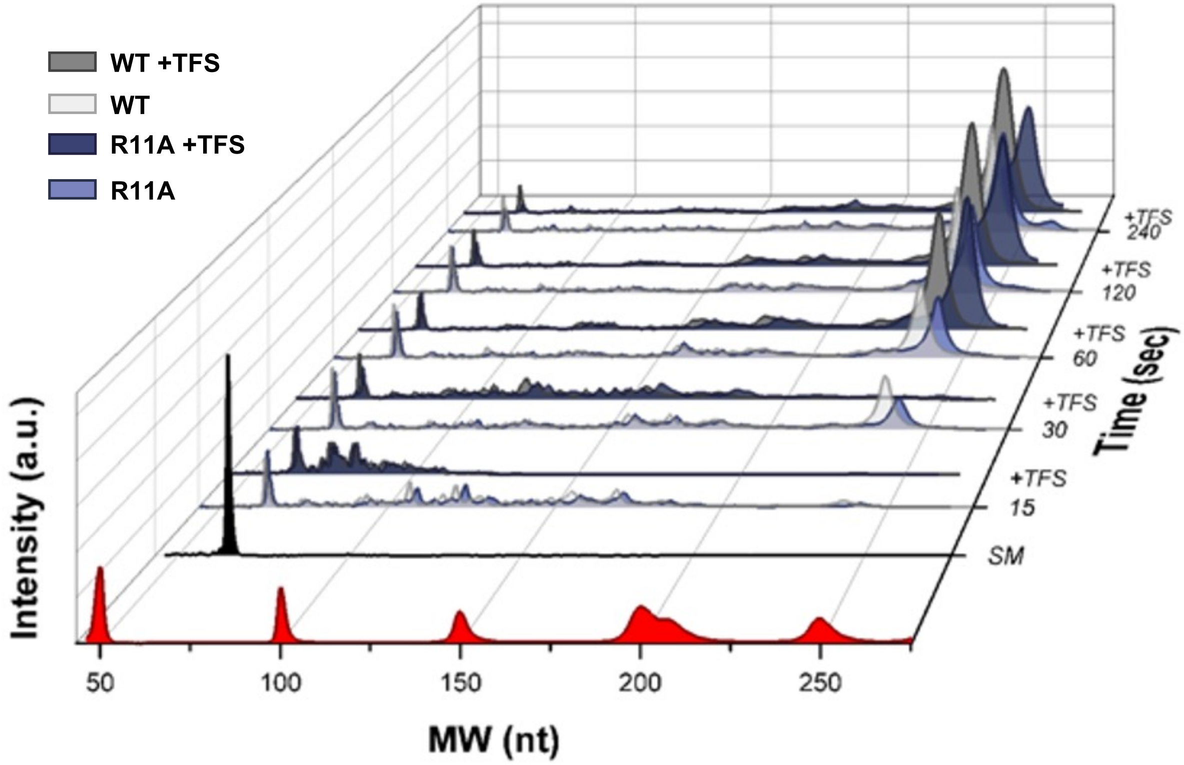
WT +TFS
WT
R11A +TFS
R11A
Intensity (a.u.)
MW (nt)
50
100
150
200
250
Time (sec)
SM
+TFS 15
+TFS 30
+TFS 60
+TFS 120
+TFS 240

j

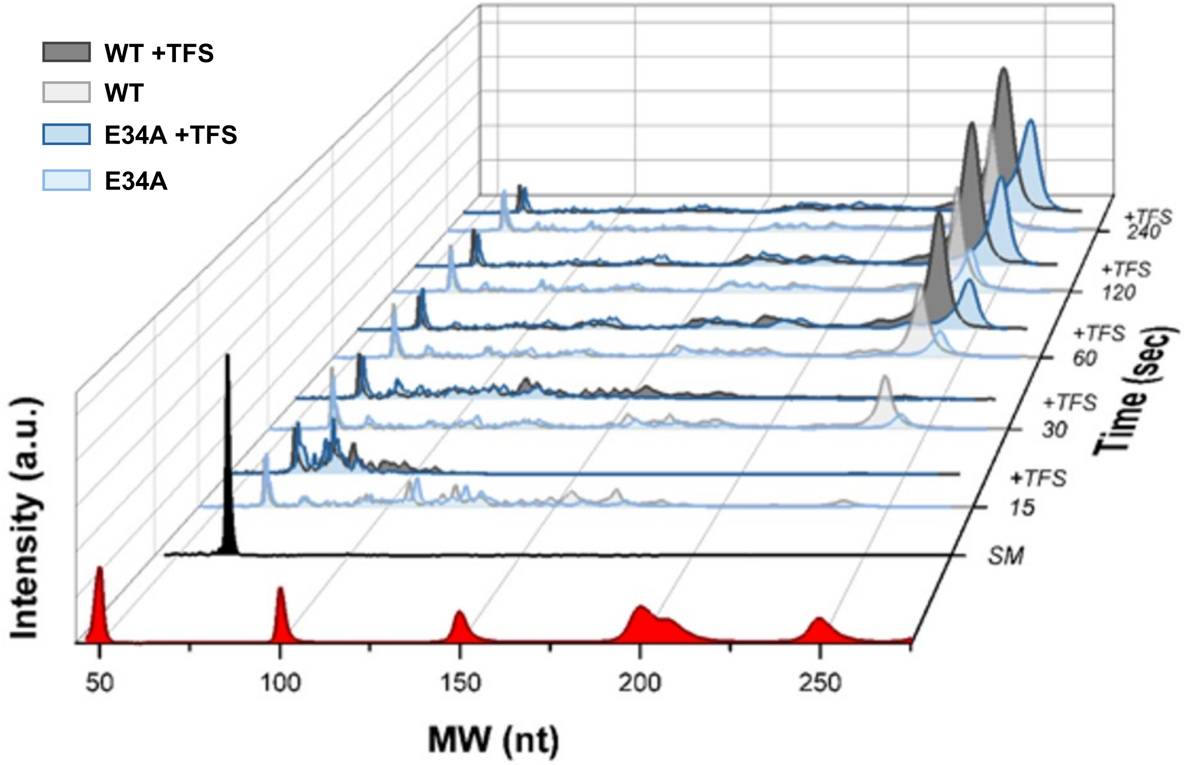
WT +TFS
WT
E34A +TFS
E34A
Intensity (a.u.)
MW (nt)
50
100
150
200
250
Time (sec)
SM
+TFS 15
+TFS 30
+TFS 60
+TFS 120
+TFS 240

<u>Supplementary Figure 3. 3D representation of TEC progression in a chromatin environment with and without TFS.</u>

(**a – j**) Waterfall plots permit quantification of the distribution of nascent transcript lengths over time in the presence or absence of TFS. The relative intensity of different transcript lengths was normalized to the sum of the counts in the starting material (SM) within each lane. Transcript abundance is compared for histone-free, $HTkA^{WT}$, and $HTkA^{variant}$ landscapes in the presence and absence of TFS.

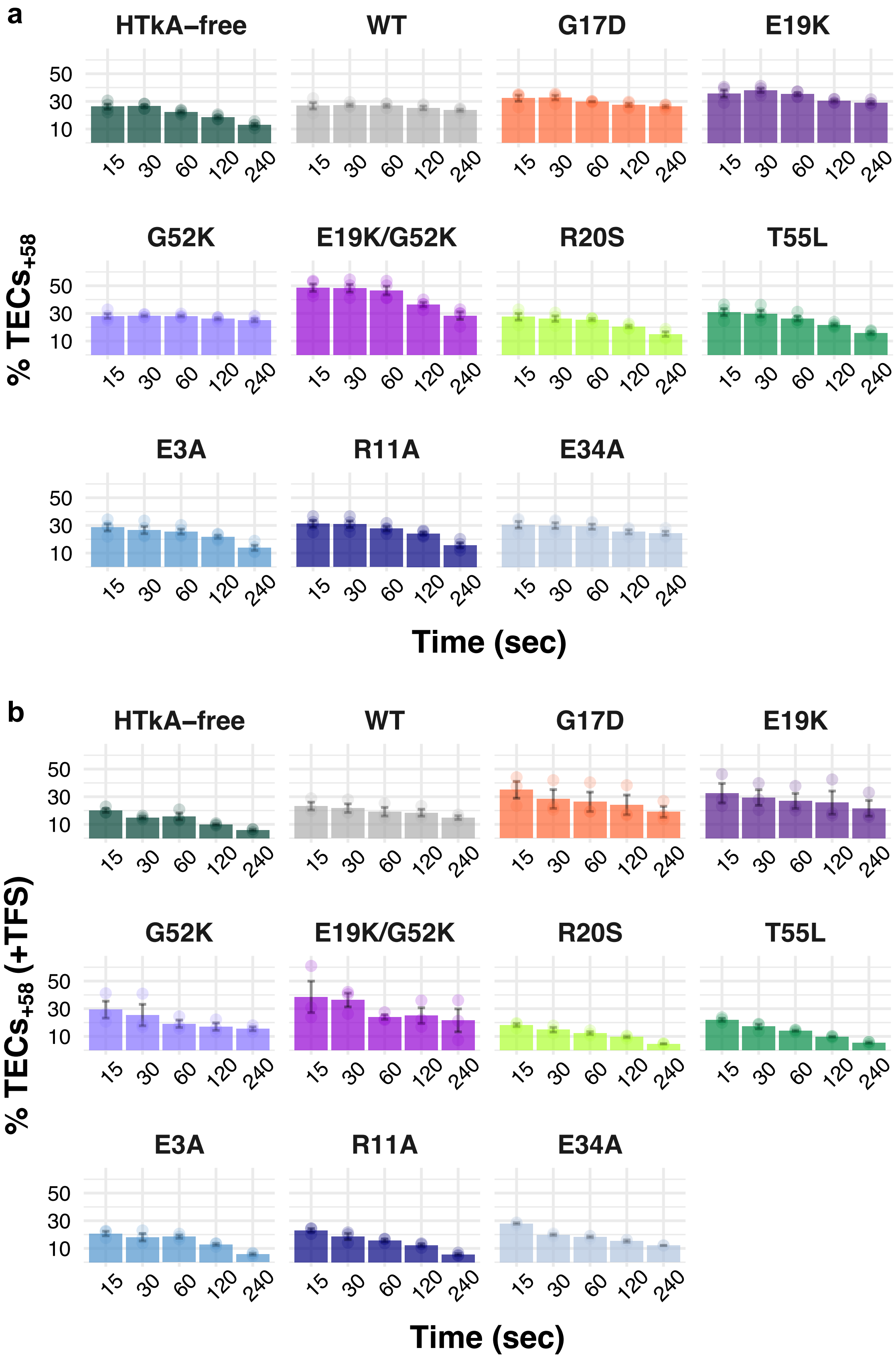

a
HTkA–free
WT
G17D
E19K
G52K
E19K/G52K
R20S
T55L
E3A
R11A
E34A
% TECs+58
Time (sec)
b
% TECs+58 (+TFS)

54

Supplementary Figure 4. TFS modestly improves transcription restart of TECs$_{+58}$.

(**a**) The percentage of TECs at position +58 nt decreases as TECs escape into active elongation. (**b**) Addition of TFS increases the rate of escape from +58 implying that some TECs were backtracked and could be stimulated into active elongation through TFS rescue. Average with standard error of n = 4 (**a**) and n = 3 (**b**) experiments is reported.

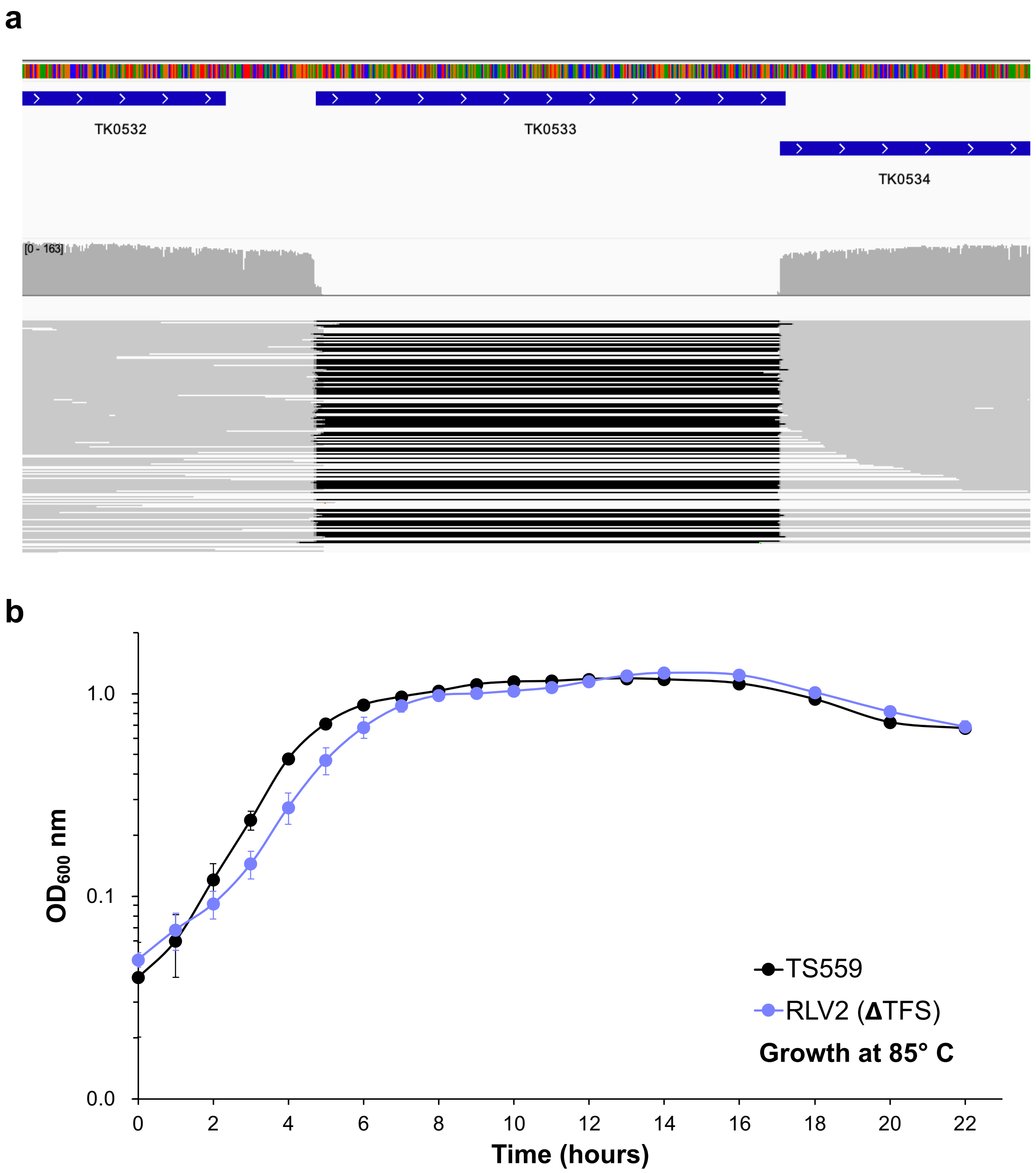

<u>**Supplementary Figure 5. TFS is not essential, and deletion has modest impacts for *T. kodakarensis* in optimal conditions.**</u>

(**a**) TK0533, encoding for TFS, was successfully deleted from the *T. kodakarensis* (TS559 – parental strain) genome. The exact endpoints of the deletion were confirmed via >100x whole

genome sequencing (WGS). (**b**) *T. kodakarensis* strains TS559 (parental) and RLV2 (ΔTK0533) grow nearly identically at 85˚C. Error bars represent the SD from biological triplicate cultures.

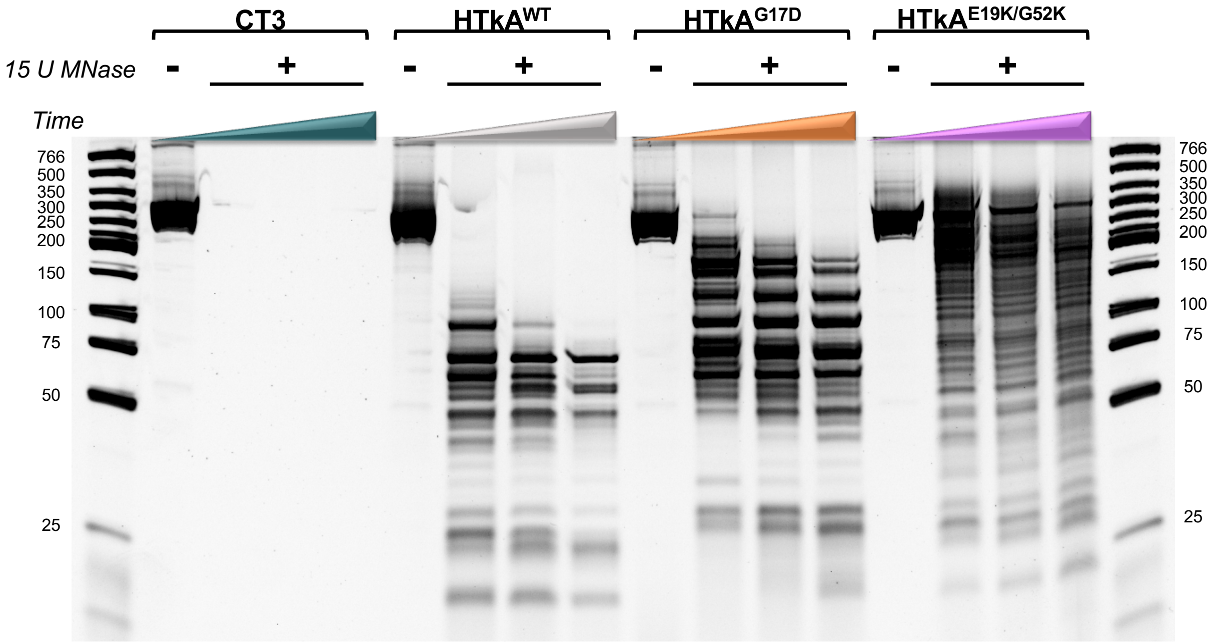

Supplementary Figure 6. Single residue substitutions alter the 3D histone-based chromatin structure.

MNase digestion of the DNA template (CT3 – Fig. 1a) used in our *in vitro* assays without and with HTkA^{WT}, HTkA^{G17D}, and HTkA$^{E19K/G52K}$. Digestion reactions were incubated for 0, 3, 6 and 12 minutes and contained 67 nM CT3 template, 20 μg histone proteins, and 15 U MNase. The purified fragments were run on a 10% TBE-urea polyacrylamide gel. Digested DNA fragments provide molecular weight standards.

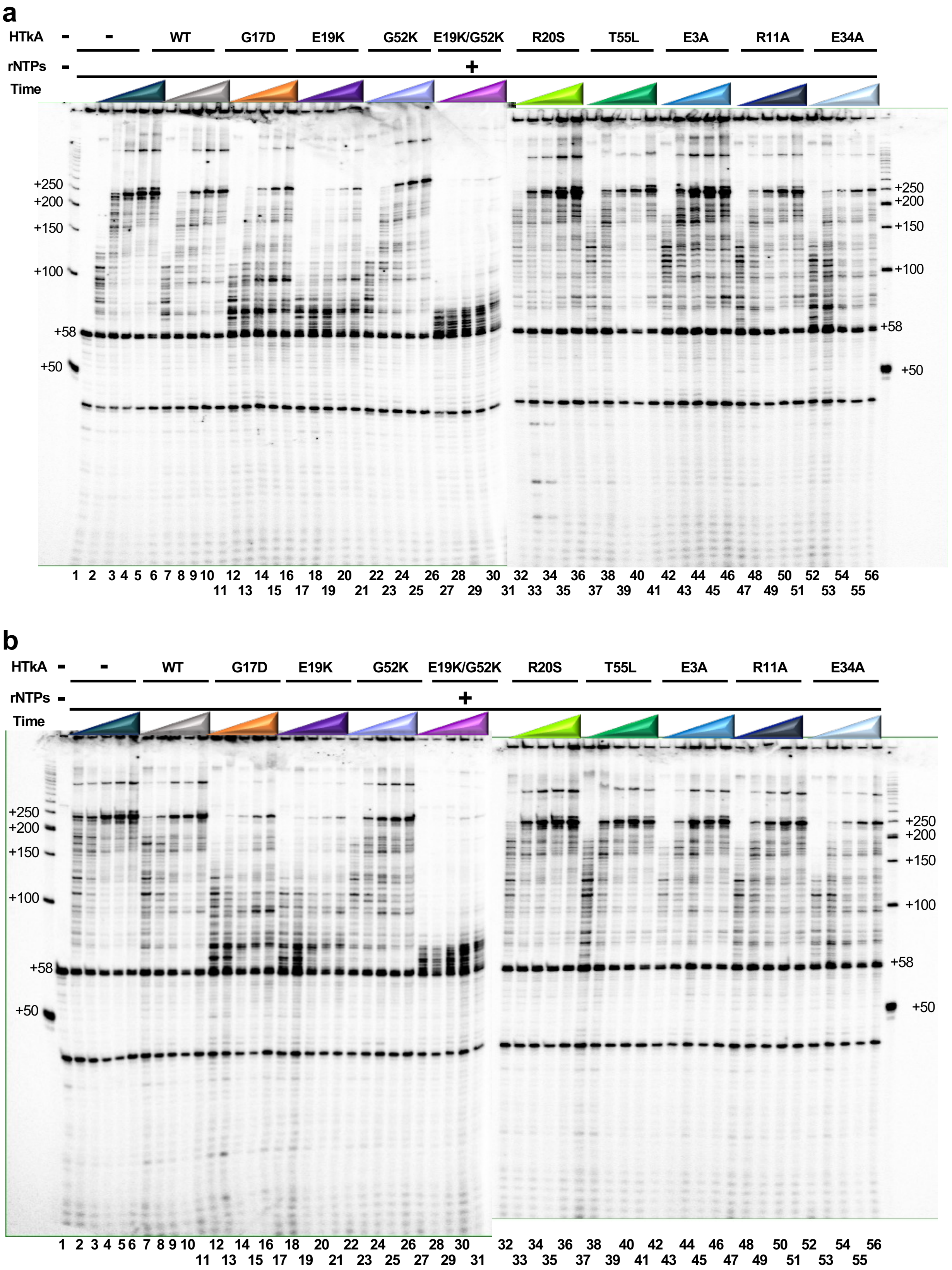
a
HTkA - - WT G17D E19K G52K E19K/G52K R20S T55L E3A R11A E34A
rNTPs - +
Time
+250
+200
+150
+100
+58
+50
1 2 3 4 5 6 7 8 9 10 11 12 13 14 15 16 17 18 19 20 21 22 23 24 25 26 27 28 29 30 31 32 33 34 35 36 37 38 39 40 41 42 43 44 45 46 47 48 49 50 51 52 53 54 55 56
b
HTkA - - WT G17D E19K G52K E19K/G52K R20S T55L E3A R11A E34A
rNTPs - +
Time
+250
+200
+150
+100
+58
+50
1 2 3 4 5 6 7 8 9 10 11 12 13 14 15 16 17 18 19 20 21 22 23 24 25 26 27 28 29 30 31 32 33 34 35 36 37 38 39 40 41 42 43 44 45 46 47 48 49 50 51 52 53 54 55 56

c

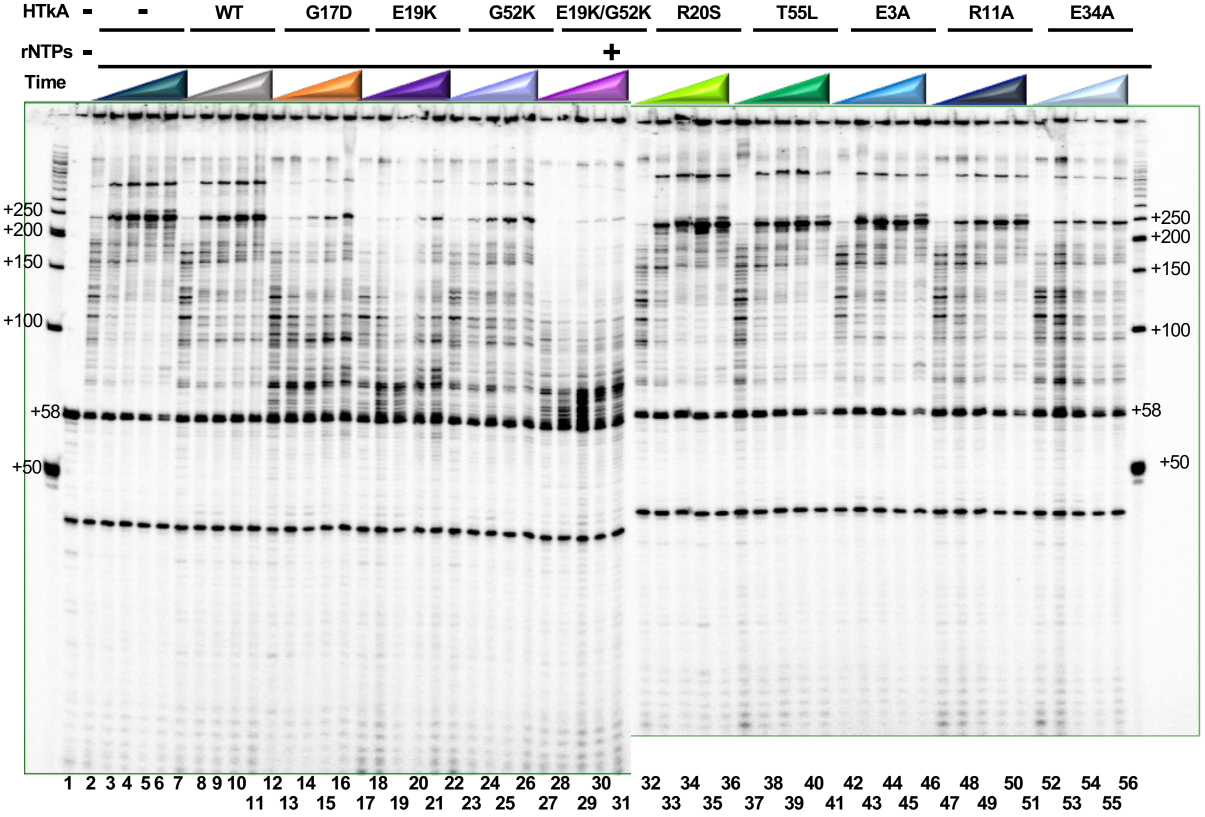
HTkA - - WT G17D E19K G52K E19K/G52K R20S T55L E3A R11A E34A
rNTPs - +
Time
+250
+200
+150
+100
+58
+50
1 2 3 4 5 6 7 8 9 10 12 14 16 18 20 22 24 26 28 30 32 34 36 38 40 42 44 46 48 50 52 54 56
11 13 15 17 19 21 23 25 27 29 31 33 35 37 39 41 43 45 47 49 51 53 55

d

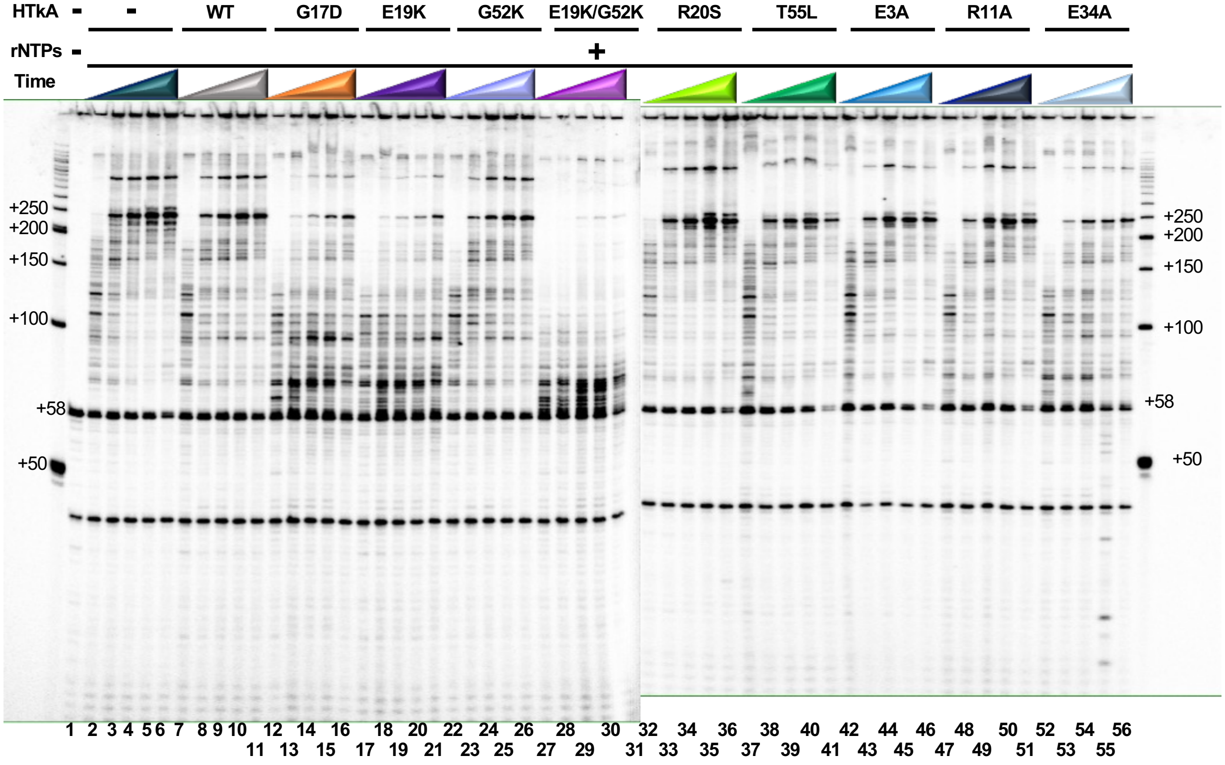
HTkA - - WT G17D E19K G52K E19K/G52K R20S T55L E3A R11A E34A
rNTPs - +
Time
+250
+200
+150
+100
+58
+50
1 2 3 4 5 6 7 8 9 10 12 14 16 18 20 22 24 26 28 30 32 34 36 38 40 42 44 46 48 50 52 54 56
11 13 15 17 19 21 23 25 27 29 31 33 35 37 39 41 43 45 47 49 51 53 55

e

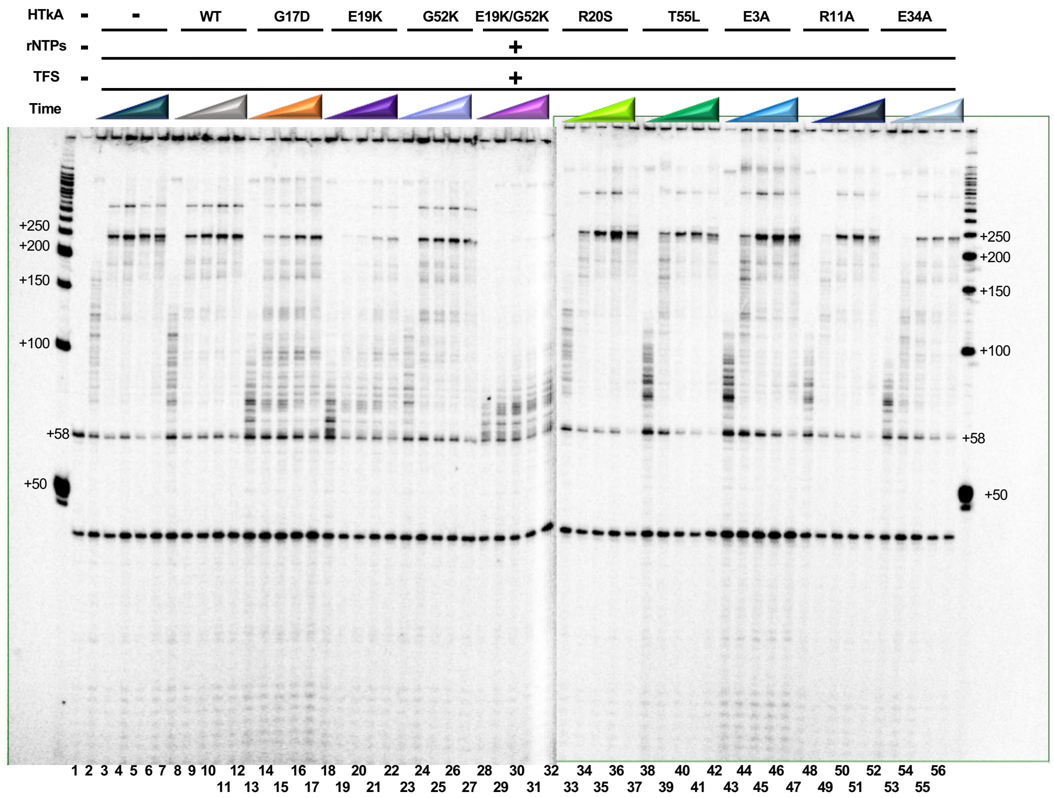
HTkA - - WT G17D E19K G52K E19K/G52K R20S T55L E3A R11A E34A
rNTPs - +
TFS - +
Time
+250
+200
+150
+100
+58
+50
1 2 3 4 5 6 7 8 9 10 11 12 13 14 15 16 17 18 19 20 21 22 23 24 25 26 27 28 29 30 31 32 33 34 35 36 37 38 39 40 41 42 43 44 45 46 47 48 49 50 51 52 53 54 55 56

f

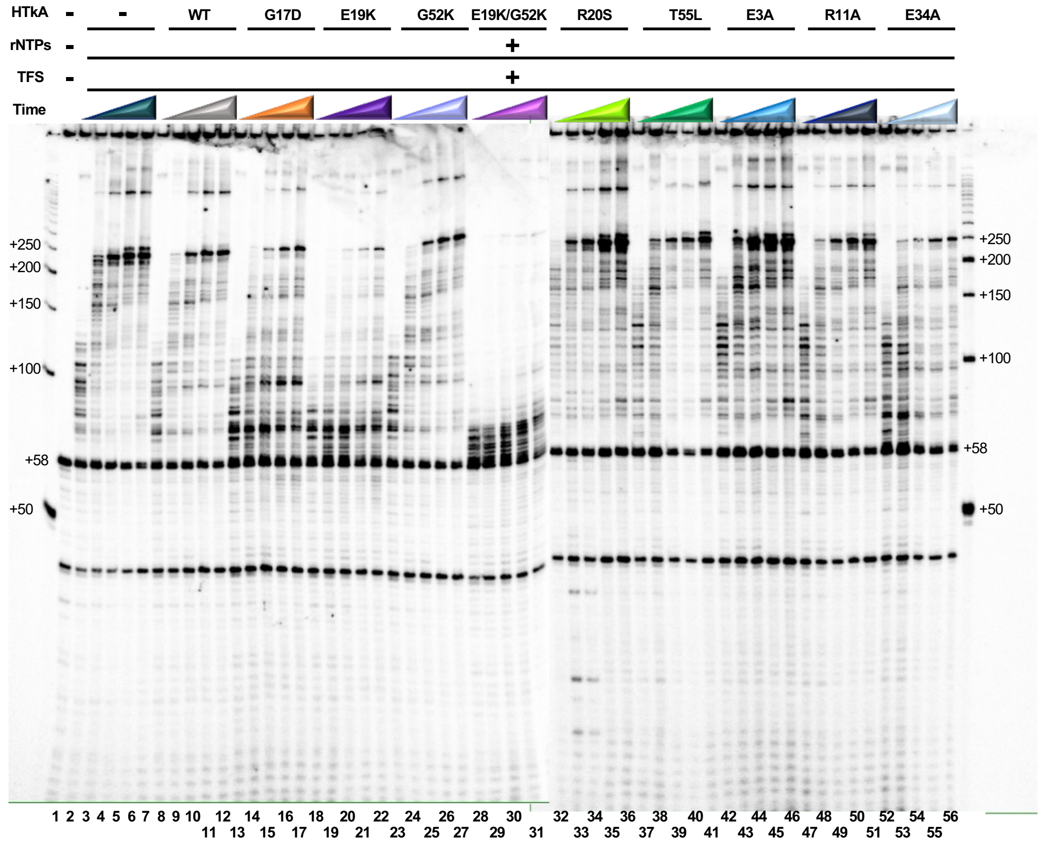
HTkA - - WT G17D E19K G52K E19K/G52K R20S T55L E3A R11A E34A
rNTPs - +
TFS - +
Time
+250
+200
+150
+100
+58
+50
1 2 3 4 5 6 7 8 9 10 11 12 13 14 15 16 17 18 19 20 21 22 23 24 25 26 27 28 29 30 31 32 33 34 35 36 37 38 39 40 41 42 43 44 45 46 47 48 49 50 51 52 53 54 55 56

81

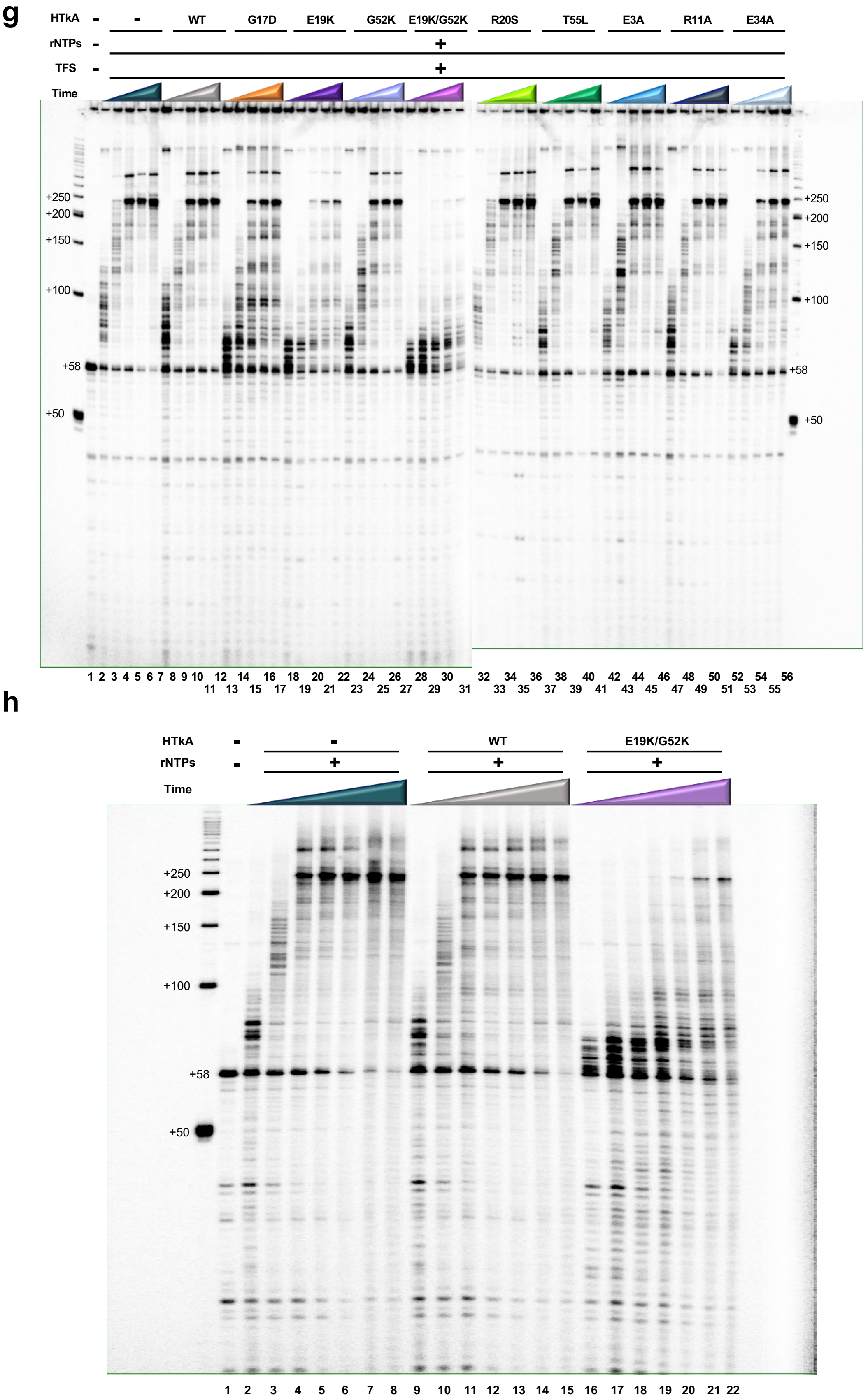
g
HTkA
rNTPs
TFS
Time
-
WT
G17D
E19K
G52K
E19K/G52K
R20S
T55L
E3A
R11A
E34A
+
+250
+200
+150
+100
+58
+50
h
HTkA
rNTPs
Time
-
WT
E19K/G52K
+
+250
+200
+150
+100
+58
+50
1 2 3 4 5 6 7 8 9 10 11 12 13 14 15 16 17 18 19 20 21 22

i

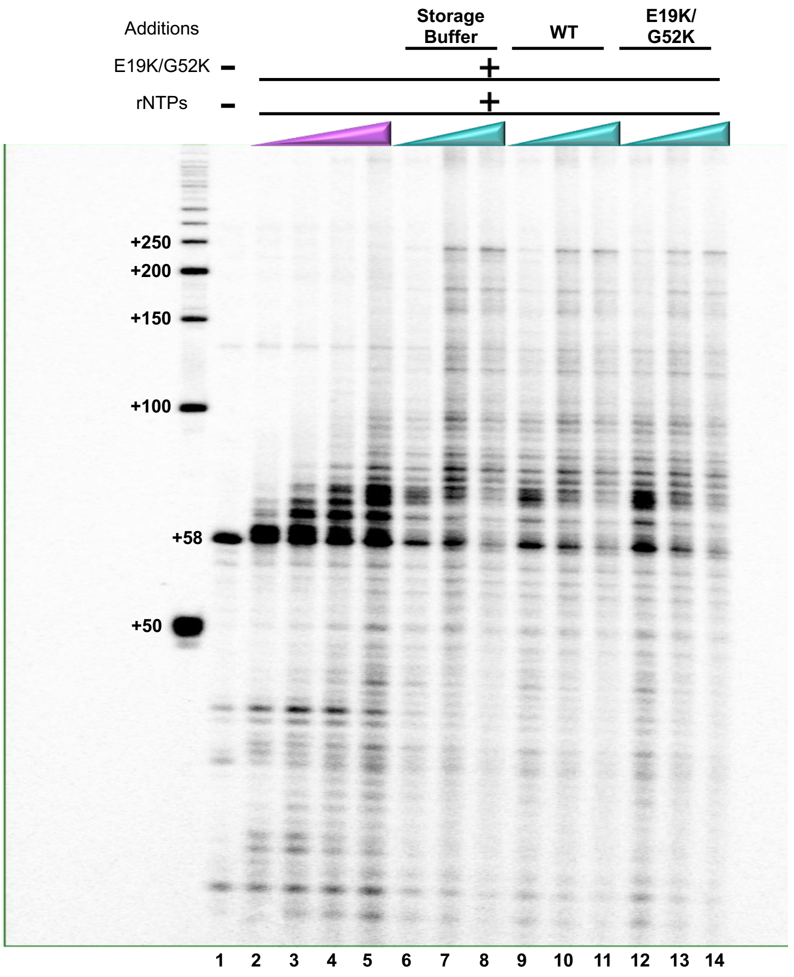
Additions
Storage Buffer
WT
E19K/ G52K
E19K/G52K
rNTPs
+250
+200
+150
+100
+58
+50
1 2 3 4 5 6 7 8 9 10 11 12 13 14

j

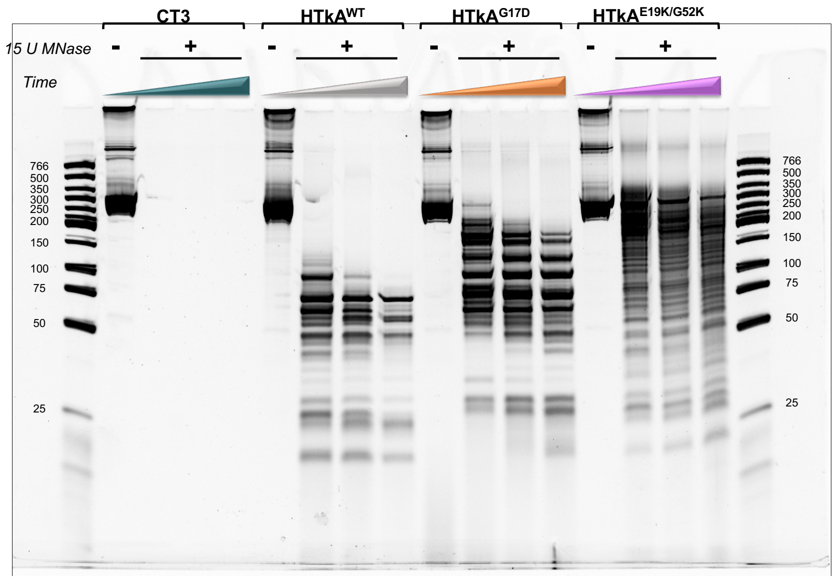
CT3
HTkAWT
HTkAG17D
HTkAE19K/G52K
15 U MNase
Time
766
500
350
300
250
200
150
100
75
50
25

k

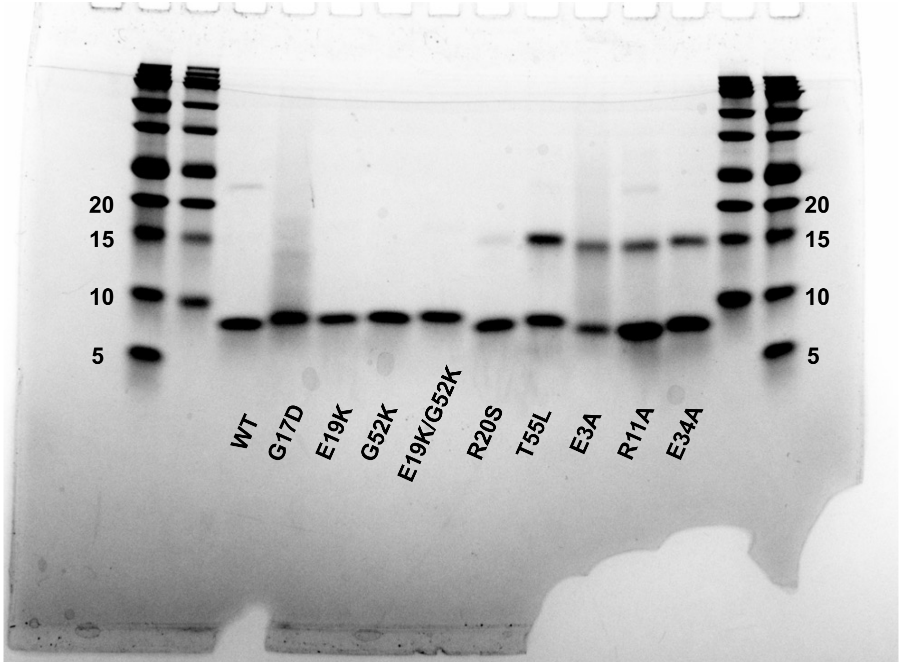
20
15
10
5
WT
G17D
E19K
G52K
E19K/G52K
R20S
T55L
E3A
R11A
E34A
20
15
10
5

l

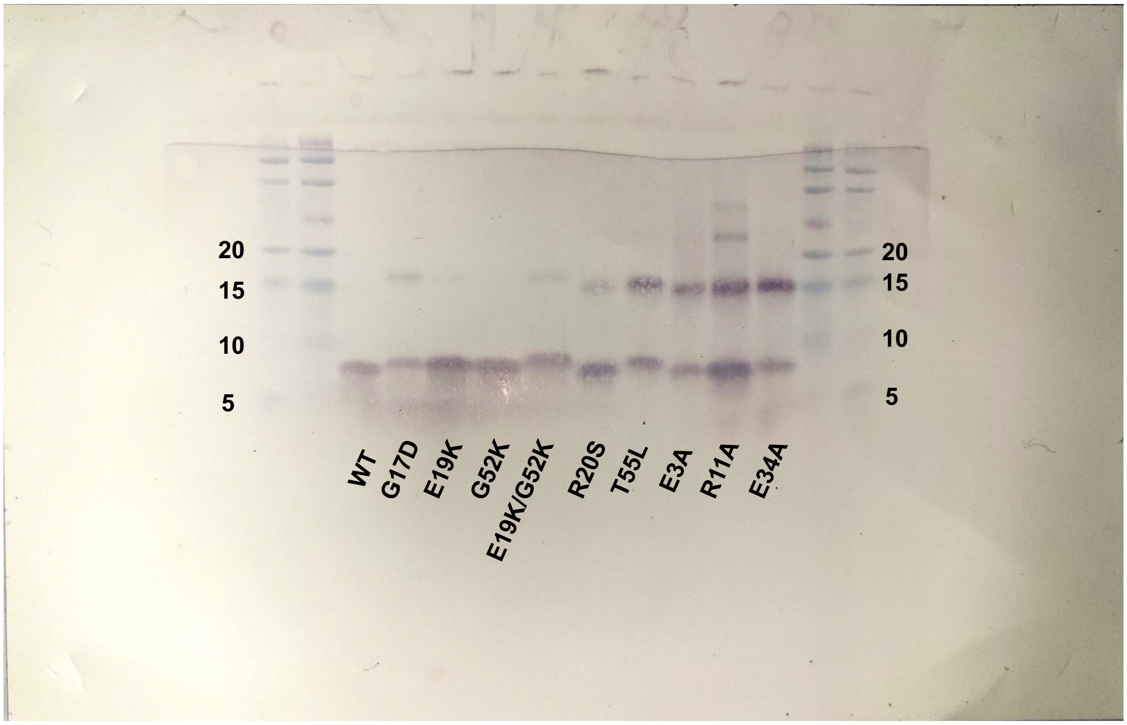
20
15
10
5
WT
G17D
E19K
G52K
E19K/G52K
R20S
T55L
E3A
R11A
E34A
20
15
10
5

Supplementary Figure 7. Unmodified gels and Western blot from representative images.

Unedited and uncropped gels for Fig. 1c (**a – d**), Fig. 4a (**e – g**), Fig. 3a (**h**), Fig. 3e (**i**), Supplementary Fig. 6 (**j**), Supplementary Figs. 1b & 1c (**k & l**).
