# Supplementary material for: Archaeal histone-based chromatin structures regulate transcription elongation rates
Source: Commun Biol. 2024 Feb 27;7:236. doi: 10.1038/s42003-024-05928-w (PMC10899632; doi:10.1038/s42003-024-05928-w)
Supplement: Supplementary file 3 — Description of Additional Supplementary Files [file 42003_2024_5928_MOESM3_ESM.pdf]

Description of Additional Supplementary Files

File name: Supplementary Data 1

Description: Excel file containing the values and calculations to determine the average elongation rates.

File name: Supplementary Data 2

Description: HTML file that details the code utilized to obtain the in-silico approach to determining the free energy of histone variants.
