# Supplementary material for: Archaeal histone-based chromatin structures regulate transcription elongation rates
Source: Commun Biol. 2024 Feb 27;7:236. doi: 10.1038/s42003-024-05928-w (PMC10899632; doi:10.1038/s42003-024-05928-w)
Supplement: Supplementary file 5 — Supplementary Data 2 [file 42003_2024_5928_MOESM5_ESM.html]

PyRosetta\_HTkA\_modeling


## Archaeal histone-based chromatin structures regulate transcription elongation rates.¶

#### Wenck, B. et al. (2023)¶

In [1]:

```
#!pip install pyrosettacolabsetup #only needs to be run once
import pyrosettacolabsetup; pyrosettacolabsetup.install_pyrosetta()
import pyrosetta; pyrosetta.init()
```

```
PyRosetta-4 2022 [Rosetta PyRosetta4.Release.python37.mac 2022.23+release.f1e0f6d7bf74728d46e42a39ffab1eedf8c1c936 2022-06-09T16:58:25] retrieved from: http://www.pyrosetta.org
(C) Copyright Rosetta Commons Member Institutions. Created in JHU by Sergey Lyskov and PyRosetta Team.
core.init: Checking for fconfig files in pwd and ./rosetta/flags
core.init: Rosetta version: PyRosetta4.Release.python37.mac r321 2022.23+release.f1e0f6d7bf7 f1e0f6d7bf74728d46e42a39ffab1eedf8c1c936 http://www.pyrosetta.org 2022-06-09T16:58:25
core.init: command: PyRosetta -ex1 -ex2aro -database /Users/breewenck/.local/lib/python3.7/site-packages/pyrosetta/database
basic.random.init_random_generator: 'RNG device' seed mode, using '/dev/urandom', seed=1750546524 seed_offset=0 real_seed=1750546524
basic.random.init_random_generator: RandomGenerator:init: Normal mode, seed=1750546524 RG_type=mt19937
```

In [2]:

```
from pyrosetta import *
init()
```

```
PyRosetta-4 2022 [Rosetta PyRosetta4.Release.python37.mac 2022.23+release.f1e0f6d7bf74728d46e42a39ffab1eedf8c1c936 2022-06-09T16:58:25] retrieved from: http://www.pyrosetta.org
(C) Copyright Rosetta Commons Member Institutions. Created in JHU by Sergey Lyskov and PyRosetta Team.
core.init: Checking for fconfig files in pwd and ./rosetta/flags
core.init: Rosetta version: PyRosetta4.Release.python37.mac r321 2022.23+release.f1e0f6d7bf7 f1e0f6d7bf74728d46e42a39ffab1eedf8c1c936 http://www.pyrosetta.org 2022-06-09T16:58:25
core.init: command: PyRosetta -ex1 -ex2aro -database /Users/breewenck/.local/lib/python3.7/site-packages/pyrosetta/database
basic.random.init_random_generator: 'RNG device' seed mode, using '/dev/urandom', seed=-2081118736 seed_offset=0 real_seed=-2081118736
basic.random.init_random_generator: RandomGenerator:init: Normal mode, seed=-2081118736 RG_type=mt19937
```

### After importing PyRosetta, it is important to clean up the pdb structure.¶

In [3]:

```
from pyrosetta.toolbox import cleanATOM
cleanATOM("pdb/5T5K.pdb")
```

In [4]:

```
cpose = pose_from_pdb("pdb/5T5K.clean.pdb")
```

```
core.chemical.GlobalResidueTypeSet: Finished initializing fa_standard residue type set.  Created 985 residue types
core.chemical.GlobalResidueTypeSet: Total time to initialize 0.788638 seconds.
core.import_pose.import_pose: File 'pdb/5T5K.clean.pdb' automatically determined to be of type PDB
core.conformation.Conformation: [ WARNING ] missing heavyatom:  OXT on residue PHE:CtermProteinFull 67
core.conformation.Conformation: [ WARNING ] missing heavyatom:  OXT on residue LYS:CtermProteinFull 135
core.conformation.Conformation: [ WARNING ] missing heavyatom:  OXT on residue LYS:CtermProteinFull 203
core.conformation.Conformation: [ WARNING ] missing heavyatom:  OXT on residue PHE:CtermProteinFull 270
core.conformation.Conformation: [ WARNING ] missing heavyatom:  OXT on residue PHE:CtermProteinFull 337
core.conformation.Conformation: [ WARNING ] missing heavyatom:  OXT on residue PHE:CtermProteinFull 404
```

In [5]:

```
print(cpose.pdb_info())
```

```
PDB file name: pdb/5T5K.clean.pdb
 Pose Range  Chain    PDB Range  |   #Residues         #Atoms

0001 -- 0067    A 0001  -- 0067  |   0067 residues;    01076 atoms
0068 -- 0135    B 0001  -- 0068  |   0068 residues;    01098 atoms
0136 -- 0203    C 0001  -- 0068  |   0068 residues;    01098 atoms
0204 -- 0270    D 0001  -- 0067  |   0067 residues;    01076 atoms
0271 -- 0337    E 0001  -- 0067  |   0067 residues;    01076 atoms
0338 -- 0404    F 0001  -- 0067  |   0067 residues;    01076 atoms
0405 -- 0494    I 0001  -- 0090  |   0090 residues;    02859 atoms
0495 -- 0584    J 0001  -- 0090  |   0090 residues;    02862 atoms
                           TOTAL |   0584 residues;    12221 atoms
```

In [6]:

```
from pyrosetta.rosetta.protocols.loops import get_fa_scorefxn
sfxn = get_fa_scorefxn()
```

```
core.scoring.ScoreFunctionFactory: SCOREFUNCTION: ref2015
core.scoring.etable: Starting energy table calculation
core.scoring.etable: smooth_etable: changing atr/rep split to bottom of energy well
core.scoring.etable: smooth_etable: spline smoothing lj etables (maxdis = 6)
core.scoring.etable: smooth_etable: spline smoothing solvation etables (max_dis = 6)
core.scoring.etable: Finished calculating energy tables.
basic.io.database: Database file opened: scoring/score_functions/hbonds/ref2015_params/HBPoly1D.csv
basic.io.database: Database file opened: scoring/score_functions/hbonds/ref2015_params/HBFadeIntervals.csv
basic.io.database: Database file opened: scoring/score_functions/hbonds/ref2015_params/HBEval.csv
basic.io.database: Database file opened: scoring/score_functions/hbonds/ref2015_params/DonStrength.csv
basic.io.database: Database file opened: scoring/score_functions/hbonds/ref2015_params/AccStrength.csv
basic.io.database: Database file opened: scoring/score_functions/rama/fd/all.ramaProb
basic.io.database: Database file opened: scoring/score_functions/rama/fd/prepro.ramaProb
basic.io.database: Database file opened: scoring/score_functions/omega/omega_ppdep.all.txt
basic.io.database: Database file opened: scoring/score_functions/omega/omega_ppdep.gly.txt
basic.io.database: Database file opened: scoring/score_functions/omega/omega_ppdep.pro.txt
basic.io.database: Database file opened: scoring/score_functions/omega/omega_ppdep.valile.txt
basic.io.database: Database file opened: scoring/score_functions/P_AA_pp/P_AA
basic.io.database: Database file opened: scoring/score_functions/P_AA_pp/P_AA_n
core.scoring.P_AA: shapovalov_lib::shap_p_aa_pp_smooth_level of 1( aka low_smooth ) got activated.
basic.io.database: Database file opened: scoring/score_functions/P_AA_pp/shapovalov/10deg/kappa131/a20.prop
```

In [7]:

```
sfxn.show(cpose)
```

```
basic.io.database: Database file opened: scoring/score_functions/elec_cp_reps.dat
core.scoring.elec.util: Read 40 countpair representative atoms
core.pack.dunbrack.RotamerLibrary: shapovalov_lib_fixes_enable option is true.
core.pack.dunbrack.RotamerLibrary: shapovalov_lib::shap_dun10_smooth_level of 1( aka lowest_smooth ) got activated.
core.pack.dunbrack.RotamerLibrary: Binary rotamer library selected: /Users/breewenck/.local/lib/python3.7/site-packages/pyrosetta/database/rotamer/shapovalov/StpDwn_0-0-0/Dunbrack10.lib.bin
core.pack.dunbrack.RotamerLibrary: Using Dunbrack library binary file '/Users/breewenck/.local/lib/python3.7/site-packages/pyrosetta/database/rotamer/shapovalov/StpDwn_0-0-0/Dunbrack10.lib.bin'.
core.pack.dunbrack.RotamerLibrary: Dunbrack 2010 library took 0.326904 seconds to load from binary
core.scoring.ScoreFunction: 
------------------------------------------------------------
 Scores                       Weight   Raw Score Wghtd.Score
------------------------------------------------------------
 fa_atr                       1.000   -4746.768   -4746.768
 fa_rep                       0.550    1870.733    1028.903
 fa_sol                       1.000    4288.994    4288.994
 fa_intra_rep                 0.005    2799.361      13.997
 fa_intra_sol_xover4          1.000     534.147     534.147
 lk_ball_wtd                  1.000    -263.826    -263.826
 fa_elec                      1.000   -1101.836   -1101.836
 pro_close                    1.250       1.602       2.003
 hbond_sr_bb                  1.000    -221.674    -221.674
 hbond_lr_bb                  1.000     -20.688     -20.688
 hbond_bb_sc                  1.000     -12.331     -12.331
 hbond_sc                     1.000    -248.705    -248.705
 dslf_fa13                    1.250       0.000       0.000
 omega                        0.400      90.726      36.290
 fa_dun                       0.700    1224.117     856.882
 p_aa_pp                      0.600     -65.076     -39.046
 yhh_planarity                0.625       0.000       0.000
 ref                          1.000     136.169     136.169
 rama_prepro                  0.450     -26.286     -11.829
---------------------------------------------------
 Total weighted score:                      230.682
```

In [8]:

```
temp_pose = cpose.clone()
sfxn.show(temp_pose)
```

```
core.scoring.ScoreFunction: 
------------------------------------------------------------
 Scores                       Weight   Raw Score Wghtd.Score
------------------------------------------------------------
 fa_atr                       1.000   -4746.768   -4746.768
 fa_rep                       0.550    1870.733    1028.903
 fa_sol                       1.000    4288.994    4288.994
 fa_intra_rep                 0.005    2799.361      13.997
 fa_intra_sol_xover4          1.000     534.147     534.147
 lk_ball_wtd                  1.000    -263.826    -263.826
 fa_elec                      1.000   -1101.836   -1101.836
 pro_close                    1.250       1.602       2.003
 hbond_sr_bb                  1.000    -221.674    -221.674
 hbond_lr_bb                  1.000     -20.688     -20.688
 hbond_bb_sc                  1.000     -12.331     -12.331
 hbond_sc                     1.000    -248.705    -248.705
 dslf_fa13                    1.250       0.000       0.000
 omega                        0.400      90.726      36.290
 fa_dun                       0.700    1224.117     856.882
 p_aa_pp                      0.600     -65.076     -39.046
 yhh_planarity                0.625       0.000       0.000
 ref                          1.000     136.169     136.169
 rama_prepro                  0.450     -26.286     -11.829
---------------------------------------------------
 Total weighted score:                      230.682
```

### We need to relax the structure to optimize the conformations for point mutations.¶

In [9]:

```
movemap = MoveMap()
movemap.set_bb(False)
movemap.set_chi(True)
relax = pyrosetta.rosetta.protocols.relax.FastRelax()
relax.constrain_relax_to_start_coords(True)
relax.coord_constrain_sidechains(True)
relax.ramp_down_constraints(False)
relax.set_scorefxn(sfxn)
relax.set_movemap(movemap)
relax.apply(temp_pose)
temp_pose.dump_pdb('pdb/5T5K.relax.pdb')
```

```
protocols.relax.RelaxScriptManager: Reading relax scripts list from database.
core.scoring.ScoreFunctionFactory: SCOREFUNCTION: ref2015
protocols.relax.RelaxScriptManager: Looking for MonomerRelax2019.txt
protocols.relax.RelaxScriptManager: ================== Reading script file: /Users/breewenck/.local/lib/python3.7/site-packages/pyrosetta/database/sampling/relax_scripts/MonomerRelax2019.txt ==================
protocols.relax.RelaxScriptManager: repeat %%nrepeats%%
protocols.relax.RelaxScriptManager: coord_cst_weight 1.0
protocols.relax.RelaxScriptManager: scale:fa_rep 0.040
protocols.relax.RelaxScriptManager: repack
protocols.relax.RelaxScriptManager: scale:fa_rep 0.051
protocols.relax.RelaxScriptManager: min 0.01
protocols.relax.RelaxScriptManager: coord_cst_weight 0.5
protocols.relax.RelaxScriptManager: scale:fa_rep 0.265
protocols.relax.RelaxScriptManager: repack
protocols.relax.RelaxScriptManager: scale:fa_rep 0.280
protocols.relax.RelaxScriptManager: min 0.01
protocols.relax.RelaxScriptManager: coord_cst_weight 0.0
protocols.relax.RelaxScriptManager: scale:fa_rep 0.559
protocols.relax.RelaxScriptManager: repack
protocols.relax.RelaxScriptManager: scale:fa_rep 0.581
protocols.relax.RelaxScriptManager: min 0.01
protocols.relax.RelaxScriptManager: coord_cst_weight 0.0
protocols.relax.RelaxScriptManager: scale:fa_rep 1
protocols.relax.RelaxScriptManager: repack
protocols.relax.RelaxScriptManager: min 0.00001
protocols.relax.RelaxScriptManager: accept_to_best
protocols.relax.RelaxScriptManager: endrepeat
protocols.relax: turning off DNA bb and chi move
protocols.relax: turning off DNA bb and chi move
protocols.relax: turning off DNA bb and chi move
protocols.relax: turning off DNA bb and chi move
protocols.relax: turning off DNA bb and chi move
protocols.relax: turning off DNA bb and chi move
protocols.relax: turning off DNA bb and chi move
protocols.relax: turning off DNA bb and chi move
protocols.relax: turning off DNA bb and chi move
protocols.relax: turning off DNA bb and chi move
protocols.relax: turning off DNA bb and chi move
protocols.relax: turning off DNA bb and chi move
protocols.relax: turning off DNA bb and chi move
protocols.relax: turning off DNA bb and chi move
protocols.relax: turning off DNA bb and chi move
protocols.relax: turning off DNA bb and chi move
protocols.relax: turning off DNA bb and chi move
protocols.relax: turning off DNA bb and chi move
protocols.relax: turning off DNA bb and chi move
protocols.relax: turning off DNA bb and chi move
protocols.relax: turning off DNA bb and chi move
protocols.relax: turning off DNA bb and chi move
protocols.relax: turning off DNA bb and chi move
protocols.relax: turning off DNA bb and chi move
protocols.relax: turning off DNA bb and chi move
protocols.relax: turning off DNA bb and chi move
protocols.relax: turning off DNA bb and chi move
protocols.relax: turning off DNA bb and chi move
protocols.relax: turning off DNA bb and chi move
protocols.relax: turning off DNA bb and chi move
protocols.relax: turning off DNA bb and chi move
protocols.relax: turning off DNA bb and chi move
protocols.relax: turning off DNA bb and chi move
protocols.relax: turning off DNA bb and chi move
protocols.relax: turning off DNA bb and chi move
protocols.relax: turning off DNA bb and chi move
protocols.relax: turning off DNA bb and chi move
protocols.relax: turning off DNA bb and chi move
protocols.relax: turning off DNA bb and chi move
protocols.relax: turning off DNA bb and chi move
protocols.relax: turning off DNA bb and chi move
protocols.relax: turning off DNA bb and chi move
protocols.relax: turning off DNA bb and chi move
protocols.relax: turning off DNA bb and chi move
protocols.relax: turning off DNA bb and chi move
protocols.relax: turning off DNA bb and chi move
protocols.relax: turning off DNA bb and chi move
protocols.relax: turning off DNA bb and chi move
protocols.relax: turning off DNA bb and chi move
protocols.relax: turning off DNA bb and chi move
protocols.relax: turning off DNA bb and chi move
protocols.relax: turning off DNA bb and chi move
protocols.relax: turning off DNA bb and chi move
protocols.relax: turning off DNA bb and chi move
protocols.relax: turning off DNA bb and chi move
protocols.relax: turning off DNA bb and chi move
protocols.relax: turning off DNA bb and chi move
protocols.relax: turning off DNA bb and chi move
protocols.relax: turning off DNA bb and chi move
protocols.relax: turning off DNA bb and chi move
protocols.relax: turning off DNA bb and chi move
protocols.relax: turning off DNA bb and chi move
protocols.relax: turning off DNA bb and chi move
protocols.relax: turning off DNA bb and chi move
protocols.relax: turning off DNA bb and chi move
protocols.relax: turning off DNA bb and chi move
protocols.relax: turning off DNA bb and chi move
protocols.relax: turning off DNA bb and chi move
protocols.relax: turning off DNA bb and chi move
protocols.relax: turning off DNA bb and chi move
protocols.relax: turning off DNA bb and chi move
protocols.relax: turning off DNA bb and chi move
protocols.relax: turning off DNA bb and chi move
protocols.relax: turning off DNA bb and chi move
protocols.relax: turning off DNA bb and chi move
protocols.relax: turning off DNA bb and chi move
protocols.relax: turning off DNA bb and chi move
protocols.relax: turning off DNA bb and chi move
protocols.relax: turning off DNA bb and chi move
protocols.relax: turning off DNA bb and chi move
protocols.relax: turning off DNA bb and chi move
protocols.relax: turning off DNA bb and chi move
protocols.relax: turning off DNA bb and chi move
protocols.relax: turning off DNA bb and chi move
protocols.relax: turning off DNA bb and chi move
protocols.relax: turning off DNA bb and chi move
protocols.relax: turning off DNA bb and chi move
protocols.relax: turning off DNA bb and chi move
protocols.relax: turning off DNA bb and chi move
protocols.relax: turning off DNA bb and chi move
protocols.relax: turning off DNA bb and chi move
protocols.relax: turning off DNA bb and chi move
protocols.relax: turning off DNA bb and chi move
protocols.relax: turning off DNA bb and chi move
protocols.relax: turning off DNA bb and chi move
protocols.relax: turning off DNA bb and chi move
protocols.relax: turning off DNA bb and chi move
protocols.relax: turning off DNA bb and chi move
protocols.relax: turning off DNA bb and chi move
protocols.relax: turning off DNA bb and chi move
protocols.relax: turning off DNA bb and chi move
protocols.relax: turning off DNA bb and chi move
protocols.relax: turning off DNA bb and chi move
protocols.relax: turning off DNA bb and chi move
protocols.relax: turning off DNA bb and chi move
protocols.relax: turning off DNA bb and chi move
protocols.relax: turning off DNA bb and chi move
protocols.relax: turning off DNA bb and chi move
protocols.relax: turning off DNA bb and chi move
protocols.relax: turning off DNA bb and chi move
protocols.relax: turning off DNA bb and chi move
protocols.relax: turning off DNA bb and chi move
protocols.relax: turning off DNA bb and chi move
protocols.relax: turning off DNA bb and chi move
protocols.relax: turning off DNA bb and chi move
protocols.relax: turning off DNA bb and chi move
protocols.relax: turning off DNA bb and chi move
protocols.relax: turning off DNA bb and chi move
protocols.relax: turning off DNA bb and chi move
protocols.relax: turning off DNA bb and chi move
protocols.relax: turning off DNA bb and chi move
protocols.relax: turning off DNA bb and chi move
protocols.relax: turning off DNA bb and chi move
protocols.relax: turning off DNA bb and chi move
protocols.relax: turning off DNA bb and chi move
protocols.relax: turning off DNA bb and chi move
protocols.relax: turning off DNA bb and chi move
protocols.relax: turning off DNA bb and chi move
protocols.relax: turning off DNA bb and chi move
protocols.relax: turning off DNA bb and chi move
protocols.relax: turning off DNA bb and chi move
protocols.relax: turning off DNA bb and chi move
protocols.relax: turning off DNA bb and chi move
protocols.relax: turning off DNA bb and chi move
protocols.relax: turning off DNA bb and chi move
protocols.relax: turning off DNA bb and chi move
protocols.relax: turning off DNA bb and chi move
protocols.relax: turning off DNA bb and chi move
protocols.relax: turning off DNA bb and chi move
protocols.relax: turning off DNA bb and chi move
protocols.relax: turning off DNA bb and chi move
protocols.relax: turning off DNA bb and chi move
protocols.relax: turning off DNA bb and chi move
protocols.relax: turning off DNA bb and chi move
protocols.relax: turning off DNA bb and chi move
protocols.relax: turning off DNA bb and chi move
protocols.relax: turning off DNA bb and chi move
protocols.relax: turning off DNA bb and chi move
protocols.relax: turning off DNA bb and chi move
protocols.relax: turning off DNA bb and chi move
protocols.relax: turning off DNA bb and chi move
protocols.relax: turning off DNA bb and chi move
protocols.relax: turning off DNA bb and chi move
protocols.relax: turning off DNA bb and chi move
protocols.relax: turning off DNA bb and chi move
protocols.relax: turning off DNA bb and chi move
protocols.relax: turning off DNA bb and chi move
protocols.relax: turning off DNA bb and chi move
protocols.relax: turning off DNA bb and chi move
protocols.relax: turning off DNA bb and chi move
protocols.relax: turning off DNA bb and chi move
protocols.relax: turning off DNA bb and chi move
protocols.relax: turning off DNA bb and chi move
protocols.relax: turning off DNA bb and chi move
protocols.relax: turning off DNA bb and chi move
protocols.relax: turning off DNA bb and chi move
protocols.relax: turning off DNA bb and chi move
protocols.relax: turning off DNA bb and chi move
protocols.relax: turning off DNA bb and chi move
protocols.relax: turning off DNA bb and chi move
protocols.relax: turning off DNA bb and chi move
protocols.relax: turning off DNA bb and chi move
protocols.relax: turning off DNA bb and chi move
protocols.relax: turning off DNA bb and chi move
protocols.relax: turning off DNA bb and chi move
protocols.relax: turning off DNA bb and chi move
protocols.relax: turning off DNA bb and chi move
protocols.relax: turning off DNA bb and chi move
protocols.relax: turning off DNA bb and chi move
protocols.relax: turning off DNA bb and chi move
protocols.relax.FastRelax: CMD: repeat  230.682  0  0  0.55
protocols.relax.FastRelax: CMD: coord_cst_weight  230.682  0  0  0.55
protocols.relax.FastRelax: CMD: scale:fa_rep  -757.065  0  0  0.022
core.pack.task: Packer task: initialize from command line()
core.pack.pack_rotamers: built 10015 rotamers at 584 positions.
core.pack.interaction_graph.interaction_graph_factory: Instantiating DensePDInteractionGraph
protocols.relax.FastRelax: CMD: repack  -1399.68  0  0  0.022
protocols.relax.FastRelax: CMD: scale:fa_rep  -1378.71  0  0  0.02805
protocols.relax.FastRelax: CMD: min  -1378.77  0  0  0.02805
protocols.relax.FastRelax: CMD: coord_cst_weight  -1378.77  0  0  0.02805
protocols.relax.FastRelax: CMD: scale:fa_rep  -970.783  0  0  0.14575
core.pack.task: Packer task: initialize from command line()
core.pack.pack_rotamers: built 9046 rotamers at 584 positions.
core.pack.interaction_graph.interaction_graph_factory: Instantiating DensePDInteractionGraph
protocols.relax.FastRelax: CMD: repack  -1090.73  0  0  0.14575
protocols.relax.FastRelax: CMD: scale:fa_rep  -1073.06  0  0  0.154
protocols.relax.FastRelax: CMD: min  -1073.18  0  0  0.154
protocols.relax.FastRelax: CMD: coord_cst_weight  -1073.18  0  0  0.154
protocols.relax.FastRelax: CMD: scale:fa_rep  -744.691  0  0  0.30745
core.pack.task: Packer task: initialize from command line()
core.pack.pack_rotamers: built 8567 rotamers at 584 positions.
core.pack.interaction_graph.interaction_graph_factory: Instantiating DensePDInteractionGraph
protocols.relax.FastRelax: CMD: repack  -760.602  0  0  0.30745
protocols.relax.FastRelax: CMD: scale:fa_rep  -736.185  0  0  0.31955
protocols.relax.FastRelax: CMD: min  -805.781  0  0  0.31955
protocols.relax.FastRelax: CMD: coord_cst_weight  -805.781  0  0  0.31955
protocols.relax.FastRelax: CMD: scale:fa_rep  -393.312  0  0  0.55
core.pack.task: Packer task: initialize from command line()
core.pack.pack_rotamers: built 8242 rotamers at 584 positions.
core.pack.interaction_graph.interaction_graph_factory: Instantiating DensePDInteractionGraph
protocols.relax.FastRelax: CMD: repack  -394.416  0  0  0.55
protocols.relax.FastRelax: CMD: min  -459.457  0  0  0.55
protocols.relax.FastRelax: MRP: 0  -459.457  -459.457  0  0
protocols.relax.FastRelax: CMD: accept_to_best  -459.457  0  0  0.55
protocols.relax.FastRelax: CMD: endrepeat  -459.457  0  0  0.55
protocols.relax.FastRelax: CMD: coord_cst_weight  -459.457  0  0  0.55
protocols.relax.FastRelax: CMD: scale:fa_rep  -1337.24  0  0  0.022
core.pack.task: Packer task: initialize from command line()
core.pack.pack_rotamers: built 10123 rotamers at 584 positions.
core.pack.interaction_graph.interaction_graph_factory: Instantiating DensePDInteractionGraph
protocols.relax.FastRelax: CMD: repack  -1422.57  0  0  0.022
protocols.relax.FastRelax: CMD: scale:fa_rep  -1402.55  0  0  0.02805
protocols.relax.FastRelax: CMD: min  -1402.59  0  0  0.02805
protocols.relax.FastRelax: CMD: coord_cst_weight  -1402.59  0  0  0.02805
protocols.relax.FastRelax: CMD: scale:fa_rep  -1013.16  0  0  0.14575
core.pack.task: Packer task: initialize from command line()
core.pack.pack_rotamers: built 9046 rotamers at 584 positions.
core.pack.interaction_graph.interaction_graph_factory: Instantiating DensePDInteractionGraph
protocols.relax.FastRelax: CMD: repack  -1117.56  0  0  0.14575
protocols.relax.FastRelax: CMD: scale:fa_rep  -1099.91  0  0  0.154
protocols.relax.FastRelax: CMD: min  -1100.01  0  0  0.154
protocols.relax.FastRelax: CMD: coord_cst_weight  -1100.01  0  0  0.154
protocols.relax.FastRelax: CMD: scale:fa_rep  -771.692  0  0  0.30745
core.pack.task: Packer task: initialize from command line()
core.pack.pack_rotamers: built 8567 rotamers at 584 positions.
core.pack.interaction_graph.interaction_graph_factory: Instantiating DensePDInteractionGraph
protocols.relax.FastRelax: CMD: repack  -799.637  0  0  0.30745
protocols.relax.FastRelax: CMD: scale:fa_rep  -775.948  0  0  0.31955
protocols.relax.FastRelax: CMD: min  -830.009  0  0  0.31955
protocols.relax.FastRelax: CMD: coord_cst_weight  -830.009  0  0  0.31955
protocols.relax.FastRelax: CMD: scale:fa_rep  -425.235  0  0  0.55
core.pack.task: Packer task: initialize from command line()
core.pack.pack_rotamers: built 8242 rotamers at 584 positions.
core.pack.interaction_graph.interaction_graph_factory: Instantiating DensePDInteractionGraph
protocols.relax.FastRelax: CMD: repack  -424.806  0  0  0.55
protocols.relax.FastRelax: CMD: min  -460.441  0  0  0.55
protocols.relax.FastRelax: MRP: 1  -460.441  -460.441  0  0
protocols.relax.FastRelax: CMD: accept_to_best  -460.441  0  0  0.55
protocols.relax.FastRelax: CMD: endrepeat  -460.441  0  0  0.55
protocols.relax.FastRelax: CMD: coord_cst_weight  -460.441  0  0  0.55
protocols.relax.FastRelax: CMD: scale:fa_rep  -1339.9  0  0  0.022
core.pack.task: Packer task: initialize from command line()
core.pack.pack_rotamers: built 10123 rotamers at 584 positions.
core.pack.interaction_graph.interaction_graph_factory: Instantiating DensePDInteractionGraph
protocols.relax.FastRelax: CMD: repack  -1421.83  0  0  0.022
protocols.relax.FastRelax: CMD: scale:fa_rep  -1401.96  0  0  0.02805
protocols.relax.FastRelax: CMD: min  -1401.99  0  0  0.02805
protocols.relax.FastRelax: CMD: coord_cst_weight  -1401.99  0  0  0.02805
protocols.relax.FastRelax: CMD: scale:fa_rep  -1015.42  0  0  0.14575
core.pack.task: Packer task: initialize from command line()
core.pack.pack_rotamers: built 9046 rotamers at 584 positions.
core.pack.interaction_graph.interaction_graph_factory: Instantiating DensePDInteractionGraph
protocols.relax.FastRelax: CMD: repack  -1123.42  0  0  0.14575
protocols.relax.FastRelax: CMD: scale:fa_rep  -1106.4  0  0  0.154
protocols.relax.FastRelax: CMD: min  -1106.47  0  0  0.154
protocols.relax.FastRelax: CMD: coord_cst_weight  -1106.47  0  0  0.154
protocols.relax.FastRelax: CMD: scale:fa_rep  -790.038  0  0  0.30745
core.pack.task: Packer task: initialize from command line()
core.pack.pack_rotamers: built 8567 rotamers at 584 positions.
core.pack.interaction_graph.interaction_graph_factory: Instantiating DensePDInteractionGraph
protocols.relax.FastRelax: CMD: repack  -800.652  0  0  0.30745
protocols.relax.FastRelax: CMD: scale:fa_rep  -776.92  0  0  0.31955
protocols.relax.FastRelax: CMD: min  -830.125  0  0  0.31955
protocols.relax.FastRelax: CMD: coord_cst_weight  -830.125  0  0  0.31955
protocols.relax.FastRelax: CMD: scale:fa_rep  -423.718  0  0  0.55
core.pack.task: Packer task: initialize from command line()
core.pack.pack_rotamers: built 8242 rotamers at 584 positions.
core.pack.interaction_graph.interaction_graph_factory: Instantiating DensePDInteractionGraph
protocols.relax.FastRelax: CMD: repack  -425.977  0  0  0.55
protocols.relax.FastRelax: CMD: min  -461.587  0  0  0.55
protocols.relax.FastRelax: MRP: 2  -461.587  -461.587  0  0
protocols.relax.FastRelax: CMD: accept_to_best  -461.587  0  0  0.55
protocols.relax.FastRelax: CMD: endrepeat  -461.587  0  0  0.55
protocols.relax.FastRelax: CMD: coord_cst_weight  -461.587  0  0  0.55
protocols.relax.FastRelax: CMD: scale:fa_rep  -1341.61  0  0  0.022
core.pack.task: Packer task: initialize from command line()
core.pack.pack_rotamers: built 10123 rotamers at 584 positions.
core.pack.interaction_graph.interaction_graph_factory: Instantiating DensePDInteractionGraph
protocols.relax.FastRelax: CMD: repack  -1426.18  0  0  0.022
protocols.relax.FastRelax: CMD: scale:fa_rep  -1405.74  0  0  0.02805
protocols.relax.FastRelax: CMD: min  -1405.78  0  0  0.02805
protocols.relax.FastRelax: CMD: coord_cst_weight  -1405.78  0  0  0.02805
protocols.relax.FastRelax: CMD: scale:fa_rep  -1008.04  0  0  0.14575
core.pack.task: Packer task: initialize from command line()
core.pack.pack_rotamers: built 9046 rotamers at 584 positions.
core.pack.interaction_graph.interaction_graph_factory: Instantiating DensePDInteractionGraph
protocols.relax.FastRelax: CMD: repack  -1123.29  0  0  0.14575
protocols.relax.FastRelax: CMD: scale:fa_rep  -1106.11  0  0  0.154
protocols.relax.FastRelax: CMD: min  -1106.18  0  0  0.154
protocols.relax.FastRelax: CMD: coord_cst_weight  -1106.18  0  0  0.154
protocols.relax.FastRelax: CMD: scale:fa_rep  -786.669  0  0  0.30745
core.pack.task: Packer task: initialize from command line()
core.pack.pack_rotamers: built 8567 rotamers at 584 positions.
core.pack.interaction_graph.interaction_graph_factory: Instantiating DensePDInteractionGraph
protocols.relax.FastRelax: CMD: repack  -798.545  0  0  0.30745
protocols.relax.FastRelax: CMD: scale:fa_rep  -774.579  0  0  0.31955
protocols.relax.FastRelax: CMD: min  -828.622  0  0  0.31955
protocols.relax.FastRelax: CMD: coord_cst_weight  -828.622  0  0  0.31955
protocols.relax.FastRelax: CMD: scale:fa_rep  -418.962  0  0  0.55
core.pack.task: Packer task: initialize from command line()
core.pack.pack_rotamers: built 8242 rotamers at 584 positions.
core.pack.interaction_graph.interaction_graph_factory: Instantiating DensePDInteractionGraph
protocols.relax.FastRelax: CMD: repack  -419.517  0  0  0.55
protocols.relax.FastRelax: CMD: min  -465.673  0  0  0.55
protocols.relax.FastRelax: MRP: 3  -465.673  -465.673  0  0
protocols.relax.FastRelax: CMD: accept_to_best  -465.673  0  0  0.55
protocols.relax.FastRelax: CMD: endrepeat  -465.673  0  0  0.55
protocols.relax.FastRelax: CMD: coord_cst_weight  -465.673  0  0  0.55
protocols.relax.FastRelax: CMD: scale:fa_rep  -1344.31  0  0  0.022
core.pack.task: Packer task: initialize from command line()
core.pack.pack_rotamers: built 10123 rotamers at 584 positions.
core.pack.interaction_graph.interaction_graph_factory: Instantiating DensePDInteractionGraph
protocols.relax.FastRelax: CMD: repack  -1427.85  0  0  0.022
protocols.relax.FastRelax: CMD: scale:fa_rep  -1408.32  0  0  0.02805
protocols.relax.FastRelax: CMD: min  -1408.36  0  0  0.02805
protocols.relax.FastRelax: CMD: coord_cst_weight  -1408.36  0  0  0.02805
protocols.relax.FastRelax: CMD: scale:fa_rep  -1028.47  0  0  0.14575
core.pack.task: Packer task: initialize from command line()
core.pack.pack_rotamers: built 9046 rotamers at 584 positions.
core.pack.interaction_graph.interaction_graph_factory: Instantiating DensePDInteractionGraph
protocols.relax.FastRelax: CMD: repack  -1125.6  0  0  0.14575
protocols.relax.FastRelax: CMD: scale:fa_rep  -1108.44  0  0  0.154
protocols.relax.FastRelax: CMD: min  -1108.51  0  0  0.154
protocols.relax.FastRelax: CMD: coord_cst_weight  -1108.51  0  0  0.154
protocols.relax.FastRelax: CMD: scale:fa_rep  -789.401  0  0  0.30745
core.pack.task: Packer task: initialize from command line()
core.pack.pack_rotamers: built 8567 rotamers at 584 positions.
core.pack.interaction_graph.interaction_graph_factory: Instantiating DensePDInteractionGraph
protocols.relax.FastRelax: CMD: repack  -802.599  0  0  0.30745
protocols.relax.FastRelax: CMD: scale:fa_rep  -778.771  0  0  0.31955
protocols.relax.FastRelax: CMD: min  -831.211  0  0  0.31955
protocols.relax.FastRelax: CMD: coord_cst_weight  -831.211  0  0  0.31955
protocols.relax.FastRelax: CMD: scale:fa_rep  -422.561  0  0  0.55
core.pack.task: Packer task: initialize from command line()
core.pack.pack_rotamers: built 8242 rotamers at 584 positions.
core.pack.interaction_graph.interaction_graph_factory: Instantiating DensePDInteractionGraph
protocols.relax.FastRelax: CMD: repack  -418.688  0  0  0.55
protocols.relax.FastRelax: CMD: min  -461.43  0  0  0.55
protocols.relax.FastRelax: MRP: 4  -461.43  -465.673  0  0
protocols.relax.FastRelax: CMD: accept_to_best  -461.43  0  0  0.55
protocols.relax.FastRelax: CMD: endrepeat  -461.43  0  0  0.55
protocols::checkpoint: Deleting checkpoints of FastRelax
```

Out[9]:

```
True
```

In [10]:

```
rpose = pose_from_pdb('pdb/5T5K.relax.pdb')
sfxn.show(rpose)
```

```
core.import_pose.import_pose: File 'pdb/5T5K.relax.pdb' automatically determined to be of type PDB
core.scoring.ScoreFunction: 
------------------------------------------------------------
 Scores                       Weight   Raw Score Wghtd.Score
------------------------------------------------------------
 fa_atr                       1.000   -4744.189   -4744.189
 fa_rep                       0.550    1664.127     915.270
 fa_sol                       1.000    4233.850    4233.850
 fa_intra_rep                 0.005    2700.248      13.501
 fa_intra_sol_xover4          1.000     527.013     527.013
 lk_ball_wtd                  1.000    -272.862    -272.862
 fa_elec                      1.000   -1197.843   -1197.843
 pro_close                    1.250       1.118       1.397
 hbond_sr_bb                  1.000    -221.670    -221.670
 hbond_lr_bb                  1.000     -20.687     -20.687
 hbond_bb_sc                  1.000     -31.379     -31.379
 hbond_sc                     1.000    -305.888    -305.888
 dslf_fa13                    1.250       0.000       0.000
 omega                        0.400      90.726      36.290
 fa_dun                       0.700     737.378     516.165
 p_aa_pp                      0.600     -65.076     -39.046
 yhh_planarity                0.625       0.000       0.000
 ref                          1.000     136.169     136.169
 rama_prepro                  0.450     -26.286     -11.829
---------------------------------------------------
 Total weighted score:                     -465.737
```

### Observation of the scorefunction before and after relaxing the structure shows a significant reduction in the overall free energy, important for finding optimal conformations for point mutations.¶

In [11]:

```
from pyrosetta.toolbox import mutate_residue
```

In [12]:

```
R20S = pyrosetta.pose_from_pdb("pdb/5T5K.relax.pdb")
mutate_residue(R20S, 19, "S")
sfxn.show(R20S)
```

```
core.import_pose.import_pose: File 'pdb/5T5K.relax.pdb' automatically determined to be of type PDB
core.scoring.ScoreFunctionFactory: SCOREFUNCTION: ref2015
core.pack.task: Packer task: initialize from command line()
core.pack.pack_rotamers: built 162 rotamers at 1 positions.
core.pack.interaction_graph.interaction_graph_factory: Instantiating PDInteractionGraph
core.scoring.ScoreFunction: 
------------------------------------------------------------
 Scores                       Weight   Raw Score Wghtd.Score
------------------------------------------------------------
 fa_atr                       1.000   -4737.166   -4737.166
 fa_rep                       0.550    1662.443     914.344
 fa_sol                       1.000    4226.793    4226.793
 fa_intra_rep                 0.005    2696.823      13.484
 fa_intra_sol_xover4          1.000     526.656     526.656
 lk_ball_wtd                  1.000    -273.066    -273.066
 fa_elec                      1.000   -1196.448   -1196.448
 pro_close                    1.250       1.118       1.397
 hbond_sr_bb                  1.000    -221.670    -221.670
 hbond_lr_bb                  1.000     -20.687     -20.687
 hbond_bb_sc                  1.000     -31.379     -31.379
 hbond_sc                     1.000    -304.942    -304.942
 dslf_fa13                    1.250       0.000       0.000
 omega                        0.400      90.726      36.290
 fa_dun                       0.700     734.324     514.027
 p_aa_pp                      0.600     -64.657     -38.794
 yhh_planarity                0.625       0.000       0.000
 ref                          1.000     135.974     135.974
 rama_prepro                  0.450     -25.666     -11.550
---------------------------------------------------
 Total weighted score:                     -466.737
```

In [13]:

```
T55L = pyrosetta.pose_from_pdb("pdb/5T5K.relax.pdb")
mutate_residue(T55L, 54, "L")
sfxn.show(T55L)
T55L.dump_pdb("pdb/5T5K.T55L.pdb")
```

```
core.import_pose.import_pose: File 'pdb/5T5K.relax.pdb' automatically determined to be of type PDB
core.scoring.ScoreFunctionFactory: SCOREFUNCTION: ref2015
core.pack.task: Packer task: initialize from command line()
core.pack.pack_rotamers: built 1 rotamers at 1 positions.
core.pack.interaction_graph.interaction_graph_factory: Instantiating PDInteractionGraph
core.scoring.ScoreFunction: 
------------------------------------------------------------
 Scores                       Weight   Raw Score Wghtd.Score
------------------------------------------------------------
 fa_atr                       1.000   -4749.275   -4749.275
 fa_rep                       0.550    1933.516    1063.434
 fa_sol                       1.000    4233.632    4233.632
 fa_intra_rep                 0.005    2702.504      13.513
 fa_intra_sol_xover4          1.000     527.029     527.029
 lk_ball_wtd                  1.000    -273.077    -273.077
 fa_elec                      1.000   -1196.547   -1196.547
 pro_close                    1.250       1.118       1.397
 hbond_sr_bb                  1.000    -221.670    -221.670
 hbond_lr_bb                  1.000     -20.687     -20.687
 hbond_bb_sc                  1.000     -31.379     -31.379
 hbond_sc                     1.000    -305.888    -305.888
 dslf_fa13                    1.250       0.000       0.000
 omega                        0.400      90.726      36.290
 fa_dun                       0.700     740.045     518.032
 p_aa_pp                      0.600     -65.617     -39.370
 yhh_planarity                0.625       0.000       0.000
 ref                          1.000     136.679     136.679
 rama_prepro                  0.450     -26.864     -12.089
---------------------------------------------------
 Total weighted score:                     -319.977
```

Out[13]:

```
True
```

### Because the free energy associated with the T55L variant is significantly greater than WT (rpose), we need to relax the structure to eliminate any incompatible conformations.¶

In [14]:

```
temp_T55L = pyrosetta.pose_from_pdb("pdb/5T5K.T55L.pdb")
movemap = MoveMap()
movemap.set_bb(False)
movemap.set_chi(True)
relax = pyrosetta.rosetta.protocols.relax.FastRelax()
relax.constrain_relax_to_start_coords(True)
relax.coord_constrain_sidechains(True)
relax.ramp_down_constraints(False)
relax.set_scorefxn(sfxn)
relax.set_movemap(movemap)
relax.apply(temp_T55L)
temp_T55L.dump_pdb("pdb/5T5K.relax_T55L.pdb")
```

```
core.import_pose.import_pose: File 'pdb/5T5K.T55L.pdb' automatically determined to be of type PDB
core.scoring.ScoreFunctionFactory: SCOREFUNCTION: ref2015
protocols.relax: turning off DNA bb and chi move
protocols.relax: turning off DNA bb and chi move
protocols.relax: turning off DNA bb and chi move
protocols.relax: turning off DNA bb and chi move
protocols.relax: turning off DNA bb and chi move
protocols.relax: turning off DNA bb and chi move
protocols.relax: turning off DNA bb and chi move
protocols.relax: turning off DNA bb and chi move
protocols.relax: turning off DNA bb and chi move
protocols.relax: turning off DNA bb and chi move
protocols.relax: turning off DNA bb and chi move
protocols.relax: turning off DNA bb and chi move
protocols.relax: turning off DNA bb and chi move
protocols.relax: turning off DNA bb and chi move
protocols.relax: turning off DNA bb and chi move
protocols.relax: turning off DNA bb and chi move
protocols.relax: turning off DNA bb and chi move
protocols.relax: turning off DNA bb and chi move
protocols.relax: turning off DNA bb and chi move
protocols.relax: turning off DNA bb and chi move
protocols.relax: turning off DNA bb and chi move
protocols.relax: turning off DNA bb and chi move
protocols.relax: turning off DNA bb and chi move
protocols.relax: turning off DNA bb and chi move
protocols.relax: turning off DNA bb and chi move
protocols.relax: turning off DNA bb and chi move
protocols.relax: turning off DNA bb and chi move
protocols.relax: turning off DNA bb and chi move
protocols.relax: turning off DNA bb and chi move
protocols.relax: turning off DNA bb and chi move
protocols.relax: turning off DNA bb and chi move
protocols.relax: turning off DNA bb and chi move
protocols.relax: turning off DNA bb and chi move
protocols.relax: turning off DNA bb and chi move
protocols.relax: turning off DNA bb and chi move
protocols.relax: turning off DNA bb and chi move
protocols.relax: turning off DNA bb and chi move
protocols.relax: turning off DNA bb and chi move
protocols.relax: turning off DNA bb and chi move
protocols.relax: turning off DNA bb and chi move
protocols.relax: turning off DNA bb and chi move
protocols.relax: turning off DNA bb and chi move
protocols.relax: turning off DNA bb and chi move
protocols.relax: turning off DNA bb and chi move
protocols.relax: turning off DNA bb and chi move
protocols.relax: turning off DNA bb and chi move
protocols.relax: turning off DNA bb and chi move
protocols.relax: turning off DNA bb and chi move
protocols.relax: turning off DNA bb and chi move
protocols.relax: turning off DNA bb and chi move
protocols.relax: turning off DNA bb and chi move
protocols.relax: turning off DNA bb and chi move
protocols.relax: turning off DNA bb and chi move
protocols.relax: turning off DNA bb and chi move
protocols.relax: turning off DNA bb and chi move
protocols.relax: turning off DNA bb and chi move
protocols.relax: turning off DNA bb and chi move
protocols.relax: turning off DNA bb and chi move
protocols.relax: turning off DNA bb and chi move
protocols.relax: turning off DNA bb and chi move
protocols.relax: turning off DNA bb and chi move
protocols.relax: turning off DNA bb and chi move
protocols.relax: turning off DNA bb and chi move
protocols.relax: turning off DNA bb and chi move
protocols.relax: turning off DNA bb and chi move
protocols.relax: turning off DNA bb and chi move
protocols.relax: turning off DNA bb and chi move
protocols.relax: turning off DNA bb and chi move
protocols.relax: turning off DNA bb and chi move
protocols.relax: turning off DNA bb and chi move
protocols.relax: turning off DNA bb and chi move
protocols.relax: turning off DNA bb and chi move
protocols.relax: turning off DNA bb and chi move
protocols.relax: turning off DNA bb and chi move
protocols.relax: turning off DNA bb and chi move
protocols.relax: turning off DNA bb and chi move
protocols.relax: turning off DNA bb and chi move
protocols.relax: turning off DNA bb and chi move
protocols.relax: turning off DNA bb and chi move
protocols.relax: turning off DNA bb and chi move
protocols.relax: turning off DNA bb and chi move
protocols.relax: turning off DNA bb and chi move
protocols.relax: turning off DNA bb and chi move
protocols.relax: turning off DNA bb and chi move
protocols.relax: turning off DNA bb and chi move
protocols.relax: turning off DNA bb and chi move
protocols.relax: turning off DNA bb and chi move
protocols.relax: turning off DNA bb and chi move
protocols.relax: turning off DNA bb and chi move
protocols.relax: turning off DNA bb and chi move
protocols.relax: turning off DNA bb and chi move
protocols.relax: turning off DNA bb and chi move
protocols.relax: turning off DNA bb and chi move
protocols.relax: turning off DNA bb and chi move
protocols.relax: turning off DNA bb and chi move
protocols.relax: turning off DNA bb and chi move
protocols.relax: turning off DNA bb and chi move
protocols.relax: turning off DNA bb and chi move
protocols.relax: turning off DNA bb and chi move
protocols.relax: turning off DNA bb and chi move
protocols.relax: turning off DNA bb and chi move
protocols.relax: turning off DNA bb and chi move
protocols.relax: turning off DNA bb and chi move
protocols.relax: turning off DNA bb and chi move
protocols.relax: turning off DNA bb and chi move
protocols.relax: turning off DNA bb and chi move
protocols.relax: turning off DNA bb and chi move
protocols.relax: turning off DNA bb and chi move
protocols.relax: turning off DNA bb and chi move
protocols.relax: turning off DNA bb and chi move
protocols.relax: turning off DNA bb and chi move
protocols.relax: turning off DNA bb and chi move
protocols.relax: turning off DNA bb and chi move
protocols.relax: turning off DNA bb and chi move
protocols.relax: turning off DNA bb and chi move
protocols.relax: turning off DNA bb and chi move
protocols.relax: turning off DNA bb and chi move
protocols.relax: turning off DNA bb and chi move
protocols.relax: turning off DNA bb and chi move
protocols.relax: turning off DNA bb and chi move
protocols.relax: turning off DNA bb and chi move
protocols.relax: turning off DNA bb and chi move
protocols.relax: turning off DNA bb and chi move
protocols.relax: turning off DNA bb and chi move
protocols.relax: turning off DNA bb and chi move
protocols.relax: turning off DNA bb and chi move
protocols.relax: turning off DNA bb and chi move
protocols.relax: turning off DNA bb and chi move
protocols.relax: turning off DNA bb and chi move
protocols.relax: turning off DNA bb and chi move
protocols.relax: turning off DNA bb and chi move
protocols.relax: turning off DNA bb and chi move
protocols.relax: turning off DNA bb and chi move
protocols.relax: turning off DNA bb and chi move
protocols.relax: turning off DNA bb and chi move
protocols.relax: turning off DNA bb and chi move
protocols.relax: turning off DNA bb and chi move
protocols.relax: turning off DNA bb and chi move
protocols.relax: turning off DNA bb and chi move
protocols.relax: turning off DNA bb and chi move
protocols.relax: turning off DNA bb and chi move
protocols.relax: turning off DNA bb and chi move
protocols.relax: turning off DNA bb and chi move
protocols.relax: turning off DNA bb and chi move
protocols.relax: turning off DNA bb and chi move
protocols.relax: turning off DNA bb and chi move
protocols.relax: turning off DNA bb and chi move
protocols.relax: turning off DNA bb and chi move
protocols.relax: turning off DNA bb and chi move
protocols.relax: turning off DNA bb and chi move
protocols.relax: turning off DNA bb and chi move
protocols.relax: turning off DNA bb and chi move
protocols.relax: turning off DNA bb and chi move
protocols.relax: turning off DNA bb and chi move
protocols.relax: turning off DNA bb and chi move
protocols.relax: turning off DNA bb and chi move
protocols.relax: turning off DNA bb and chi move
protocols.relax: turning off DNA bb and chi move
protocols.relax: turning off DNA bb and chi move
protocols.relax: turning off DNA bb and chi move
protocols.relax: turning off DNA bb and chi move
protocols.relax: turning off DNA bb and chi move
protocols.relax: turning off DNA bb and chi move
protocols.relax: turning off DNA bb and chi move
protocols.relax: turning off DNA bb and chi move
protocols.relax: turning off DNA bb and chi move
protocols.relax: turning off DNA bb and chi move
protocols.relax: turning off DNA bb and chi move
protocols.relax: turning off DNA bb and chi move
protocols.relax: turning off DNA bb and chi move
protocols.relax: turning off DNA bb and chi move
protocols.relax: turning off DNA bb and chi move
protocols.relax: turning off DNA bb and chi move
protocols.relax: turning off DNA bb and chi move
protocols.relax: turning off DNA bb and chi move
protocols.relax: turning off DNA bb and chi move
protocols.relax: turning off DNA bb and chi move
protocols.relax: turning off DNA bb and chi move
protocols.relax: turning off DNA bb and chi move
protocols.relax: turning off DNA bb and chi move
protocols.relax.FastRelax: CMD: repeat  -319.973  0  0  0.55
protocols.relax.FastRelax: CMD: coord_cst_weight  -319.973  0  0  0.55
protocols.relax.FastRelax: CMD: scale:fa_rep  -1340.87  0  0  0.022
core.pack.task: Packer task: initialize from command line()
core.pack.pack_rotamers: built 10090 rotamers at 584 positions.
core.pack.interaction_graph.interaction_graph_factory: Instantiating DensePDInteractionGraph
protocols.relax.FastRelax: CMD: repack  -1426.22  0  0  0.022
protocols.relax.FastRelax: CMD: scale:fa_rep  -1404.84  0  0  0.02805
protocols.relax.FastRelax: CMD: min  -1404.88  0  0  0.02805
protocols.relax.FastRelax: CMD: coord_cst_weight  -1404.88  0  0  0.02805
protocols.relax.FastRelax: CMD: scale:fa_rep  -988.94  0  0  0.14575
core.pack.task: Packer task: initialize from command line()
core.pack.pack_rotamers: built 9012 rotamers at 584 positions.
core.pack.interaction_graph.interaction_graph_factory: Instantiating DensePDInteractionGraph
protocols.relax.FastRelax: CMD: repack  -1101.51  0  0  0.14575
protocols.relax.FastRelax: CMD: scale:fa_rep  -1083.31  0  0  0.154
protocols.relax.FastRelax: CMD: min  -1083.4  0  0  0.154
protocols.relax.FastRelax: CMD: coord_cst_weight  -1083.4  0  0  0.154
protocols.relax.FastRelax: CMD: scale:fa_rep  -744.984  0  0  0.30745
core.pack.task: Packer task: initialize from command line()
core.pack.pack_rotamers: built 8533 rotamers at 584 positions.
core.pack.interaction_graph.interaction_graph_factory: Instantiating DensePDInteractionGraph
protocols.relax.FastRelax: CMD: repack  -758.538  0  0  0.30745
protocols.relax.FastRelax: CMD: scale:fa_rep  -733.314  0  0  0.31955
protocols.relax.FastRelax: CMD: min  -803.649  0  0  0.31955
protocols.relax.FastRelax: CMD: coord_cst_weight  -803.649  0  0  0.31955
protocols.relax.FastRelax: CMD: scale:fa_rep  -383.055  0  0  0.55
core.pack.task: Packer task: initialize from command line()
core.pack.pack_rotamers: built 8207 rotamers at 584 positions.
core.pack.interaction_graph.interaction_graph_factory: Instantiating DensePDInteractionGraph
protocols.relax.FastRelax: CMD: repack  -382.659  0  0  0.55
protocols.relax.FastRelax: CMD: min  -425.781  0  0  0.55
protocols.relax.FastRelax: MRP: 0  -425.781  -425.781  0  0
protocols.relax.FastRelax: CMD: accept_to_best  -425.781  0  0  0.55
protocols.relax.FastRelax: CMD: endrepeat  -425.781  0  0  0.55
protocols.relax.FastRelax: CMD: coord_cst_weight  -425.781  0  0  0.55
protocols.relax.FastRelax: CMD: scale:fa_rep  -1337.35  0  0  0.022
core.pack.task: Packer task: initialize from command line()
core.pack.pack_rotamers: built 10090 rotamers at 584 positions.
core.pack.interaction_graph.interaction_graph_factory: Instantiating DensePDInteractionGraph
protocols.relax.FastRelax: CMD: repack  -1423.3  0  0  0.022
protocols.relax.FastRelax: CMD: scale:fa_rep  -1401.6  0  0  0.02805
protocols.relax.FastRelax: CMD: min  -1401.64  0  0  0.02805
protocols.relax.FastRelax: CMD: coord_cst_weight  -1401.64  0  0  0.02805
protocols.relax.FastRelax: CMD: scale:fa_rep  -979.648  0  0  0.14575
core.pack.task: Packer task: initialize from command line()
core.pack.pack_rotamers: built 9012 rotamers at 584 positions.
core.pack.interaction_graph.interaction_graph_factory: Instantiating DensePDInteractionGraph
protocols.relax.FastRelax: CMD: repack  -1103.18  0  0  0.14575
protocols.relax.FastRelax: CMD: scale:fa_rep  -1084.96  0  0  0.154
protocols.relax.FastRelax: CMD: min  -1085.05  0  0  0.154
protocols.relax.FastRelax: CMD: coord_cst_weight  -1085.05  0  0  0.154
protocols.relax.FastRelax: CMD: scale:fa_rep  -746.294  0  0  0.30745
core.pack.task: Packer task: initialize from command line()
core.pack.pack_rotamers: built 8533 rotamers at 584 positions.
core.pack.interaction_graph.interaction_graph_factory: Instantiating DensePDInteractionGraph
protocols.relax.FastRelax: CMD: repack  -758.098  0  0  0.30745
protocols.relax.FastRelax: CMD: scale:fa_rep  -733.076  0  0  0.31955
protocols.relax.FastRelax: CMD: min  -798.644  0  0  0.31955
protocols.relax.FastRelax: CMD: coord_cst_weight  -798.644  0  0  0.31955
protocols.relax.FastRelax: CMD: scale:fa_rep  -377.196  0  0  0.55
core.pack.task: Packer task: initialize from command line()
core.pack.pack_rotamers: built 8207 rotamers at 584 positions.
core.pack.interaction_graph.interaction_graph_factory: Instantiating DensePDInteractionGraph
protocols.relax.FastRelax: CMD: repack  -378.074  0  0  0.55
protocols.relax.FastRelax: CMD: min  -424.812  0  0  0.55
protocols.relax.FastRelax: MRP: 1  -424.812  -425.781  0  0
protocols.relax.FastRelax: CMD: accept_to_best  -424.812  0  0  0.55
protocols.relax.FastRelax: CMD: endrepeat  -424.812  0  0  0.55
protocols.relax.FastRelax: CMD: coord_cst_weight  -424.812  0  0  0.55
protocols.relax.FastRelax: CMD: scale:fa_rep  -1333.67  0  0  0.022
core.pack.task: Packer task: initialize from command line()
core.pack.pack_rotamers: built 10090 rotamers at 584 positions.
core.pack.interaction_graph.interaction_graph_factory: Instantiating DensePDInteractionGraph
protocols.relax.FastRelax: CMD: repack  -1425.15  0  0  0.022
protocols.relax.FastRelax: CMD: scale:fa_rep  -1403.16  0  0  0.02805
protocols.relax.FastRelax: CMD: min  -1403.2  0  0  0.02805
protocols.relax.FastRelax: CMD: coord_cst_weight  -1403.2  0  0  0.02805
protocols.relax.FastRelax: CMD: scale:fa_rep  -975.489  0  0  0.14575
core.pack.task: Packer task: initialize from command line()
core.pack.pack_rotamers: built 9012 rotamers at 584 positions.
core.pack.interaction_graph.interaction_graph_factory: Instantiating DensePDInteractionGraph
protocols.relax.FastRelax: CMD: repack  -1106.22  0  0  0.14575
protocols.relax.FastRelax: CMD: scale:fa_rep  -1088.02  0  0  0.154
protocols.relax.FastRelax: CMD: min  -1088.09  0  0  0.154
protocols.relax.FastRelax: CMD: coord_cst_weight  -1088.09  0  0  0.154
protocols.relax.FastRelax: CMD: scale:fa_rep  -749.621  0  0  0.30745
core.pack.task: Packer task: initialize from command line()
core.pack.pack_rotamers: built 8533 rotamers at 584 positions.
core.pack.interaction_graph.interaction_graph_factory: Instantiating DensePDInteractionGraph
protocols.relax.FastRelax: CMD: repack  -763.259  0  0  0.30745
protocols.relax.FastRelax: CMD: scale:fa_rep  -737.934  0  0  0.31955
protocols.relax.FastRelax: CMD: min  -804.815  0  0  0.31955
protocols.relax.FastRelax: CMD: coord_cst_weight  -804.815  0  0  0.31955
protocols.relax.FastRelax: CMD: scale:fa_rep  -380.536  0  0  0.55
core.pack.task: Packer task: initialize from command line()
core.pack.pack_rotamers: built 8207 rotamers at 584 positions.
core.pack.interaction_graph.interaction_graph_factory: Instantiating DensePDInteractionGraph
protocols.relax.FastRelax: CMD: repack  -377.019  0  0  0.55
protocols.relax.FastRelax: CMD: min  -427.694  0  0  0.55
protocols.relax.FastRelax: MRP: 2  -427.694  -427.694  0  0
protocols.relax.FastRelax: CMD: accept_to_best  -427.694  0  0  0.55
protocols.relax.FastRelax: CMD: endrepeat  -427.694  0  0  0.55
protocols.relax.FastRelax: CMD: coord_cst_weight  -427.694  0  0  0.55
protocols.relax.FastRelax: CMD: scale:fa_rep  -1331.84  0  0  0.022
core.pack.task: Packer task: initialize from command line()
core.pack.pack_rotamers: built 10090 rotamers at 584 positions.
core.pack.interaction_graph.interaction_graph_factory: Instantiating DensePDInteractionGraph
protocols.relax.FastRelax: CMD: repack  -1421.71  0  0  0.022
protocols.relax.FastRelax: CMD: scale:fa_rep  -1400.41  0  0  0.02805
protocols.relax.FastRelax: CMD: min  -1400.45  0  0  0.02805
protocols.relax.FastRelax: CMD: coord_cst_weight  -1400.45  0  0  0.02805
protocols.relax.FastRelax: CMD: scale:fa_rep  -986.063  0  0  0.14575
core.pack.task: Packer task: initialize from command line()
core.pack.pack_rotamers: built 9012 rotamers at 584 positions.
core.pack.interaction_graph.interaction_graph_factory: Instantiating DensePDInteractionGraph
protocols.relax.FastRelax: CMD: repack  -1104.02  0  0  0.14575
protocols.relax.FastRelax: CMD: scale:fa_rep  -1085.84  0  0  0.154
protocols.relax.FastRelax: CMD: min  -1085.91  0  0  0.154
protocols.relax.FastRelax: CMD: coord_cst_weight  -1085.91  0  0  0.154
protocols.relax.FastRelax: CMD: scale:fa_rep  -747.812  0  0  0.30745
core.pack.task: Packer task: initialize from command line()
core.pack.pack_rotamers: built 8533 rotamers at 584 positions.
core.pack.interaction_graph.interaction_graph_factory: Instantiating DensePDInteractionGraph
protocols.relax.FastRelax: CMD: repack  -758.598  0  0  0.30745
protocols.relax.FastRelax: CMD: scale:fa_rep  -733.331  0  0  0.31955
protocols.relax.FastRelax: CMD: min  -803.866  0  0  0.31955
protocols.relax.FastRelax: CMD: coord_cst_weight  -803.866  0  0  0.31955
protocols.relax.FastRelax: CMD: scale:fa_rep  -383.499  0  0  0.55
core.pack.task: Packer task: initialize from command line()
core.pack.pack_rotamers: built 8207 rotamers at 584 positions.
core.pack.interaction_graph.interaction_graph_factory: Instantiating DensePDInteractionGraph
protocols.relax.FastRelax: CMD: repack  -383.731  0  0  0.55
protocols.relax.FastRelax: CMD: min  -425.462  0  0  0.55
protocols.relax.FastRelax: MRP: 3  -425.462  -427.694  0  0
protocols.relax.FastRelax: CMD: accept_to_best  -425.462  0  0  0.55
protocols.relax.FastRelax: CMD: endrepeat  -425.462  0  0  0.55
protocols.relax.FastRelax: CMD: coord_cst_weight  -425.462  0  0  0.55
protocols.relax.FastRelax: CMD: scale:fa_rep  -1336.28  0  0  0.022
core.pack.task: Packer task: initialize from command line()
core.pack.pack_rotamers: built 10090 rotamers at 584 positions.
core.pack.interaction_graph.interaction_graph_factory: Instantiating DensePDInteractionGraph
protocols.relax.FastRelax: CMD: repack  -1426.3  0  0  0.022
protocols.relax.FastRelax: CMD: scale:fa_rep  -1404.5  0  0  0.02805
protocols.relax.FastRelax: CMD: min  -1404.54  0  0  0.02805
protocols.relax.FastRelax: CMD: coord_cst_weight  -1404.54  0  0  0.02805
protocols.relax.FastRelax: CMD: scale:fa_rep  -980.422  0  0  0.14575
core.pack.task: Packer task: initialize from command line()
core.pack.pack_rotamers: built 9012 rotamers at 584 positions.
core.pack.interaction_graph.interaction_graph_factory: Instantiating DensePDInteractionGraph
protocols.relax.FastRelax: CMD: repack  -1105.15  0  0  0.14575
protocols.relax.FastRelax: CMD: scale:fa_rep  -1086.96  0  0  0.154
protocols.relax.FastRelax: CMD: min  -1087.04  0  0  0.154
protocols.relax.FastRelax: CMD: coord_cst_weight  -1087.04  0  0  0.154
protocols.relax.FastRelax: CMD: scale:fa_rep  -748.748  0  0  0.30745
core.pack.task: Packer task: initialize from command line()
core.pack.pack_rotamers: built 8533 rotamers at 584 positions.
core.pack.interaction_graph.interaction_graph_factory: Instantiating DensePDInteractionGraph
protocols.relax.FastRelax: CMD: repack  -760.713  0  0  0.30745
protocols.relax.FastRelax: CMD: scale:fa_rep  -735.186  0  0  0.31955
protocols.relax.FastRelax: CMD: min  -803.225  0  0  0.31955
protocols.relax.FastRelax: CMD: coord_cst_weight  -803.225  0  0  0.31955
protocols.relax.FastRelax: CMD: scale:fa_rep  -375.125  0  0  0.55
core.pack.task: Packer task: initialize from command line()
core.pack.pack_rotamers: built 8207 rotamers at 584 positions.
core.pack.interaction_graph.interaction_graph_factory: Instantiating DensePDInteractionGraph
protocols.relax.FastRelax: CMD: repack  -372.411  0  0  0.55
protocols.relax.FastRelax: CMD: min  -424.25  0  0  0.55
protocols.relax.FastRelax: MRP: 4  -424.25  -427.694  0  0
protocols.relax.FastRelax: CMD: accept_to_best  -424.25  0  0  0.55
protocols.relax.FastRelax: CMD: endrepeat  -424.25  0  0  0.55
protocols::checkpoint: Deleting checkpoints of FastRelax
```

Out[14]:

```
True
```

In [15]:

```
rT55L = pyrosetta.pose_from_pdb("pdb/5T5K.relax_T55L.pdb")
sfxn.show(rT55L)
```

```
core.import_pose.import_pose: File 'pdb/5T5K.relax_T55L.pdb' automatically determined to be of type PDB
core.scoring.ScoreFunction: 
------------------------------------------------------------
 Scores                       Weight   Raw Score Wghtd.Score
------------------------------------------------------------
 fa_atr                       1.000   -4736.574   -4736.574
 fa_rep                       0.550    1712.493     941.871
 fa_sol                       1.000    4223.865    4223.865
 fa_intra_rep                 0.005    2732.588      13.663
 fa_intra_sol_xover4          1.000     526.218     526.218
 lk_ball_wtd                  1.000    -275.078    -275.078
 fa_elec                      1.000   -1189.044   -1189.044
 pro_close                    1.250       1.118       1.397
 hbond_sr_bb                  1.000    -221.670    -221.670
 hbond_lr_bb                  1.000     -20.687     -20.687
 hbond_bb_sc                  1.000     -31.262     -31.262
 hbond_sc                     1.000    -300.892    -300.892
 dslf_fa13                    1.250       0.000       0.000
 omega                        0.400      90.726      36.290
 fa_dun                       0.700     741.368     518.958
 p_aa_pp                      0.600     -65.617     -39.370
 yhh_planarity                0.625       0.000       0.000
 ref                          1.000     136.679     136.679
 rama_prepro                  0.450     -26.864     -12.089
---------------------------------------------------
 Total weighted score:                     -427.724
```

In [16]:

```
G17D = pose_from_pdb('pdb/5T5K.relax.pdb')
mutate_residue(G17D, 16, "D")
sfxn.show(G17D)
```

```
core.import_pose.import_pose: File 'pdb/5T5K.relax.pdb' automatically determined to be of type PDB
core.scoring.ScoreFunctionFactory: SCOREFUNCTION: ref2015
core.pack.task: Packer task: initialize from command line()
core.pack.pack_rotamers: built 1 rotamers at 1 positions.
core.pack.interaction_graph.interaction_graph_factory: Instantiating PDInteractionGraph
core.scoring.ScoreFunction: 
------------------------------------------------------------
 Scores                       Weight   Raw Score Wghtd.Score
------------------------------------------------------------
 fa_atr                       1.000   -4746.948   -4746.948
 fa_rep                       0.550    1711.788     941.484
 fa_sol                       1.000    4237.761    4237.761
 fa_intra_rep                 0.005    2701.674      13.508
 fa_intra_sol_xover4          1.000     527.382     527.382
 lk_ball_wtd                  1.000    -273.185    -273.185
 fa_elec                      1.000   -1197.025   -1197.025
 pro_close                    1.250       1.118       1.397
 hbond_sr_bb                  1.000    -221.670    -221.670
 hbond_lr_bb                  1.000     -20.687     -20.687
 hbond_bb_sc                  1.000     -31.379     -31.379
 hbond_sc                     1.000    -305.888    -305.888
 dslf_fa13                    1.250       0.000       0.000
 omega                        0.400      90.755      36.302
 fa_dun                       0.700     741.129     518.790
 p_aa_pp                      0.600     -59.222     -35.533
 yhh_planarity                0.625       0.000       0.000
 ref                          1.000     133.225     133.225
 rama_prepro                  0.450     -11.219      -5.049
---------------------------------------------------
 Total weighted score:                     -427.515
```

In [17]:

```
G17D.dump_pdb("pdb/5T5K.G17D.pdb")
```

Out[17]:

```
True
```

In [18]:

```
temp_G17D = pyrosetta.pose_from_pdb("pdb/5T5K.G17D.pdb")
movemap = MoveMap()
movemap.set_bb(False)
movemap.set_chi(True)
relax = pyrosetta.rosetta.protocols.relax.FastRelax()
relax.constrain_relax_to_start_coords(True)
relax.coord_constrain_sidechains(True)
relax.ramp_down_constraints(False)
relax.set_scorefxn(sfxn)
relax.set_movemap(movemap)
relax.apply(temp_G17D)
temp_G17D.dump_pdb("pdb/5T5K.relax_G17D.pdb")
```

```
core.import_pose.import_pose: File 'pdb/5T5K.G17D.pdb' automatically determined to be of type PDB
core.scoring.ScoreFunctionFactory: SCOREFUNCTION: ref2015
protocols.relax: turning off DNA bb and chi move
protocols.relax: turning off DNA bb and chi move
protocols.relax: turning off DNA bb and chi move
protocols.relax: turning off DNA bb and chi move
protocols.relax: turning off DNA bb and chi move
protocols.relax: turning off DNA bb and chi move
protocols.relax: turning off DNA bb and chi move
protocols.relax: turning off DNA bb and chi move
protocols.relax: turning off DNA bb and chi move
protocols.relax: turning off DNA bb and chi move
protocols.relax: turning off DNA bb and chi move
protocols.relax: turning off DNA bb and chi move
protocols.relax: turning off DNA bb and chi move
protocols.relax: turning off DNA bb and chi move
protocols.relax: turning off DNA bb and chi move
protocols.relax: turning off DNA bb and chi move
protocols.relax: turning off DNA bb and chi move
protocols.relax: turning off DNA bb and chi move
protocols.relax: turning off DNA bb and chi move
protocols.relax: turning off DNA bb and chi move
protocols.relax: turning off DNA bb and chi move
protocols.relax: turning off DNA bb and chi move
protocols.relax: turning off DNA bb and chi move
protocols.relax: turning off DNA bb and chi move
protocols.relax: turning off DNA bb and chi move
protocols.relax: turning off DNA bb and chi move
protocols.relax: turning off DNA bb and chi move
protocols.relax: turning off DNA bb and chi move
protocols.relax: turning off DNA bb and chi move
protocols.relax: turning off DNA bb and chi move
protocols.relax: turning off DNA bb and chi move
protocols.relax: turning off DNA bb and chi move
protocols.relax: turning off DNA bb and chi move
protocols.relax: turning off DNA bb and chi move
protocols.relax: turning off DNA bb and chi move
protocols.relax: turning off DNA bb and chi move
protocols.relax: turning off DNA bb and chi move
protocols.relax: turning off DNA bb and chi move
protocols.relax: turning off DNA bb and chi move
protocols.relax: turning off DNA bb and chi move
protocols.relax: turning off DNA bb and chi move
protocols.relax: turning off DNA bb and chi move
protocols.relax: turning off DNA bb and chi move
protocols.relax: turning off DNA bb and chi move
protocols.relax: turning off DNA bb and chi move
protocols.relax: turning off DNA bb and chi move
protocols.relax: turning off DNA bb and chi move
protocols.relax: turning off DNA bb and chi move
protocols.relax: turning off DNA bb and chi move
protocols.relax: turning off DNA bb and chi move
protocols.relax: turning off DNA bb and chi move
protocols.relax: turning off DNA bb and chi move
protocols.relax: turning off DNA bb and chi move
protocols.relax: turning off DNA bb and chi move
protocols.relax: turning off DNA bb and chi move
protocols.relax: turning off DNA bb and chi move
protocols.relax: turning off DNA bb and chi move
protocols.relax: turning off DNA bb and chi move
protocols.relax: turning off DNA bb and chi move
protocols.relax: turning off DNA bb and chi move
protocols.relax: turning off DNA bb and chi move
protocols.relax: turning off DNA bb and chi move
protocols.relax: turning off DNA bb and chi move
protocols.relax: turning off DNA bb and chi move
protocols.relax: turning off DNA bb and chi move
protocols.relax: turning off DNA bb and chi move
protocols.relax: turning off DNA bb and chi move
protocols.relax: turning off DNA bb and chi move
protocols.relax: turning off DNA bb and chi move
protocols.relax: turning off DNA bb and chi move
protocols.relax: turning off DNA bb and chi move
protocols.relax: turning off DNA bb and chi move
protocols.relax: turning off DNA bb and chi move
protocols.relax: turning off DNA bb and chi move
protocols.relax: turning off DNA bb and chi move
protocols.relax: turning off DNA bb and chi move
protocols.relax: turning off DNA bb and chi move
protocols.relax: turning off DNA bb and chi move
protocols.relax: turning off DNA bb and chi move
protocols.relax: turning off DNA bb and chi move
protocols.relax: turning off DNA bb and chi move
protocols.relax: turning off DNA bb and chi move
protocols.relax: turning off DNA bb and chi move
protocols.relax: turning off DNA bb and chi move
protocols.relax: turning off DNA bb and chi move
protocols.relax: turning off DNA bb and chi move
protocols.relax: turning off DNA bb and chi move
protocols.relax: turning off DNA bb and chi move
protocols.relax: turning off DNA bb and chi move
protocols.relax: turning off DNA bb and chi move
protocols.relax: turning off DNA bb and chi move
protocols.relax: turning off DNA bb and chi move
protocols.relax: turning off DNA bb and chi move
protocols.relax: turning off DNA bb and chi move
protocols.relax: turning off DNA bb and chi move
protocols.relax: turning off DNA bb and chi move
protocols.relax: turning off DNA bb and chi move
protocols.relax: turning off DNA bb and chi move
protocols.relax: turning off DNA bb and chi move
protocols.relax: turning off DNA bb and chi move
protocols.relax: turning off DNA bb and chi move
protocols.relax: turning off DNA bb and chi move
protocols.relax: turning off DNA bb and chi move
protocols.relax: turning off DNA bb and chi move
protocols.relax: turning off DNA bb and chi move
protocols.relax: turning off DNA bb and chi move
protocols.relax: turning off DNA bb and chi move
protocols.relax: turning off DNA bb and chi move
protocols.relax: turning off DNA bb and chi move
protocols.relax: turning off DNA bb and chi move
protocols.relax: turning off DNA bb and chi move
protocols.relax: turning off DNA bb and chi move
protocols.relax: turning off DNA bb and chi move
protocols.relax: turning off DNA bb and chi move
protocols.relax: turning off DNA bb and chi move
protocols.relax: turning off DNA bb and chi move
protocols.relax: turning off DNA bb and chi move
protocols.relax: turning off DNA bb and chi move
protocols.relax: turning off DNA bb and chi move
protocols.relax: turning off DNA bb and chi move
protocols.relax: turning off DNA bb and chi move
protocols.relax: turning off DNA bb and chi move
protocols.relax: turning off DNA bb and chi move
protocols.relax: turning off DNA bb and chi move
protocols.relax: turning off DNA bb and chi move
protocols.relax: turning off DNA bb and chi move
protocols.relax: turning off DNA bb and chi move
protocols.relax: turning off DNA bb and chi move
protocols.relax: turning off DNA bb and chi move
protocols.relax: turning off DNA bb and chi move
protocols.relax: turning off DNA bb and chi move
protocols.relax: turning off DNA bb and chi move
protocols.relax: turning off DNA bb and chi move
protocols.relax: turning off DNA bb and chi move
protocols.relax: turning off DNA bb and chi move
protocols.relax: turning off DNA bb and chi move
protocols.relax: turning off DNA bb and chi move
protocols.relax: turning off DNA bb and chi move
protocols.relax: turning off DNA bb and chi move
protocols.relax: turning off DNA bb and chi move
protocols.relax: turning off DNA bb and chi move
protocols.relax: turning off DNA bb and chi move
protocols.relax: turning off DNA bb and chi move
protocols.relax: turning off DNA bb and chi move
protocols.relax: turning off DNA bb and chi move
protocols.relax: turning off DNA bb and chi move
protocols.relax: turning off DNA bb and chi move
protocols.relax: turning off DNA bb and chi move
protocols.relax: turning off DNA bb and chi move
protocols.relax: turning off DNA bb and chi move
protocols.relax: turning off DNA bb and chi move
protocols.relax: turning off DNA bb and chi move
protocols.relax: turning off DNA bb and chi move
protocols.relax: turning off DNA bb and chi move
protocols.relax: turning off DNA bb and chi move
protocols.relax: turning off DNA bb and chi move
protocols.relax: turning off DNA bb and chi move
protocols.relax: turning off DNA bb and chi move
protocols.relax: turning off DNA bb and chi move
protocols.relax: turning off DNA bb and chi move
protocols.relax: turning off DNA bb and chi move
protocols.relax: turning off DNA bb and chi move
protocols.relax: turning off DNA bb and chi move
protocols.relax: turning off DNA bb and chi move
protocols.relax: turning off DNA bb and chi move
protocols.relax: turning off DNA bb and chi move
protocols.relax: turning off DNA bb and chi move
protocols.relax: turning off DNA bb and chi move
protocols.relax: turning off DNA bb and chi move
protocols.relax: turning off DNA bb and chi move
protocols.relax: turning off DNA bb and chi move
protocols.relax: turning off DNA bb and chi move
protocols.relax: turning off DNA bb and chi move
protocols.relax: turning off DNA bb and chi move
protocols.relax: turning off DNA bb and chi move
protocols.relax: turning off DNA bb and chi move
protocols.relax: turning off DNA bb and chi move
protocols.relax: turning off DNA bb and chi move
protocols.relax: turning off DNA bb and chi move
protocols.relax: turning off DNA bb and chi move
protocols.relax.FastRelax: CMD: repeat  -427.523  0  0  0.55
protocols.relax.FastRelax: CMD: coord_cst_weight  -427.523  0  0  0.55
protocols.relax.FastRelax: CMD: scale:fa_rep  -1331.34  0  0  0.022
core.pack.task: Packer task: initialize from command line()
core.pack.pack_rotamers: built 10129 rotamers at 584 positions.
core.pack.interaction_graph.interaction_graph_factory: Instantiating DensePDInteractionGraph
protocols.relax.FastRelax: CMD: repack  -1413.41  0  0  0.022
protocols.relax.FastRelax: CMD: scale:fa_rep  -1393.26  0  0  0.02805
protocols.relax.FastRelax: CMD: min  -1393.3  0  0  0.02805
protocols.relax.FastRelax: CMD: coord_cst_weight  -1393.3  0  0  0.02805
protocols.relax.FastRelax: CMD: scale:fa_rep  -1001.29  0  0  0.14575
core.pack.task: Packer task: initialize from command line()
core.pack.pack_rotamers: built 9049 rotamers at 584 positions.
core.pack.interaction_graph.interaction_graph_factory: Instantiating DensePDInteractionGraph
protocols.relax.FastRelax: CMD: repack  -1109.09  0  0  0.14575
protocols.relax.FastRelax: CMD: scale:fa_rep  -1091.66  0  0  0.154
protocols.relax.FastRelax: CMD: min  -1091.74  0  0  0.154
protocols.relax.FastRelax: CMD: coord_cst_weight  -1091.74  0  0  0.154
protocols.relax.FastRelax: CMD: scale:fa_rep  -767.633  0  0  0.30745
core.pack.task: Packer task: initialize from command line()
core.pack.pack_rotamers: built 8568 rotamers at 584 positions.
core.pack.interaction_graph.interaction_graph_factory: Instantiating DensePDInteractionGraph
protocols.relax.FastRelax: CMD: repack  -779.413  0  0  0.30745
protocols.relax.FastRelax: CMD: scale:fa_rep  -755.11  0  0  0.31955
protocols.relax.FastRelax: CMD: min  -809.471  0  0  0.31955
protocols.relax.FastRelax: CMD: coord_cst_weight  -809.471  0  0  0.31955
protocols.relax.FastRelax: CMD: scale:fa_rep  -391.969  0  0  0.55
core.pack.task: Packer task: initialize from command line()
core.pack.pack_rotamers: built 8243 rotamers at 584 positions.
core.pack.interaction_graph.interaction_graph_factory: Instantiating DensePDInteractionGraph
protocols.relax.FastRelax: CMD: repack  -392.427  0  0  0.55
protocols.relax.FastRelax: CMD: min  -435.295  0  0  0.55
protocols.relax.FastRelax: MRP: 0  -435.295  -435.295  0  0
protocols.relax.FastRelax: CMD: accept_to_best  -435.295  0  0  0.55
protocols.relax.FastRelax: CMD: endrepeat  -435.295  0  0  0.55
protocols.relax.FastRelax: CMD: coord_cst_weight  -435.295  0  0  0.55
protocols.relax.FastRelax: CMD: scale:fa_rep  -1333.32  0  0  0.022
core.pack.task: Packer task: initialize from command line()
core.pack.pack_rotamers: built 10129 rotamers at 584 positions.
core.pack.interaction_graph.interaction_graph_factory: Instantiating DensePDInteractionGraph
protocols.relax.FastRelax: CMD: repack  -1413.71  0  0  0.022
protocols.relax.FastRelax: CMD: scale:fa_rep  -1394.23  0  0  0.02805
protocols.relax.FastRelax: CMD: min  -1394.27  0  0  0.02805
protocols.relax.FastRelax: CMD: coord_cst_weight  -1394.27  0  0  0.02805
protocols.relax.FastRelax: CMD: scale:fa_rep  -1015.33  0  0  0.14575
core.pack.task: Packer task: initialize from command line()
core.pack.pack_rotamers: built 9049 rotamers at 584 positions.
core.pack.interaction_graph.interaction_graph_factory: Instantiating DensePDInteractionGraph
protocols.relax.FastRelax: CMD: repack  -1105.34  0  0  0.14575
protocols.relax.FastRelax: CMD: scale:fa_rep  -1087.53  0  0  0.154
protocols.relax.FastRelax: CMD: min  -1087.62  0  0  0.154
protocols.relax.FastRelax: CMD: coord_cst_weight  -1087.62  0  0  0.154
protocols.relax.FastRelax: CMD: scale:fa_rep  -756.506  0  0  0.30745
core.pack.task: Packer task: initialize from command line()
core.pack.pack_rotamers: built 8568 rotamers at 584 positions.
core.pack.interaction_graph.interaction_graph_factory: Instantiating DensePDInteractionGraph
protocols.relax.FastRelax: CMD: repack  -781.299  0  0  0.30745
protocols.relax.FastRelax: CMD: scale:fa_rep  -757.23  0  0  0.31955
protocols.relax.FastRelax: CMD: min  -805.818  0  0  0.31955
protocols.relax.FastRelax: CMD: coord_cst_weight  -805.818  0  0  0.31955
protocols.relax.FastRelax: CMD: scale:fa_rep  -390.395  0  0  0.55
core.pack.task: Packer task: initialize from command line()
core.pack.pack_rotamers: built 8243 rotamers at 584 positions.
core.pack.interaction_graph.interaction_graph_factory: Instantiating DensePDInteractionGraph
protocols.relax.FastRelax: CMD: repack  -391.74  0  0  0.55
protocols.relax.FastRelax: CMD: min  -430.286  0  0  0.55
protocols.relax.FastRelax: MRP: 1  -430.286  -435.295  0  0
protocols.relax.FastRelax: CMD: accept_to_best  -430.286  0  0  0.55
protocols.relax.FastRelax: CMD: endrepeat  -430.286  0  0  0.55
protocols.relax.FastRelax: CMD: coord_cst_weight  -430.286  0  0  0.55
protocols.relax.FastRelax: CMD: scale:fa_rep  -1329.04  0  0  0.022
core.pack.task: Packer task: initialize from command line()
core.pack.pack_rotamers: built 10129 rotamers at 584 positions.
core.pack.interaction_graph.interaction_graph_factory: Instantiating DensePDInteractionGraph
protocols.relax.FastRelax: CMD: repack  -1413.2  0  0  0.022
protocols.relax.FastRelax: CMD: scale:fa_rep  -1393.25  0  0  0.02805
protocols.relax.FastRelax: CMD: min  -1393.28  0  0  0.02805
protocols.relax.FastRelax: CMD: coord_cst_weight  -1393.28  0  0  0.02805
protocols.relax.FastRelax: CMD: scale:fa_rep  -1005.25  0  0  0.14575
core.pack.task: Packer task: initialize from command line()
core.pack.pack_rotamers: built 9049 rotamers at 584 positions.
core.pack.interaction_graph.interaction_graph_factory: Instantiating DensePDInteractionGraph
protocols.relax.FastRelax: CMD: repack  -1104.18  0  0  0.14575
protocols.relax.FastRelax: CMD: scale:fa_rep  -1086.68  0  0  0.154
protocols.relax.FastRelax: CMD: min  -1086.75  0  0  0.154
protocols.relax.FastRelax: CMD: coord_cst_weight  -1086.75  0  0  0.154
protocols.relax.FastRelax: CMD: scale:fa_rep  -761.305  0  0  0.30745
core.pack.task: Packer task: initialize from command line()
core.pack.pack_rotamers: built 8568 rotamers at 584 positions.
core.pack.interaction_graph.interaction_graph_factory: Instantiating DensePDInteractionGraph
protocols.relax.FastRelax: CMD: repack  -776.378  0  0  0.30745
protocols.relax.FastRelax: CMD: scale:fa_rep  -752.354  0  0  0.31955
protocols.relax.FastRelax: CMD: min  -804.359  0  0  0.31955
protocols.relax.FastRelax: CMD: coord_cst_weight  -804.359  0  0  0.31955
protocols.relax.FastRelax: CMD: scale:fa_rep  -391.837  0  0  0.55
core.pack.task: Packer task: initialize from command line()
core.pack.pack_rotamers: built 8243 rotamers at 584 positions.
core.pack.interaction_graph.interaction_graph_factory: Instantiating DensePDInteractionGraph
protocols.relax.FastRelax: CMD: repack  -392.323  0  0  0.55
protocols.relax.FastRelax: CMD: min  -426.916  0  0  0.55
protocols.relax.FastRelax: MRP: 2  -426.916  -435.295  0  0
protocols.relax.FastRelax: CMD: accept_to_best  -426.916  0  0  0.55
protocols.relax.FastRelax: CMD: endrepeat  -426.916  0  0  0.55
protocols.relax.FastRelax: CMD: coord_cst_weight  -426.916  0  0  0.55
protocols.relax.FastRelax: CMD: scale:fa_rep  -1324.21  0  0  0.022
core.pack.task: Packer task: initialize from command line()
core.pack.pack_rotamers: built 10129 rotamers at 584 positions.
core.pack.interaction_graph.interaction_graph_factory: Instantiating DensePDInteractionGraph
protocols.relax.FastRelax: CMD: repack  -1408.45  0  0  0.022
protocols.relax.FastRelax: CMD: scale:fa_rep  -1388.73  0  0  0.02805
protocols.relax.FastRelax: CMD: min  -1388.77  0  0  0.02805
protocols.relax.FastRelax: CMD: coord_cst_weight  -1388.77  0  0  0.02805
protocols.relax.FastRelax: CMD: scale:fa_rep  -1005.2  0  0  0.14575
core.pack.task: Packer task: initialize from command line()
core.pack.pack_rotamers: built 9049 rotamers at 584 positions.
core.pack.interaction_graph.interaction_graph_factory: Instantiating DensePDInteractionGraph
protocols.relax.FastRelax: CMD: repack  -1107.96  0  0  0.14575
protocols.relax.FastRelax: CMD: scale:fa_rep  -1090.55  0  0  0.154
protocols.relax.FastRelax: CMD: min  -1090.61  0  0  0.154
protocols.relax.FastRelax: CMD: coord_cst_weight  -1090.61  0  0  0.154
protocols.relax.FastRelax: CMD: scale:fa_rep  -766.773  0  0  0.30745
core.pack.task: Packer task: initialize from command line()
core.pack.pack_rotamers: built 8568 rotamers at 584 positions.
core.pack.interaction_graph.interaction_graph_factory: Instantiating DensePDInteractionGraph
protocols.relax.FastRelax: CMD: repack  -779.387  0  0  0.30745
protocols.relax.FastRelax: CMD: scale:fa_rep  -755.307  0  0  0.31955
protocols.relax.FastRelax: CMD: min  -808.877  0  0  0.31955
protocols.relax.FastRelax: CMD: coord_cst_weight  -808.877  0  0  0.31955
protocols.relax.FastRelax: CMD: scale:fa_rep  -396.751  0  0  0.55
core.pack.task: Packer task: initialize from command line()
core.pack.pack_rotamers: built 8243 rotamers at 584 positions.
core.pack.interaction_graph.interaction_graph_factory: Instantiating DensePDInteractionGraph
protocols.relax.FastRelax: CMD: repack  -398.062  0  0  0.55
protocols.relax.FastRelax: CMD: min  -430.452  0  0  0.55
protocols.relax.FastRelax: MRP: 3  -430.452  -435.295  0  0
protocols.relax.FastRelax: CMD: accept_to_best  -430.452  0  0  0.55
protocols.relax.FastRelax: CMD: endrepeat  -430.452  0  0  0.55
protocols.relax.FastRelax: CMD: coord_cst_weight  -430.452  0  0  0.55
protocols.relax.FastRelax: CMD: scale:fa_rep  -1325.66  0  0  0.022
core.pack.task: Packer task: initialize from command line()
core.pack.pack_rotamers: built 10129 rotamers at 584 positions.
core.pack.interaction_graph.interaction_graph_factory: Instantiating DensePDInteractionGraph
protocols.relax.FastRelax: CMD: repack  -1414.08  0  0  0.022
protocols.relax.FastRelax: CMD: scale:fa_rep  -1394.16  0  0  0.02805
protocols.relax.FastRelax: CMD: min  -1394.2  0  0  0.02805
protocols.relax.FastRelax: CMD: coord_cst_weight  -1394.2  0  0  0.02805
protocols.relax.FastRelax: CMD: scale:fa_rep  -1006.8  0  0  0.14575
core.pack.task: Packer task: initialize from command line()
core.pack.pack_rotamers: built 9049 rotamers at 584 positions.
core.pack.interaction_graph.interaction_graph_factory: Instantiating DensePDInteractionGraph
protocols.relax.FastRelax: CMD: repack  -1108.26  0  0  0.14575
protocols.relax.FastRelax: CMD: scale:fa_rep  -1090.74  0  0  0.154
protocols.relax.FastRelax: CMD: min  -1090.81  0  0  0.154
protocols.relax.FastRelax: CMD: coord_cst_weight  -1090.81  0  0  0.154
protocols.relax.FastRelax: CMD: scale:fa_rep  -764.981  0  0  0.30745
core.pack.task: Packer task: initialize from command line()
core.pack.pack_rotamers: built 8568 rotamers at 584 positions.
core.pack.interaction_graph.interaction_graph_factory: Instantiating DensePDInteractionGraph
protocols.relax.FastRelax: CMD: repack  -778.585  0  0  0.30745
protocols.relax.FastRelax: CMD: scale:fa_rep  -754.444  0  0  0.31955
protocols.relax.FastRelax: CMD: min  -805.92  0  0  0.31955
protocols.relax.FastRelax: CMD: coord_cst_weight  -805.92  0  0  0.31955
protocols.relax.FastRelax: CMD: scale:fa_rep  -390.745  0  0  0.55
core.pack.task: Packer task: initialize from command line()
core.pack.pack_rotamers: built 8243 rotamers at 584 positions.
core.pack.interaction_graph.interaction_graph_factory: Instantiating DensePDInteractionGraph
protocols.relax.FastRelax: CMD: repack  -391.388  0  0  0.55
protocols.relax.FastRelax: CMD: min  -429.599  0  0  0.55
protocols.relax.FastRelax: MRP: 4  -429.599  -435.295  0  0
protocols.relax.FastRelax: CMD: accept_to_best  -429.599  0  0  0.55
protocols.relax.FastRelax: CMD: endrepeat  -429.599  0  0  0.55
protocols::checkpoint: Deleting checkpoints of FastRelax
```

Out[18]:

```
True
```

In [19]:

```
rG17D = pyrosetta.pose_from_pdb("pdb/5T5K.relax_G17D.pdb")
sfxn.show(rG17D)
```

```
core.import_pose.import_pose: File 'pdb/5T5K.relax_G17D.pdb' automatically determined to be of type PDB
core.scoring.ScoreFunction: 
------------------------------------------------------------
 Scores                       Weight   Raw Score Wghtd.Score
------------------------------------------------------------
 fa_atr                       1.000   -4742.495   -4742.495
 fa_rep                       0.550    1700.810     935.446
 fa_sol                       1.000    4232.499    4232.499
 fa_intra_rep                 0.005    2697.160      13.486
 fa_intra_sol_xover4          1.000     526.758     526.758
 lk_ball_wtd                  1.000    -273.795    -273.795
 fa_elec                      1.000   -1195.722   -1195.722
 pro_close                    1.250       1.126       1.408
 hbond_sr_bb                  1.000    -221.670    -221.670
 hbond_lr_bb                  1.000     -20.687     -20.687
 hbond_bb_sc                  1.000     -31.787     -31.787
 hbond_sc                     1.000    -304.903    -304.903
 dslf_fa13                    1.250       0.000       0.000
 omega                        0.400      90.755      36.302
 fa_dun                       0.700     738.799     517.160
 p_aa_pp                      0.600     -59.222     -35.533
 yhh_planarity                0.625       0.000       0.000
 ref                          1.000     133.225     133.225
 rama_prepro                  0.450     -11.219      -5.049
---------------------------------------------------
 Total weighted score:                     -435.359
```

In [20]:

```
scrtype1 = pyrosetta.rosetta.core.scoring.ScoreType(1) #fa_atr = <ScoreType.fa_atr: 1>
scrtype2 = pyrosetta.rosetta.core.scoring.ScoreType(2) #fa_rep = <ScoreType.fa_rep: 2>
```

In [21]:

```
pyrosetta.toolbox.atom_pair_energy.print_residue_pair_energies(19, rpose, sfxn, scrtype1, 0)
```

```
I 12  -0.0033862179416992017
K 13  -0.013405502726086739
A 17  -0.9216622837498751
E 18  -1.344548304770868
R 19  -15.916102976398092
V 20  -1.0322908943796338
S 21  -0.15229937204056407
R 25  -1.0535897924934812e-08
K 120  -0.7437189862779889
T 121  -3.136808887802333
I 122  -1.2317054506203287
K 123  -0.00018165763962618708
c 473  -0.010957907138650589
a 474  -3.445532279991365
a 475  -3.2146618142038186
t 517  -4.213638012725424e-09
g 518  -0.05111475629821421
a 519  -0.4463404505781464
g 520  -1.5556996427953798
c 521  -0.018022089399829834
```

In [22]:

```
pyrosetta.toolbox.atom_pair_energy.print_residue_pair_energies(19, R20S, sfxn, scrtype1, 0)
```

```
I 12  -0.0033862179416992017
K 13  -0.013405502726086739
A 17  -0.9210262641110226
E 18  -0.848018573698188
S 19  -6.27918863870306
V 20  -0.999961956039603
S 21  -0.15246897638735776
R 25  -1.0535897924934812e-08
K 120  -0.6343150587509255
T 121  -2.57648816045366
I 122  -1.2308686348245461
K 123  -0.00018165763962618708
a 474  -0.9437619525509374
a 475  -1.8714263379493792
g 520  -0.1024150657786686
c 521  -0.001265749837899205
```

In [24]:

```
pyrosetta.toolbox.atom_pair_energy.print_residue_pair_energies(54, rpose, sfxn, scrtype2, 0)
```

```
R 52  0.0023887396102304226
K 53  0.09261438870938402
T 54  9444.972394277309
I 55  0.41261487441705097
R 86  3.3880719150587706
V 87  0.00302579365215503
S 88  0.00043379247025197507
a 494  0.000461002221837533
a 501  4.458825123624806
```

In [25]:

```
pyrosetta.toolbox.atom_pair_energy.print_residue_pair_energies(54, rT55L, sfxn, scrtype2, 0)
```

```
R 52  0.0023887396102304226
K 53  2.113276666304838
L 54  11278.767737336157
I 55  0.05448626371712283
R 86  48.28779958914763
c 493  0.00483030964426804
a 494  16.8201940812143
a 501  0.1164268471155883
```

In [26]:

```
pyrosetta.toolbox.atom_pair_energy.print_residue_pair_energies(16, rpose, sfxn, scrtype2, 0)
```

```
D 14  0.24261654952235784
A 15  0.131362490943214
G 16  5482.113821572886
A 17  0.06608727665710067
I 111  0.3005267708743051
R 115  0.059212871505435485
```

In [27]:

```
pyrosetta.toolbox.atom_pair_energy.print_residue_pair_energies(16, rG17D, sfxn, scrtype2, 0)
```

```
D 14  0.24261654952235784
A 15  33.69856704218391
D 16  10263.253099215674
A 17  0.06608727665710067
I 111  0.7521219742007441
```

### To look at the energy associated with the L1-L1 interface we stacked the 5T5K.pdb file using the symmetry mates function in PyMOL.¶

In [28]:

```
cleanATOM('pdb/stacked_G17.pdb')
```

In [29]:

```
G17_stacked = pose_from_pdb('pdb/stacked_G17.clean.pdb')
```

```
core.import_pose.import_pose: File 'pdb/stacked_G17.clean.pdb' automatically determined to be of type PDB
core.conformation.Conformation: [ WARNING ] missing heavyatom:  OXT on residue PHE:CtermProteinFull 67
core.conformation.Conformation: [ WARNING ] missing heavyatom:  OXT on residue PHE:CtermProteinFull 134
core.conformation.Conformation: [ WARNING ] missing heavyatom:  OXT on residue LYS:CtermProteinFull 202
core.conformation.Conformation: [ WARNING ] missing heavyatom:  OXT on residue LYS:CtermProteinFull 270
core.conformation.Conformation: [ WARNING ] missing heavyatom:  OXT on residue LYS:CtermProteinFull 338
core.conformation.Conformation: [ WARNING ] missing heavyatom:  OXT on residue LYS:CtermProteinFull 406
core.conformation.Conformation: [ WARNING ] missing heavyatom:  OXT on residue PHE:CtermProteinFull 473
core.conformation.Conformation: [ WARNING ] missing heavyatom:  OXT on residue PHE:CtermProteinFull 540
core.conformation.Conformation: [ WARNING ] missing heavyatom:  OXT on residue PHE:CtermProteinFull 607
core.conformation.Conformation: [ WARNING ] missing heavyatom:  OXT on residue PHE:CtermProteinFull 674
core.conformation.Conformation: [ WARNING ] missing heavyatom:  OXT on residue PHE:CtermProteinFull 741
core.conformation.Conformation: [ WARNING ] missing heavyatom:  OXT on residue PHE:CtermProteinFull 808
```

In [30]:

```
movemap = MoveMap()
movemap.set_bb(False)
movemap.set_chi(True)
relax = pyrosetta.rosetta.protocols.relax.FastRelax()
relax.constrain_relax_to_start_coords(True)
relax.coord_constrain_sidechains(True)
relax.ramp_down_constraints(False)
relax.set_scorefxn(sfxn)
relax.set_movemap(movemap)
relax.apply(G17_stacked)
G17_stacked.dump_pdb('pdb/G17_stacked.relax.pdb')
```

```
core.scoring.ScoreFunctionFactory: SCOREFUNCTION: ref2015
protocols.relax: turning off DNA bb and chi move
protocols.relax: turning off DNA bb and chi move
protocols.relax: turning off DNA bb and chi move
protocols.relax: turning off DNA bb and chi move
protocols.relax: turning off DNA bb and chi move
protocols.relax: turning off DNA bb and chi move
protocols.relax: turning off DNA bb and chi move
protocols.relax: turning off DNA bb and chi move
protocols.relax: turning off DNA bb and chi move
protocols.relax: turning off DNA bb and chi move
protocols.relax: turning off DNA bb and chi move
protocols.relax: turning off DNA bb and chi move
protocols.relax: turning off DNA bb and chi move
protocols.relax: turning off DNA bb and chi move
protocols.relax: turning off DNA bb and chi move
protocols.relax: turning off DNA bb and chi move
protocols.relax: turning off DNA bb and chi move
protocols.relax: turning off DNA bb and chi move
protocols.relax: turning off DNA bb and chi move
protocols.relax: turning off DNA bb and chi move
protocols.relax: turning off DNA bb and chi move
protocols.relax: turning off DNA bb and chi move
protocols.relax: turning off DNA bb and chi move
protocols.relax: turning off DNA bb and chi move
protocols.relax: turning off DNA bb and chi move
protocols.relax: turning off DNA bb and chi move
protocols.relax: turning off DNA bb and chi move
protocols.relax: turning off DNA bb and chi move
protocols.relax: turning off DNA bb and chi move
protocols.relax: turning off DNA bb and chi move
protocols.relax: turning off DNA bb and chi move
protocols.relax: turning off DNA bb and chi move
protocols.relax: turning off DNA bb and chi move
protocols.relax: turning off DNA bb and chi move
protocols.relax: turning off DNA bb and chi move
protocols.relax: turning off DNA bb and chi move
protocols.relax: turning off DNA bb and chi move
protocols.relax: turning off DNA bb and chi move
protocols.relax: turning off DNA bb and chi move
protocols.relax: turning off DNA bb and chi move
protocols.relax: turning off DNA bb and chi move
protocols.relax: turning off DNA bb and chi move
protocols.relax: turning off DNA bb and chi move
protocols.relax: turning off DNA bb and chi move
protocols.relax: turning off DNA bb and chi move
protocols.relax: turning off DNA bb and chi move
protocols.relax: turning off DNA bb and chi move
protocols.relax: turning off DNA bb and chi move
protocols.relax: turning off DNA bb and chi move
protocols.relax: turning off DNA bb and chi move
protocols.relax: turning off DNA bb and chi move
protocols.relax: turning off DNA bb and chi move
protocols.relax: turning off DNA bb and chi move
protocols.relax: turning off DNA bb and chi move
protocols.relax: turning off DNA bb and chi move
protocols.relax: turning off DNA bb and chi move
protocols.relax: turning off DNA bb and chi move
protocols.relax: turning off DNA bb and chi move
protocols.relax: turning off DNA bb and chi move
protocols.relax: turning off DNA bb and chi move
protocols.relax: turning off DNA bb and chi move
protocols.relax: turning off DNA bb and chi move
protocols.relax: turning off DNA bb and chi move
protocols.relax: turning off DNA bb and chi move
protocols.relax: turning off DNA bb and chi move
protocols.relax: turning off DNA bb and chi move
protocols.relax: turning off DNA bb and chi move
protocols.relax: turning off DNA bb and chi move
protocols.relax: turning off DNA bb and chi move
protocols.relax: turning off DNA bb and chi move
protocols.relax: turning off DNA bb and chi move
protocols.relax: turning off DNA bb and chi move
protocols.relax: turning off DNA bb and chi move
protocols.relax: turning off DNA bb and chi move
protocols.relax: turning off DNA bb and chi move
protocols.relax: turning off DNA bb and chi move
protocols.relax: turning off DNA bb and chi move
protocols.relax: turning off DNA bb and chi move
protocols.relax: turning off DNA bb and chi move
protocols.relax: turning off DNA bb and chi move
protocols.relax: turning off DNA bb and chi move
protocols.relax: turning off DNA bb and chi move
protocols.relax: turning off DNA bb and chi move
protocols.relax: turning off DNA bb and chi move
protocols.relax: turning off DNA bb and chi move
protocols.relax: turning off DNA bb and chi move
protocols.relax: turning off DNA bb and chi move
protocols.relax: turning off DNA bb and chi move
protocols.relax: turning off DNA bb and chi move
protocols.relax: turning off DNA bb and chi move
protocols.relax: turning off DNA bb and chi move
protocols.relax: turning off DNA bb and chi move
protocols.relax: turning off DNA bb and chi move
protocols.relax: turning off DNA bb and chi move
protocols.relax: turning off DNA bb and chi move
protocols.relax: turning off DNA bb and chi move
protocols.relax: turning off DNA bb and chi move
protocols.relax: turning off DNA bb and chi move
protocols.relax: turning off DNA bb and chi move
protocols.relax: turning off DNA bb and chi move
protocols.relax: turning off DNA bb and chi move
protocols.relax: turning off DNA bb and chi move
protocols.relax: turning off DNA bb and chi move
protocols.relax: turning off DNA bb and chi move
protocols.relax: turning off DNA bb and chi move
protocols.relax: turning off DNA bb and chi move
protocols.relax: turning off DNA bb and chi move
protocols.relax: turning off DNA bb and chi move
protocols.relax: turning off DNA bb and chi move
protocols.relax: turning off DNA bb and chi move
protocols.relax: turning off DNA bb and chi move
protocols.relax: turning off DNA bb and chi move
protocols.relax: turning off DNA bb and chi move
protocols.relax: turning off DNA bb and chi move
protocols.relax: turning off DNA bb and chi move
protocols.relax: turning off DNA bb and chi move
protocols.relax: turning off DNA bb and chi move
protocols.relax: turning off DNA bb and chi move
protocols.relax: turning off DNA bb and chi move
protocols.relax: turning off DNA bb and chi move
protocols.relax: turning off DNA bb and chi move
protocols.relax: turning off DNA bb and chi move
protocols.relax: turning off DNA bb and chi move
protocols.relax: turning off DNA bb and chi move
protocols.relax: turning off DNA bb and chi move
protocols.relax: turning off DNA bb and chi move
protocols.relax: turning off DNA bb and chi move
protocols.relax: turning off DNA bb and chi move
protocols.relax: turning off DNA bb and chi move
protocols.relax: turning off DNA bb and chi move
protocols.relax: turning off DNA bb and chi move
protocols.relax: turning off DNA bb and chi move
protocols.relax: turning off DNA bb and chi move
protocols.relax: turning off DNA bb and chi move
protocols.relax: turning off DNA bb and chi move
protocols.relax: turning off DNA bb and chi move
protocols.relax: turning off DNA bb and chi move
protocols.relax: turning off DNA bb and chi move
protocols.relax: turning off DNA bb and chi move
protocols.relax: turning off DNA bb and chi move
protocols.relax: turning off DNA bb and chi move
protocols.relax: turning off DNA bb and chi move
protocols.relax: turning off DNA bb and chi move
protocols.relax: turning off DNA bb and chi move
protocols.relax: turning off DNA bb and chi move
protocols.relax: turning off DNA bb and chi move
protocols.relax: turning off DNA bb and chi move
protocols.relax: turning off DNA bb and chi move
protocols.relax: turning off DNA bb and chi move
protocols.relax: turning off DNA bb and chi move
protocols.relax: turning off DNA bb and chi move
protocols.relax: turning off DNA bb and chi move
protocols.relax: turning off DNA bb and chi move
protocols.relax: turning off DNA bb and chi move
protocols.relax: turning off DNA bb and chi move
protocols.relax: turning off DNA bb and chi move
protocols.relax: turning off DNA bb and chi move
protocols.relax: turning off DNA bb and chi move
protocols.relax: turning off DNA bb and chi move
protocols.relax: turning off DNA bb and chi move
protocols.relax: turning off DNA bb and chi move
protocols.relax: turning off DNA bb and chi move
protocols.relax: turning off DNA bb and chi move
protocols.relax: turning off DNA bb and chi move
protocols.relax: turning off DNA bb and chi move
protocols.relax: turning off DNA bb and chi move
protocols.relax: turning off DNA bb and chi move
protocols.relax: turning off DNA bb and chi move
protocols.relax: turning off DNA bb and chi move
protocols.relax: turning off DNA bb and chi move
protocols.relax: turning off DNA bb and chi move
protocols.relax: turning off DNA bb and chi move
protocols.relax: turning off DNA bb and chi move
protocols.relax: turning off DNA bb and chi move
protocols.relax: turning off DNA bb and chi move
protocols.relax: turning off DNA bb and chi move
protocols.relax: turning off DNA bb and chi move
protocols.relax: turning off DNA bb and chi move
protocols.relax: turning off DNA bb and chi move
protocols.relax: turning off DNA bb and chi move
protocols.relax: turning off DNA bb and chi move
protocols.relax: turning off DNA bb and chi move
protocols.relax: turning off DNA bb and chi move
protocols.relax: turning off DNA bb and chi move
protocols.relax: turning off DNA bb and chi move
protocols.relax: turning off DNA bb and chi move
protocols.relax: turning off DNA bb and chi move
protocols.relax: turning off DNA bb and chi move
protocols.relax: turning off DNA bb and chi move
protocols.relax: turning off DNA bb and chi move
protocols.relax: turning off DNA bb and chi move
protocols.relax: turning off DNA bb and chi move
protocols.relax: turning off DNA bb and chi move
protocols.relax: turning off DNA bb and chi move
protocols.relax: turning off DNA bb and chi move
protocols.relax: turning off DNA bb and chi move
protocols.relax: turning off DNA bb and chi move
protocols.relax: turning off DNA bb and chi move
protocols.relax: turning off DNA bb and chi move
protocols.relax: turning off DNA bb and chi move
protocols.relax: turning off DNA bb and chi move
protocols.relax: turning off DNA bb and chi move
protocols.relax: turning off DNA bb and chi move
protocols.relax: turning off DNA bb and chi move
protocols.relax: turning off DNA bb and chi move
protocols.relax: turning off DNA bb and chi move
protocols.relax: turning off DNA bb and chi move
protocols.relax: turning off DNA bb and chi move
protocols.relax: turning off DNA bb and chi move
protocols.relax: turning off DNA bb and chi move
protocols.relax: turning off DNA bb and chi move
protocols.relax: turning off DNA bb and chi move
protocols.relax: turning off DNA bb and chi move
protocols.relax: turning off DNA bb and chi move
protocols.relax: turning off DNA bb and chi move
protocols.relax: turning off DNA bb and chi move
protocols.relax: turning off DNA bb and chi move
protocols.relax: turning off DNA bb and chi move
protocols.relax: turning off DNA bb and chi move
protocols.relax: turning off DNA bb and chi move
protocols.relax: turning off DNA bb and chi move
protocols.relax: turning off DNA bb and chi move
protocols.relax: turning off DNA bb and chi move
protocols.relax: turning off DNA bb and chi move
protocols.relax: turning off DNA bb and chi move
protocols.relax: turning off DNA bb and chi move
protocols.relax: turning off DNA bb and chi move
protocols.relax: turning off DNA bb and chi move
protocols.relax: turning off DNA bb and chi move
protocols.relax: turning off DNA bb and chi move
protocols.relax: turning off DNA bb and chi move
protocols.relax: turning off DNA bb and chi move
protocols.relax: turning off DNA bb and chi move
protocols.relax: turning off DNA bb and chi move
protocols.relax: turning off DNA bb and chi move
protocols.relax: turning off DNA bb and chi move
protocols.relax: turning off DNA bb and chi move
protocols.relax: turning off DNA bb and chi move
protocols.relax: turning off DNA bb and chi move
protocols.relax: turning off DNA bb and chi move
protocols.relax: turning off DNA bb and chi move
protocols.relax: turning off DNA bb and chi move
protocols.relax: turning off DNA bb and chi move
protocols.relax: turning off DNA bb and chi move
protocols.relax: turning off DNA bb and chi move
protocols.relax: turning off DNA bb and chi move
protocols.relax: turning off DNA bb and chi move
protocols.relax: turning off DNA bb and chi move
protocols.relax: turning off DNA bb and chi move
protocols.relax: turning off DNA bb and chi move
protocols.relax: turning off DNA bb and chi move
protocols.relax: turning off DNA bb and chi move
protocols.relax: turning off DNA bb and chi move
protocols.relax: turning off DNA bb and chi move
protocols.relax: turning off DNA bb and chi move
protocols.relax: turning off DNA bb and chi move
protocols.relax: turning off DNA bb and chi move
protocols.relax: turning off DNA bb and chi move
protocols.relax: turning off DNA bb and chi move
protocols.relax: turning off DNA bb and chi move
protocols.relax: turning off DNA bb and chi move
protocols.relax: turning off DNA bb and chi move
protocols.relax: turning off DNA bb and chi move
protocols.relax: turning off DNA bb and chi move
protocols.relax: turning off DNA bb and chi move
protocols.relax: turning off DNA bb and chi move
protocols.relax: turning off DNA bb and chi move
protocols.relax: turning off DNA bb and chi move
protocols.relax: turning off DNA bb and chi move
protocols.relax: turning off DNA bb and chi move
protocols.relax: turning off DNA bb and chi move
protocols.relax: turning off DNA bb and chi move
protocols.relax: turning off DNA bb and chi move
protocols.relax: turning off DNA bb and chi move
protocols.relax: turning off DNA bb and chi move
protocols.relax: turning off DNA bb and chi move
protocols.relax: turning off DNA bb and chi move
protocols.relax: turning off DNA bb and chi move
protocols.relax: turning off DNA bb and chi move
protocols.relax: turning off DNA bb and chi move
protocols.relax: turning off DNA bb and chi move
protocols.relax: turning off DNA bb and chi move
protocols.relax: turning off DNA bb and chi move
protocols.relax: turning off DNA bb and chi move
protocols.relax: turning off DNA bb and chi move
protocols.relax: turning off DNA bb and chi move
protocols.relax: turning off DNA bb and chi move
protocols.relax: turning off DNA bb and chi move
protocols.relax: turning off DNA bb and chi move
protocols.relax: turning off DNA bb and chi move
protocols.relax: turning off DNA bb and chi move
protocols.relax: turning off DNA bb and chi move
protocols.relax: turning off DNA bb and chi move
protocols.relax: turning off DNA bb and chi move
protocols.relax: turning off DNA bb and chi move
protocols.relax: turning off DNA bb and chi move
protocols.relax: turning off DNA bb and chi move
protocols.relax: turning off DNA bb and chi move
protocols.relax: turning off DNA bb and chi move
protocols.relax: turning off DNA bb and chi move
protocols.relax: turning off DNA bb and chi move
protocols.relax: turning off DNA bb and chi move
protocols.relax: turning off DNA bb and chi move
protocols.relax: turning off DNA bb and chi move
protocols.relax: turning off DNA bb and chi move
protocols.relax: turning off DNA bb and chi move
protocols.relax: turning off DNA bb and chi move
protocols.relax: turning off DNA bb and chi move
protocols.relax: turning off DNA bb and chi move
protocols.relax: turning off DNA bb and chi move
protocols.relax: turning off DNA bb and chi move
protocols.relax: turning off DNA bb and chi move
protocols.relax: turning off DNA bb and chi move
protocols.relax: turning off DNA bb and chi move
protocols.relax: turning off DNA bb and chi move
protocols.relax: turning off DNA bb and chi move
protocols.relax: turning off DNA bb and chi move
protocols.relax: turning off DNA bb and chi move
protocols.relax: turning off DNA bb and chi move
protocols.relax: turning off DNA bb and chi move
protocols.relax: turning off DNA bb and chi move
protocols.relax: turning off DNA bb and chi move
protocols.relax: turning off DNA bb and chi move
protocols.relax: turning off DNA bb and chi move
protocols.relax: turning off DNA bb and chi move
protocols.relax: turning off DNA bb and chi move
protocols.relax: turning off DNA bb and chi move
protocols.relax: turning off DNA bb and chi move
protocols.relax: turning off DNA bb and chi move
protocols.relax: turning off DNA bb and chi move
protocols.relax: turning off DNA bb and chi move
protocols.relax: turning off DNA bb and chi move
protocols.relax: turning off DNA bb and chi move
protocols.relax: turning off DNA bb and chi move
protocols.relax: turning off DNA bb and chi move
protocols.relax: turning off DNA bb and chi move
protocols.relax: turning off DNA bb and chi move
protocols.relax: turning off DNA bb and chi move
protocols.relax: turning off DNA bb and chi move
protocols.relax: turning off DNA bb and chi move
protocols.relax: turning off DNA bb and chi move
protocols.relax: turning off DNA bb and chi move
protocols.relax: turning off DNA bb and chi move
protocols.relax: turning off DNA bb and chi move
protocols.relax: turning off DNA bb and chi move
protocols.relax: turning off DNA bb and chi move
protocols.relax: turning off DNA bb and chi move
protocols.relax: turning off DNA bb and chi move
protocols.relax: turning off DNA bb and chi move
protocols.relax: turning off DNA bb and chi move
protocols.relax: turning off DNA bb and chi move
protocols.relax: turning off DNA bb and chi move
protocols.relax: turning off DNA bb and chi move
protocols.relax: turning off DNA bb and chi move
protocols.relax: turning off DNA bb and chi move
protocols.relax: turning off DNA bb and chi move
protocols.relax: turning off DNA bb and chi move
protocols.relax: turning off DNA bb and chi move
protocols.relax: turning off DNA bb and chi move
protocols.relax: turning off DNA bb and chi move
protocols.relax.FastRelax: CMD: repeat  472.18  0  0  0.55
protocols.relax.FastRelax: CMD: coord_cst_weight  472.18  0  0  0.55
protocols.relax.FastRelax: CMD: scale:fa_rep  -1521.88  0  0  0.022
core.pack.task: Packer task: initialize from command line()
core.pack.pack_rotamers: built 22026 rotamers at 1168 positions.
core.pack.interaction_graph.interaction_graph_factory: Instantiating DensePDInteractionGraph
protocols.relax.FastRelax: CMD: repack  -2830.07  0  0  0.022
protocols.relax.FastRelax: CMD: scale:fa_rep  -2784.7  0  0  0.02805
protocols.relax.FastRelax: CMD: min  -2784.86  0  0  0.02805
protocols.relax.FastRelax: CMD: coord_cst_weight  -2784.86  0  0  0.02805
protocols.relax.FastRelax: CMD: scale:fa_rep  -1902.34  0  0  0.14575
core.pack.task: Packer task: initialize from command line()
core.pack.pack_rotamers: built 19691 rotamers at 1168 positions.
core.pack.interaction_graph.interaction_graph_factory: Instantiating DensePDInteractionGraph
protocols.relax.FastRelax: CMD: repack  -2199.45  0  0  0.14575
protocols.relax.FastRelax: CMD: scale:fa_rep  -2162.99  0  0  0.154
protocols.relax.FastRelax: CMD: min  -2163.24  0  0  0.154
protocols.relax.FastRelax: CMD: coord_cst_weight  -2163.24  0  0  0.154
protocols.relax.FastRelax: CMD: scale:fa_rep  -1485.27  0  0  0.30745
core.pack.task: Packer task: initialize from command line()
core.pack.pack_rotamers: built 18630 rotamers at 1168 positions.
core.pack.interaction_graph.interaction_graph_factory: Instantiating DensePDInteractionGraph
protocols.relax.FastRelax: CMD: repack  -1541.34  0  0  0.30745
protocols.relax.FastRelax: CMD: scale:fa_rep  -1492.74  0  0  0.31955
protocols.relax.FastRelax: CMD: min  -1658.13  0  0  0.31955
protocols.relax.FastRelax: CMD: coord_cst_weight  -1658.13  0  0  0.31955
protocols.relax.FastRelax: CMD: scale:fa_rep  -841.886  0  0  0.55
core.pack.task: Packer task: initialize from command line()
core.pack.pack_rotamers: built 17880 rotamers at 1168 positions.
core.pack.interaction_graph.interaction_graph_factory: Instantiating DensePDInteractionGraph
protocols.relax.FastRelax: CMD: repack  -835.097  0  0  0.55
protocols.relax.FastRelax: CMD: min  -922.799  0  0  0.55
protocols.relax.FastRelax: MRP: 0  -922.799  -922.799  0  0
protocols.relax.FastRelax: CMD: accept_to_best  -922.799  0  0  0.55
protocols.relax.FastRelax: CMD: endrepeat  -922.799  0  0  0.55
protocols.relax.FastRelax: CMD: coord_cst_weight  -922.799  0  0  0.55
protocols.relax.FastRelax: CMD: scale:fa_rep  -2704.3  0  0  0.022
core.pack.task: Packer task: initialize from command line()
core.pack.pack_rotamers: built 22073 rotamers at 1168 positions.
core.pack.interaction_graph.interaction_graph_factory: Instantiating DensePDInteractionGraph
protocols.relax.FastRelax: CMD: repack  -2879.87  0  0  0.022
protocols.relax.FastRelax: CMD: scale:fa_rep  -2837.98  0  0  0.02805
protocols.relax.FastRelax: CMD: min  -2838.07  0  0  0.02805
protocols.relax.FastRelax: CMD: coord_cst_weight  -2838.07  0  0  0.02805
protocols.relax.FastRelax: CMD: scale:fa_rep  -2023.19  0  0  0.14575
core.pack.task: Packer task: initialize from command line()
core.pack.pack_rotamers: built 19691 rotamers at 1168 positions.
core.pack.interaction_graph.interaction_graph_factory: Instantiating DensePDInteractionGraph
protocols.relax.FastRelax: CMD: repack  -2266.92  0  0  0.14575
protocols.relax.FastRelax: CMD: scale:fa_rep  -2232.51  0  0  0.154
protocols.relax.FastRelax: CMD: min  -2232.65  0  0  0.154
protocols.relax.FastRelax: CMD: coord_cst_weight  -2232.65  0  0  0.154
protocols.relax.FastRelax: CMD: scale:fa_rep  -1592.76  0  0  0.30745
core.pack.task: Packer task: initialize from command line()
core.pack.pack_rotamers: built 18630 rotamers at 1168 positions.
core.pack.interaction_graph.interaction_graph_factory: Instantiating DensePDInteractionGraph
protocols.relax.FastRelax: CMD: repack  -1618.51  0  0  0.30745
protocols.relax.FastRelax: CMD: scale:fa_rep  -1571.13  0  0  0.31955
protocols.relax.FastRelax: CMD: min  -1673.94  0  0  0.31955
protocols.relax.FastRelax: CMD: coord_cst_weight  -1673.94  0  0  0.31955
protocols.relax.FastRelax: CMD: scale:fa_rep  -850.291  0  0  0.55
core.pack.task: Packer task: initialize from command line()
core.pack.pack_rotamers: built 17880 rotamers at 1168 positions.
core.pack.interaction_graph.interaction_graph_factory: Instantiating DensePDInteractionGraph
protocols.relax.FastRelax: CMD: repack  -855.294  0  0  0.55
protocols.relax.FastRelax: CMD: min  -935.684  0  0  0.55
protocols.relax.FastRelax: MRP: 1  -935.684  -935.684  0  0
protocols.relax.FastRelax: CMD: accept_to_best  -935.684  0  0  0.55
protocols.relax.FastRelax: CMD: endrepeat  -935.684  0  0  0.55
protocols.relax.FastRelax: CMD: coord_cst_weight  -935.684  0  0  0.55
protocols.relax.FastRelax: CMD: scale:fa_rep  -2715.7  0  0  0.022
core.pack.task: Packer task: initialize from command line()
core.pack.pack_rotamers: built 22073 rotamers at 1168 positions.
core.pack.interaction_graph.interaction_graph_factory: Instantiating DensePDInteractionGraph
protocols.relax.FastRelax: CMD: repack  -2876.2  0  0  0.022
protocols.relax.FastRelax: CMD: scale:fa_rep  -2836.02  0  0  0.02805
protocols.relax.FastRelax: CMD: min  -2836.1  0  0  0.02805
protocols.relax.FastRelax: CMD: coord_cst_weight  -2836.1  0  0  0.02805
protocols.relax.FastRelax: CMD: scale:fa_rep  -2054.45  0  0  0.14575
core.pack.task: Packer task: initialize from command line()
core.pack.pack_rotamers: built 19691 rotamers at 1168 positions.
core.pack.interaction_graph.interaction_graph_factory: Instantiating DensePDInteractionGraph
protocols.relax.FastRelax: CMD: repack  -2275.91  0  0  0.14575
protocols.relax.FastRelax: CMD: scale:fa_rep  -2241.8  0  0  0.154
protocols.relax.FastRelax: CMD: min  -2241.93  0  0  0.154
protocols.relax.FastRelax: CMD: coord_cst_weight  -2241.93  0  0  0.154
protocols.relax.FastRelax: CMD: scale:fa_rep  -1607.52  0  0  0.30745
core.pack.task: Packer task: initialize from command line()
core.pack.pack_rotamers: built 18630 rotamers at 1168 positions.
core.pack.interaction_graph.interaction_graph_factory: Instantiating DensePDInteractionGraph
protocols.relax.FastRelax: CMD: repack  -1647.6  0  0  0.30745
protocols.relax.FastRelax: CMD: scale:fa_rep  -1601.05  0  0  0.31955
protocols.relax.FastRelax: CMD: min  -1679.09  0  0  0.31955
protocols.relax.FastRelax: CMD: coord_cst_weight  -1679.09  0  0  0.31955
protocols.relax.FastRelax: CMD: scale:fa_rep  -851.782  0  0  0.55
core.pack.task: Packer task: initialize from command line()
core.pack.pack_rotamers: built 17880 rotamers at 1168 positions.
core.pack.interaction_graph.interaction_graph_factory: Instantiating DensePDInteractionGraph
protocols.relax.FastRelax: CMD: repack  -842.604  0  0  0.55
protocols.relax.FastRelax: CMD: min  -929.972  0  0  0.55
protocols.relax.FastRelax: MRP: 2  -929.972  -935.684  0  0
protocols.relax.FastRelax: CMD: accept_to_best  -929.972  0  0  0.55
protocols.relax.FastRelax: CMD: endrepeat  -929.972  0  0  0.55
protocols.relax.FastRelax: CMD: coord_cst_weight  -929.972  0  0  0.55
protocols.relax.FastRelax: CMD: scale:fa_rep  -2713.1  0  0  0.022
core.pack.task: Packer task: initialize from command line()
core.pack.pack_rotamers: built 22073 rotamers at 1168 positions.
core.pack.interaction_graph.interaction_graph_factory: Instantiating DensePDInteractionGraph
protocols.relax.FastRelax: CMD: repack  -2882.27  0  0  0.022
protocols.relax.FastRelax: CMD: scale:fa_rep  -2841.55  0  0  0.02805
protocols.relax.FastRelax: CMD: min  -2841.63  0  0  0.02805
protocols.relax.FastRelax: CMD: coord_cst_weight  -2841.63  0  0  0.02805
protocols.relax.FastRelax: CMD: scale:fa_rep  -2049.62  0  0  0.14575
core.pack.task: Packer task: initialize from command line()
core.pack.pack_rotamers: built 19691 rotamers at 1168 positions.
core.pack.interaction_graph.interaction_graph_factory: Instantiating DensePDInteractionGraph
protocols.relax.FastRelax: CMD: repack  -2283.27  0  0  0.14575
protocols.relax.FastRelax: CMD: scale:fa_rep  -2249.36  0  0  0.154
protocols.relax.FastRelax: CMD: min  -2249.5  0  0  0.154
protocols.relax.FastRelax: CMD: coord_cst_weight  -2249.5  0  0  0.154
protocols.relax.FastRelax: CMD: scale:fa_rep  -1618.95  0  0  0.30745
core.pack.task: Packer task: initialize from command line()
core.pack.pack_rotamers: built 18630 rotamers at 1168 positions.
core.pack.interaction_graph.interaction_graph_factory: Instantiating DensePDInteractionGraph
protocols.relax.FastRelax: CMD: repack  -1640.59  0  0  0.30745
protocols.relax.FastRelax: CMD: scale:fa_rep  -1593.33  0  0  0.31955
protocols.relax.FastRelax: CMD: min  -1681.37  0  0  0.31955
protocols.relax.FastRelax: CMD: coord_cst_weight  -1681.37  0  0  0.31955
protocols.relax.FastRelax: CMD: scale:fa_rep  -850.349  0  0  0.55
core.pack.task: Packer task: initialize from command line()
core.pack.pack_rotamers: built 17880 rotamers at 1168 positions.
core.pack.interaction_graph.interaction_graph_factory: Instantiating DensePDInteractionGraph
protocols.relax.FastRelax: CMD: repack  -848.599  0  0  0.55
protocols.relax.FastRelax: CMD: min  -924.907  0  0  0.55
protocols.relax.FastRelax: MRP: 3  -924.907  -935.684  0  0
protocols.relax.FastRelax: CMD: accept_to_best  -924.907  0  0  0.55
protocols.relax.FastRelax: CMD: endrepeat  -924.907  0  0  0.55
protocols.relax.FastRelax: CMD: coord_cst_weight  -924.907  0  0  0.55
protocols.relax.FastRelax: CMD: scale:fa_rep  -2713.6  0  0  0.022
core.pack.task: Packer task: initialize from command line()
core.pack.pack_rotamers: built 22073 rotamers at 1168 positions.
core.pack.interaction_graph.interaction_graph_factory: Instantiating DensePDInteractionGraph
protocols.relax.FastRelax: CMD: repack  -2887.16  0  0  0.022
protocols.relax.FastRelax: CMD: scale:fa_rep  -2845.14  0  0  0.02805
protocols.relax.FastRelax: CMD: min  -2845.23  0  0  0.02805
protocols.relax.FastRelax: CMD: coord_cst_weight  -2845.23  0  0  0.02805
protocols.relax.FastRelax: CMD: scale:fa_rep  -2027.82  0  0  0.14575
core.pack.task: Packer task: initialize from command line()
core.pack.pack_rotamers: built 19691 rotamers at 1168 positions.
core.pack.interaction_graph.interaction_graph_factory: Instantiating DensePDInteractionGraph
protocols.relax.FastRelax: CMD: repack  -2281.09  0  0  0.14575
protocols.relax.FastRelax: CMD: scale:fa_rep  -2247.2  0  0  0.154
protocols.relax.FastRelax: CMD: min  -2247.32  0  0  0.154
protocols.relax.FastRelax: CMD: coord_cst_weight  -2247.32  0  0  0.154
protocols.relax.FastRelax: CMD: scale:fa_rep  -1617.12  0  0  0.30745
core.pack.task: Packer task: initialize from command line()
core.pack.pack_rotamers: built 18630 rotamers at 1168 positions.
core.pack.interaction_graph.interaction_graph_factory: Instantiating DensePDInteractionGraph
protocols.relax.FastRelax: CMD: repack  -1638.56  0  0  0.30745
protocols.relax.FastRelax: CMD: scale:fa_rep  -1591.76  0  0  0.31955
protocols.relax.FastRelax: CMD: min  -1679.97  0  0  0.31955
protocols.relax.FastRelax: CMD: coord_cst_weight  -1679.97  0  0  0.31955
protocols.relax.FastRelax: CMD: scale:fa_rep  -857.001  0  0  0.55
core.pack.task: Packer task: initialize from command line()
core.pack.pack_rotamers: built 17880 rotamers at 1168 positions.
core.pack.interaction_graph.interaction_graph_factory: Instantiating DensePDInteractionGraph
protocols.relax.FastRelax: CMD: repack  -857.101  0  0  0.55
protocols.relax.FastRelax: CMD: min  -933.45  0  0  0.55
protocols.relax.FastRelax: MRP: 4  -933.45  -935.684  0  0
protocols.relax.FastRelax: CMD: accept_to_best  -933.45  0  0  0.55
protocols.relax.FastRelax: CMD: endrepeat  -933.45  0  0  0.55
protocols::checkpoint: Deleting checkpoints of FastRelax
```

Out[30]:

```
True
```

In [31]:

```
rstacked_G17 = pose_from_pdb('pdb/G17_stacked.relax.pdb')
sfxn.show(rstacked_G17)
```

```
core.import_pose.import_pose: File 'pdb/G17_stacked.relax.pdb' automatically determined to be of type PDB
core.scoring.ScoreFunction: 
------------------------------------------------------------
 Scores                       Weight   Raw Score Wghtd.Score
------------------------------------------------------------
 fa_atr                       1.000   -9561.577   -9561.577
 fa_rep                       0.550    3371.184    1854.151
 fa_sol                       1.000    8555.269    8555.269
 fa_intra_rep                 0.005    5408.344      27.042
 fa_intra_sol_xover4          1.000    1059.993    1059.993
 lk_ball_wtd                  1.000    -537.733    -537.733
 fa_elec                      1.000   -2450.925   -2450.925
 pro_close                    1.250       2.259       2.824
 hbond_sr_bb                  1.000    -443.323    -443.323
 hbond_lr_bb                  1.000     -41.373     -41.373
 hbond_bb_sc                  1.000     -65.995     -65.995
 hbond_sc                     1.000    -625.453    -625.453
 dslf_fa13                    1.250       0.000       0.000
 omega                        0.400     181.513      72.605
 fa_dun                       0.700    1497.123    1047.986
 p_aa_pp                      0.600    -130.171     -78.103
 yhh_planarity                0.625       0.000       0.000
 ref                          1.000     272.338     272.338
 rama_prepro                  0.450     -52.651     -23.693
---------------------------------------------------
 Total weighted score:                     -935.967
```

In [32]:

```
print(rstacked_G17.pdb_info())
```

```
PDB file name: pdb/G17_stacked.relax.pdb
 Pose Range  Chain    PDB Range  |   #Residues         #Atoms

0001 -- 0067    A 0001  -- 0067  |   0067 residues;    01076 atoms
0068 -- 0134    A 0001  -- 0067  |   0067 residues;    01076 atoms
0135 -- 0202    B 0001  -- 0068  |   0068 residues;    01098 atoms
0203 -- 0270    B 0001  -- 0068  |   0068 residues;    01098 atoms
0271 -- 0338    C 0001  -- 0068  |   0068 residues;    01098 atoms
0339 -- 0406    C 0001  -- 0068  |   0068 residues;    01098 atoms
0407 -- 0473    D 0001  -- 0067  |   0067 residues;    01076 atoms
0474 -- 0540    D 0001  -- 0067  |   0067 residues;    01076 atoms
0541 -- 0607    E 0001  -- 0067  |   0067 residues;    01076 atoms
0608 -- 0674    E 0001  -- 0067  |   0067 residues;    01076 atoms
0675 -- 0741    F 0001  -- 0067  |   0067 residues;    01076 atoms
0742 -- 0808    F 0001  -- 0067  |   0067 residues;    01076 atoms
0809 -- 0898    I 0001  -- 0090  |   0090 residues;    02859 atoms
0899 -- 0988    I 0001  -- 0090  |   0090 residues;    02859 atoms
0989 -- 1078    J 0001  -- 0090  |   0090 residues;    02862 atoms
1079 -- 1168    J 0001  -- 0090  |   0090 residues;    02862 atoms
                           TOTAL |   1168 residues;    24442 atoms
```

In [33]:

```
temp_G17 = rstacked_G17.clone()
```

In [34]:

```
mutate_residue(temp_G17, 16, "D")
sfxn.show(temp_G17)
```

```
core.scoring.ScoreFunctionFactory: SCOREFUNCTION: ref2015
core.pack.task: Packer task: initialize from command line()
core.pack.pack_rotamers: built 1 rotamers at 1 positions.
core.pack.interaction_graph.interaction_graph_factory: Instantiating PDInteractionGraph
core.scoring.ScoreFunction: 
------------------------------------------------------------
 Scores                       Weight   Raw Score Wghtd.Score
------------------------------------------------------------
 fa_atr                       1.000   -9568.734   -9568.734
 fa_rep                       0.550    3797.041    2088.373
 fa_sol                       1.000    8565.041    8565.041
 fa_intra_rep                 0.005    5409.612      27.048
 fa_intra_sol_xover4          1.000    1060.959    1060.959
 lk_ball_wtd                  1.000    -536.794    -536.794
 fa_elec                      1.000   -2445.773   -2445.773
 pro_close                    1.250       2.259       2.824
 hbond_sr_bb                  1.000    -443.323    -443.323
 hbond_lr_bb                  1.000     -41.373     -41.373
 hbond_bb_sc                  1.000     -65.995     -65.995
 hbond_sc                     1.000    -625.453    -625.453
 dslf_fa13                    1.250       0.000       0.000
 omega                        0.400     181.542      72.617
 fa_dun                       0.700    1502.730    1051.911
 p_aa_pp                      0.600    -124.317     -74.590
 yhh_planarity                0.625       0.000       0.000
 ref                          1.000     269.395     269.395
 rama_prepro                  0.450     -37.585     -16.913
---------------------------------------------------
 Total weighted score:                     -680.781
```

In [35]:

```
mutate_residue(temp_G17, 218, "D")
sfxn.show(temp_G17)
```

```
core.scoring.ScoreFunctionFactory: SCOREFUNCTION: ref2015
core.pack.task: Packer task: initialize from command line()
core.pack.pack_rotamers: built 1 rotamers at 1 positions.
core.pack.interaction_graph.interaction_graph_factory: Instantiating PDInteractionGraph
core.scoring.ScoreFunction: 
------------------------------------------------------------
 Scores                       Weight   Raw Score Wghtd.Score
------------------------------------------------------------
 fa_atr                       1.000   -9576.305   -9576.305
 fa_rep                       0.550    4204.456    2312.451
 fa_sol                       1.000    8575.128    8575.128
 fa_intra_rep                 0.005    5410.856      27.054
 fa_intra_sol_xover4          1.000    1061.921    1061.921
 lk_ball_wtd                  1.000    -535.422    -535.422
 fa_elec                      1.000   -2443.559   -2443.559
 pro_close                    1.250       2.259       2.824
 hbond_sr_bb                  1.000    -443.323    -443.323
 hbond_lr_bb                  1.000     -41.373     -41.373
 hbond_bb_sc                  1.000     -65.995     -65.995
 hbond_sc                     1.000    -625.908    -625.908
 dslf_fa13                    1.250       0.000       0.000
 omega                        0.400     181.617      72.647
 fa_dun                       0.700    1508.359    1055.851
 p_aa_pp                      0.600    -118.455     -71.073
 yhh_planarity                0.625       0.000       0.000
 ref                          1.000     266.451     266.451
 rama_prepro                  0.450     -22.609     -10.174
---------------------------------------------------
 Total weighted score:                     -438.807
```

In [36]:

```
movemap = MoveMap()
movemap.set_bb(False)
movemap.set_chi(True)
relax = pyrosetta.rosetta.protocols.relax.FastRelax()
relax.constrain_relax_to_start_coords(True)
relax.coord_constrain_sidechains(True)
relax.ramp_down_constraints(False)
relax.set_scorefxn(sfxn)
relax.set_movemap(movemap)
relax.apply(temp_G17)
temp_G17.dump_pdb('pdb/G17D_stacked.relax.pdb')
```

```
core.scoring.ScoreFunctionFactory: SCOREFUNCTION: ref2015
protocols.relax: turning off DNA bb and chi move
protocols.relax: turning off DNA bb and chi move
protocols.relax: turning off DNA bb and chi move
protocols.relax: turning off DNA bb and chi move
protocols.relax: turning off DNA bb and chi move
protocols.relax: turning off DNA bb and chi move
protocols.relax: turning off DNA bb and chi move
protocols.relax: turning off DNA bb and chi move
protocols.relax: turning off DNA bb and chi move
protocols.relax: turning off DNA bb and chi move
protocols.relax: turning off DNA bb and chi move
protocols.relax: turning off DNA bb and chi move
protocols.relax: turning off DNA bb and chi move
protocols.relax: turning off DNA bb and chi move
protocols.relax: turning off DNA bb and chi move
protocols.relax: turning off DNA bb and chi move
protocols.relax: turning off DNA bb and chi move
protocols.relax: turning off DNA bb and chi move
protocols.relax: turning off DNA bb and chi move
protocols.relax: turning off DNA bb and chi move
protocols.relax: turning off DNA bb and chi move
protocols.relax: turning off DNA bb and chi move
protocols.relax: turning off DNA bb and chi move
protocols.relax: turning off DNA bb and chi move
protocols.relax: turning off DNA bb and chi move
protocols.relax: turning off DNA bb and chi move
protocols.relax: turning off DNA bb and chi move
protocols.relax: turning off DNA bb and chi move
protocols.relax: turning off DNA bb and chi move
protocols.relax: turning off DNA bb and chi move
protocols.relax: turning off DNA bb and chi move
protocols.relax: turning off DNA bb and chi move
protocols.relax: turning off DNA bb and chi move
protocols.relax: turning off DNA bb and chi move
protocols.relax: turning off DNA bb and chi move
protocols.relax: turning off DNA bb and chi move
protocols.relax: turning off DNA bb and chi move
protocols.relax: turning off DNA bb and chi move
protocols.relax: turning off DNA bb and chi move
protocols.relax: turning off DNA bb and chi move
protocols.relax: turning off DNA bb and chi move
protocols.relax: turning off DNA bb and chi move
protocols.relax: turning off DNA bb and chi move
protocols.relax: turning off DNA bb and chi move
protocols.relax: turning off DNA bb and chi move
protocols.relax: turning off DNA bb and chi move
protocols.relax: turning off DNA bb and chi move
protocols.relax: turning off DNA bb and chi move
protocols.relax: turning off DNA bb and chi move
protocols.relax: turning off DNA bb and chi move
protocols.relax: turning off DNA bb and chi move
protocols.relax: turning off DNA bb and chi move
protocols.relax: turning off DNA bb and chi move
protocols.relax: turning off DNA bb and chi move
protocols.relax: turning off DNA bb and chi move
protocols.relax: turning off DNA bb and chi move
protocols.relax: turning off DNA bb and chi move
protocols.relax: turning off DNA bb and chi move
protocols.relax: turning off DNA bb and chi move
protocols.relax: turning off DNA bb and chi move
protocols.relax: turning off DNA bb and chi move
protocols.relax: turning off DNA bb and chi move
protocols.relax: turning off DNA bb and chi move
protocols.relax: turning off DNA bb and chi move
protocols.relax: turning off DNA bb and chi move
protocols.relax: turning off DNA bb and chi move
protocols.relax: turning off DNA bb and chi move
protocols.relax: turning off DNA bb and chi move
protocols.relax: turning off DNA bb and chi move
protocols.relax: turning off DNA bb and chi move
protocols.relax: turning off DNA bb and chi move
protocols.relax: turning off DNA bb and chi move
protocols.relax: turning off DNA bb and chi move
protocols.relax: turning off DNA bb and chi move
protocols.relax: turning off DNA bb and chi move
protocols.relax: turning off DNA bb and chi move
protocols.relax: turning off DNA bb and chi move
protocols.relax: turning off DNA bb and chi move
protocols.relax: turning off DNA bb and chi move
protocols.relax: turning off DNA bb and chi move
protocols.relax: turning off DNA bb and chi move
protocols.relax: turning off DNA bb and chi move
protocols.relax: turning off DNA bb and chi move
protocols.relax: turning off DNA bb and chi move
protocols.relax: turning off DNA bb and chi move
protocols.relax: turning off DNA bb and chi move
protocols.relax: turning off DNA bb and chi move
protocols.relax: turning off DNA bb and chi move
protocols.relax: turning off DNA bb and chi move
protocols.relax: turning off DNA bb and chi move
protocols.relax: turning off DNA bb and chi move
protocols.relax: turning off DNA bb and chi move
protocols.relax: turning off DNA bb and chi move
protocols.relax: turning off DNA bb and chi move
protocols.relax: turning off DNA bb and chi move
protocols.relax: turning off DNA bb and chi move
protocols.relax: turning off DNA bb and chi move
protocols.relax: turning off DNA bb and chi move
protocols.relax: turning off DNA bb and chi move
protocols.relax: turning off DNA bb and chi move
protocols.relax: turning off DNA bb and chi move
protocols.relax: turning off DNA bb and chi move
protocols.relax: turning off DNA bb and chi move
protocols.relax: turning off DNA bb and chi move
protocols.relax: turning off DNA bb and chi move
protocols.relax: turning off DNA bb and chi move
protocols.relax: turning off DNA bb and chi move
protocols.relax: turning off DNA bb and chi move
protocols.relax: turning off DNA bb and chi move
protocols.relax: turning off DNA bb and chi move
protocols.relax: turning off DNA bb and chi move
protocols.relax: turning off DNA bb and chi move
protocols.relax: turning off DNA bb and chi move
protocols.relax: turning off DNA bb and chi move
protocols.relax: turning off DNA bb and chi move
protocols.relax: turning off DNA bb and chi move
protocols.relax: turning off DNA bb and chi move
protocols.relax: turning off DNA bb and chi move
protocols.relax: turning off DNA bb and chi move
protocols.relax: turning off DNA bb and chi move
protocols.relax: turning off DNA bb and chi move
protocols.relax: turning off DNA bb and chi move
protocols.relax: turning off DNA bb and chi move
protocols.relax: turning off DNA bb and chi move
protocols.relax: turning off DNA bb and chi move
protocols.relax: turning off DNA bb and chi move
protocols.relax: turning off DNA bb and chi move
protocols.relax: turning off DNA bb and chi move
protocols.relax: turning off DNA bb and chi move
protocols.relax: turning off DNA bb and chi move
protocols.relax: turning off DNA bb and chi move
protocols.relax: turning off DNA bb and chi move
protocols.relax: turning off DNA bb and chi move
protocols.relax: turning off DNA bb and chi move
protocols.relax: turning off DNA bb and chi move
protocols.relax: turning off DNA bb and chi move
protocols.relax: turning off DNA bb and chi move
protocols.relax: turning off DNA bb and chi move
protocols.relax: turning off DNA bb and chi move
protocols.relax: turning off DNA bb and chi move
protocols.relax: turning off DNA bb and chi move
protocols.relax: turning off DNA bb and chi move
protocols.relax: turning off DNA bb and chi move
protocols.relax: turning off DNA bb and chi move
protocols.relax: turning off DNA bb and chi move
protocols.relax: turning off DNA bb and chi move
protocols.relax: turning off DNA bb and chi move
protocols.relax: turning off DNA bb and chi move
protocols.relax: turning off DNA bb and chi move
protocols.relax: turning off DNA bb and chi move
protocols.relax: turning off DNA bb and chi move
protocols.relax: turning off DNA bb and chi move
protocols.relax: turning off DNA bb and chi move
protocols.relax: turning off DNA bb and chi move
protocols.relax: turning off DNA bb and chi move
protocols.relax: turning off DNA bb and chi move
protocols.relax: turning off DNA bb and chi move
protocols.relax: turning off DNA bb and chi move
protocols.relax: turning off DNA bb and chi move
protocols.relax: turning off DNA bb and chi move
protocols.relax: turning off DNA bb and chi move
protocols.relax: turning off DNA bb and chi move
protocols.relax: turning off DNA bb and chi move
protocols.relax: turning off DNA bb and chi move
protocols.relax: turning off DNA bb and chi move
protocols.relax: turning off DNA bb and chi move
protocols.relax: turning off DNA bb and chi move
protocols.relax: turning off DNA bb and chi move
protocols.relax: turning off DNA bb and chi move
protocols.relax: turning off DNA bb and chi move
protocols.relax: turning off DNA bb and chi move
protocols.relax: turning off DNA bb and chi move
protocols.relax: turning off DNA bb and chi move
protocols.relax: turning off DNA bb and chi move
protocols.relax: turning off DNA bb and chi move
protocols.relax: turning off DNA bb and chi move
protocols.relax: turning off DNA bb and chi move
protocols.relax: turning off DNA bb and chi move
protocols.relax: turning off DNA bb and chi move
protocols.relax: turning off DNA bb and chi move
protocols.relax: turning off DNA bb and chi move
protocols.relax: turning off DNA bb and chi move
protocols.relax: turning off DNA bb and chi move
protocols.relax: turning off DNA bb and chi move
protocols.relax: turning off DNA bb and chi move
protocols.relax: turning off DNA bb and chi move
protocols.relax: turning off DNA bb and chi move
protocols.relax: turning off DNA bb and chi move
protocols.relax: turning off DNA bb and chi move
protocols.relax: turning off DNA bb and chi move
protocols.relax: turning off DNA bb and chi move
protocols.relax: turning off DNA bb and chi move
protocols.relax: turning off DNA bb and chi move
protocols.relax: turning off DNA bb and chi move
protocols.relax: turning off DNA bb and chi move
protocols.relax: turning off DNA bb and chi move
protocols.relax: turning off DNA bb and chi move
protocols.relax: turning off DNA bb and chi move
protocols.relax: turning off DNA bb and chi move
protocols.relax: turning off DNA bb and chi move
protocols.relax: turning off DNA bb and chi move
protocols.relax: turning off DNA bb and chi move
protocols.relax: turning off DNA bb and chi move
protocols.relax: turning off DNA bb and chi move
protocols.relax: turning off DNA bb and chi move
protocols.relax: turning off DNA bb and chi move
protocols.relax: turning off DNA bb and chi move
protocols.relax: turning off DNA bb and chi move
protocols.relax: turning off DNA bb and chi move
protocols.relax: turning off DNA bb and chi move
protocols.relax: turning off DNA bb and chi move
protocols.relax: turning off DNA bb and chi move
protocols.relax: turning off DNA bb and chi move
protocols.relax: turning off DNA bb and chi move
protocols.relax: turning off DNA bb and chi move
protocols.relax: turning off DNA bb and chi move
protocols.relax: turning off DNA bb and chi move
protocols.relax: turning off DNA bb and chi move
protocols.relax: turning off DNA bb and chi move
protocols.relax: turning off DNA bb and chi move
protocols.relax: turning off DNA bb and chi move
protocols.relax: turning off DNA bb and chi move
protocols.relax: turning off DNA bb and chi move
protocols.relax: turning off DNA bb and chi move
protocols.relax: turning off DNA bb and chi move
protocols.relax: turning off DNA bb and chi move
protocols.relax: turning off DNA bb and chi move
protocols.relax: turning off DNA bb and chi move
protocols.relax: turning off DNA bb and chi move
protocols.relax: turning off DNA bb and chi move
protocols.relax: turning off DNA bb and chi move
protocols.relax: turning off DNA bb and chi move
protocols.relax: turning off DNA bb and chi move
protocols.relax: turning off DNA bb and chi move
protocols.relax: turning off DNA bb and chi move
protocols.relax: turning off DNA bb and chi move
protocols.relax: turning off DNA bb and chi move
protocols.relax: turning off DNA bb and chi move
protocols.relax: turning off DNA bb and chi move
protocols.relax: turning off DNA bb and chi move
protocols.relax: turning off DNA bb and chi move
protocols.relax: turning off DNA bb and chi move
protocols.relax: turning off DNA bb and chi move
protocols.relax: turning off DNA bb and chi move
protocols.relax: turning off DNA bb and chi move
protocols.relax: turning off DNA bb and chi move
protocols.relax: turning off DNA bb and chi move
protocols.relax: turning off DNA bb and chi move
protocols.relax: turning off DNA bb and chi move
protocols.relax: turning off DNA bb and chi move
protocols.relax: turning off DNA bb and chi move
protocols.relax: turning off DNA bb and chi move
protocols.relax: turning off DNA bb and chi move
protocols.relax: turning off DNA bb and chi move
protocols.relax: turning off DNA bb and chi move
protocols.relax: turning off DNA bb and chi move
protocols.relax: turning off DNA bb and chi move
protocols.relax: turning off DNA bb and chi move
protocols.relax: turning off DNA bb and chi move
protocols.relax: turning off DNA bb and chi move
protocols.relax: turning off DNA bb and chi move
protocols.relax: turning off DNA bb and chi move
protocols.relax: turning off DNA bb and chi move
protocols.relax: turning off DNA bb and chi move
protocols.relax: turning off DNA bb and chi move
protocols.relax: turning off DNA bb and chi move
protocols.relax: turning off DNA bb and chi move
protocols.relax: turning off DNA bb and chi move
protocols.relax: turning off DNA bb and chi move
protocols.relax: turning off DNA bb and chi move
protocols.relax: turning off DNA bb and chi move
protocols.relax: turning off DNA bb and chi move
protocols.relax: turning off DNA bb and chi move
protocols.relax: turning off DNA bb and chi move
protocols.relax: turning off DNA bb and chi move
protocols.relax: turning off DNA bb and chi move
protocols.relax: turning off DNA bb and chi move
protocols.relax: turning off DNA bb and chi move
protocols.relax: turning off DNA bb and chi move
protocols.relax: turning off DNA bb and chi move
protocols.relax: turning off DNA bb and chi move
protocols.relax: turning off DNA bb and chi move
protocols.relax: turning off DNA bb and chi move
protocols.relax: turning off DNA bb and chi move
protocols.relax: turning off DNA bb and chi move
protocols.relax: turning off DNA bb and chi move
protocols.relax: turning off DNA bb and chi move
protocols.relax: turning off DNA bb and chi move
protocols.relax: turning off DNA bb and chi move
protocols.relax: turning off DNA bb and chi move
protocols.relax: turning off DNA bb and chi move
protocols.relax: turning off DNA bb and chi move
protocols.relax: turning off DNA bb and chi move
protocols.relax: turning off DNA bb and chi move
protocols.relax: turning off DNA bb and chi move
protocols.relax: turning off DNA bb and chi move
protocols.relax: turning off DNA bb and chi move
protocols.relax: turning off DNA bb and chi move
protocols.relax: turning off DNA bb and chi move
protocols.relax: turning off DNA bb and chi move
protocols.relax: turning off DNA bb and chi move
protocols.relax: turning off DNA bb and chi move
protocols.relax: turning off DNA bb and chi move
protocols.relax: turning off DNA bb and chi move
protocols.relax: turning off DNA bb and chi move
protocols.relax: turning off DNA bb and chi move
protocols.relax: turning off DNA bb and chi move
protocols.relax: turning off DNA bb and chi move
protocols.relax: turning off DNA bb and chi move
protocols.relax: turning off DNA bb and chi move
protocols.relax: turning off DNA bb and chi move
protocols.relax: turning off DNA bb and chi move
protocols.relax: turning off DNA bb and chi move
protocols.relax: turning off DNA bb and chi move
protocols.relax: turning off DNA bb and chi move
protocols.relax: turning off DNA bb and chi move
protocols.relax: turning off DNA bb and chi move
protocols.relax: turning off DNA bb and chi move
protocols.relax: turning off DNA bb and chi move
protocols.relax: turning off DNA bb and chi move
protocols.relax: turning off DNA bb and chi move
protocols.relax: turning off DNA bb and chi move
protocols.relax: turning off DNA bb and chi move
protocols.relax: turning off DNA bb and chi move
protocols.relax: turning off DNA bb and chi move
protocols.relax: turning off DNA bb and chi move
protocols.relax: turning off DNA bb and chi move
protocols.relax: turning off DNA bb and chi move
protocols.relax: turning off DNA bb and chi move
protocols.relax: turning off DNA bb and chi move
protocols.relax: turning off DNA bb and chi move
protocols.relax: turning off DNA bb and chi move
protocols.relax: turning off DNA bb and chi move
protocols.relax: turning off DNA bb and chi move
protocols.relax: turning off DNA bb and chi move
protocols.relax: turning off DNA bb and chi move
protocols.relax: turning off DNA bb and chi move
protocols.relax: turning off DNA bb and chi move
protocols.relax: turning off DNA bb and chi move
protocols.relax: turning off DNA bb and chi move
protocols.relax: turning off DNA bb and chi move
protocols.relax: turning off DNA bb and chi move
protocols.relax: turning off DNA bb and chi move
protocols.relax: turning off DNA bb and chi move
protocols.relax: turning off DNA bb and chi move
protocols.relax: turning off DNA bb and chi move
protocols.relax: turning off DNA bb and chi move
protocols.relax: turning off DNA bb and chi move
protocols.relax: turning off DNA bb and chi move
protocols.relax: turning off DNA bb and chi move
protocols.relax: turning off DNA bb and chi move
protocols.relax: turning off DNA bb and chi move
protocols.relax: turning off DNA bb and chi move
protocols.relax: turning off DNA bb and chi move
protocols.relax: turning off DNA bb and chi move
protocols.relax: turning off DNA bb and chi move
protocols.relax: turning off DNA bb and chi move
protocols.relax: turning off DNA bb and chi move
protocols.relax: turning off DNA bb and chi move
protocols.relax: turning off DNA bb and chi move
protocols.relax.FastRelax: CMD: repeat  -438.807  0  0  0.55
protocols.relax.FastRelax: CMD: coord_cst_weight  -438.807  0  0  0.55
protocols.relax.FastRelax: CMD: scale:fa_rep  -2658.76  0  0  0.022
core.pack.task: Packer task: initialize from command line()
core.pack.pack_rotamers: built 22179 rotamers at 1168 positions.
core.pack.interaction_graph.interaction_graph_factory: Instantiating DensePDInteractionGraph
protocols.relax.FastRelax: CMD: repack  -2829.43  0  0  0.022
protocols.relax.FastRelax: CMD: scale:fa_rep  -2784.95  0  0  0.02805
protocols.relax.FastRelax: CMD: min  -2785.03  0  0  0.02805
protocols.relax.FastRelax: CMD: coord_cst_weight  -2785.03  0  0  0.02805
protocols.relax.FastRelax: CMD: scale:fa_rep  -1919.8  0  0  0.14575
core.pack.task: Packer task: initialize from command line()
core.pack.pack_rotamers: built 19756 rotamers at 1168 positions.
core.pack.interaction_graph.interaction_graph_factory: Instantiating DensePDInteractionGraph
protocols.relax.FastRelax: CMD: repack  -2148.02  0  0  0.14575
protocols.relax.FastRelax: CMD: scale:fa_rep  -2108.56  0  0  0.154
protocols.relax.FastRelax: CMD: min  -2108.73  0  0  0.154
protocols.relax.FastRelax: CMD: coord_cst_weight  -2108.73  0  0  0.154
protocols.relax.FastRelax: CMD: scale:fa_rep  -1374.89  0  0  0.30745
core.pack.task: Packer task: initialize from command line()
core.pack.pack_rotamers: built 18692 rotamers at 1168 positions.
core.pack.interaction_graph.interaction_graph_factory: Instantiating DensePDInteractionGraph
protocols.relax.FastRelax: CMD: repack  -1401.33  0  0  0.30745
protocols.relax.FastRelax: CMD: scale:fa_rep  -1346.53  0  0  0.31955
protocols.relax.FastRelax: CMD: min  -1446.82  0  0  0.31955
protocols.relax.FastRelax: CMD: coord_cst_weight  -1446.82  0  0  0.31955
protocols.relax.FastRelax: CMD: scale:fa_rep  -482.7  0  0  0.55
core.pack.task: Packer task: initialize from command line()
core.pack.pack_rotamers: built 17936 rotamers at 1168 positions.
core.pack.interaction_graph.interaction_graph_factory: Instantiating DensePDInteractionGraph
protocols.relax.FastRelax: CMD: repack  -459.373  0  0  0.55
protocols.relax.FastRelax: CMD: min  -628.071  0  0  0.55
protocols.relax.FastRelax: MRP: 0  -628.071  -628.071  0  0
protocols.relax.FastRelax: CMD: accept_to_best  -628.071  0  0  0.55
protocols.relax.FastRelax: CMD: endrepeat  -628.071  0  0  0.55
protocols.relax.FastRelax: CMD: coord_cst_weight  -628.071  0  0  0.55
protocols.relax.FastRelax: CMD: scale:fa_rep  -2644.2  0  0  0.022
core.pack.task: Packer task: initialize from command line()
core.pack.pack_rotamers: built 22179 rotamers at 1168 positions.
core.pack.interaction_graph.interaction_graph_factory: Instantiating DensePDInteractionGraph
protocols.relax.FastRelax: CMD: repack  -2837.98  0  0  0.022
protocols.relax.FastRelax: CMD: scale:fa_rep  -2793.58  0  0  0.02805
protocols.relax.FastRelax: CMD: min  -2793.65  0  0  0.02805
protocols.relax.FastRelax: CMD: coord_cst_weight  -2793.65  0  0  0.02805
protocols.relax.FastRelax: CMD: scale:fa_rep  -1929.82  0  0  0.14575
core.pack.task: Packer task: initialize from command line()
core.pack.pack_rotamers: built 19756 rotamers at 1168 positions.
core.pack.interaction_graph.interaction_graph_factory: Instantiating DensePDInteractionGraph
protocols.relax.FastRelax: CMD: repack  -2154.35  0  0  0.14575
protocols.relax.FastRelax: CMD: scale:fa_rep  -2114.51  0  0  0.154
protocols.relax.FastRelax: CMD: min  -2114.71  0  0  0.154
protocols.relax.FastRelax: CMD: coord_cst_weight  -2114.71  0  0  0.154
protocols.relax.FastRelax: CMD: scale:fa_rep  -1373.83  0  0  0.30745
core.pack.task: Packer task: initialize from command line()
core.pack.pack_rotamers: built 18692 rotamers at 1168 positions.
core.pack.interaction_graph.interaction_graph_factory: Instantiating DensePDInteractionGraph
protocols.relax.FastRelax: CMD: repack  -1405.06  0  0  0.30745
protocols.relax.FastRelax: CMD: scale:fa_rep  -1349.99  0  0  0.31955
protocols.relax.FastRelax: CMD: min  -1449.64  0  0  0.31955
protocols.relax.FastRelax: CMD: coord_cst_weight  -1449.64  0  0  0.31955
protocols.relax.FastRelax: CMD: scale:fa_rep  -481.351  0  0  0.55
core.pack.task: Packer task: initialize from command line()
core.pack.pack_rotamers: built 17936 rotamers at 1168 positions.
core.pack.interaction_graph.interaction_graph_factory: Instantiating DensePDInteractionGraph
protocols.relax.FastRelax: CMD: repack  -484.856  0  0  0.55
protocols.relax.FastRelax: CMD: min  -637.75  0  0  0.55
protocols.relax.FastRelax: MRP: 1  -637.75  -637.75  0  0
protocols.relax.FastRelax: CMD: accept_to_best  -637.75  0  0  0.55
protocols.relax.FastRelax: CMD: endrepeat  -637.75  0  0  0.55
protocols.relax.FastRelax: CMD: coord_cst_weight  -637.75  0  0  0.55
protocols.relax.FastRelax: CMD: scale:fa_rep  -2642.27  0  0  0.022
core.pack.task: Packer task: initialize from command line()
core.pack.pack_rotamers: built 22179 rotamers at 1168 positions.
core.pack.interaction_graph.interaction_graph_factory: Instantiating DensePDInteractionGraph
protocols.relax.FastRelax: CMD: repack  -2836.99  0  0  0.022
protocols.relax.FastRelax: CMD: scale:fa_rep  -2791.28  0  0  0.02805
protocols.relax.FastRelax: CMD: min  -2791.37  0  0  0.02805
protocols.relax.FastRelax: CMD: coord_cst_weight  -2791.37  0  0  0.02805
protocols.relax.FastRelax: CMD: scale:fa_rep  -1902.15  0  0  0.14575
core.pack.task: Packer task: initialize from command line()
core.pack.pack_rotamers: built 19756 rotamers at 1168 positions.
core.pack.interaction_graph.interaction_graph_factory: Instantiating DensePDInteractionGraph
protocols.relax.FastRelax: CMD: repack  -2148.96  0  0  0.14575
protocols.relax.FastRelax: CMD: scale:fa_rep  -2108.76  0  0  0.154
protocols.relax.FastRelax: CMD: min  -2108.94  0  0  0.154
protocols.relax.FastRelax: CMD: coord_cst_weight  -2108.94  0  0  0.154
protocols.relax.FastRelax: CMD: scale:fa_rep  -1361.36  0  0  0.30745
core.pack.task: Packer task: initialize from command line()
core.pack.pack_rotamers: built 18692 rotamers at 1168 positions.
core.pack.interaction_graph.interaction_graph_factory: Instantiating DensePDInteractionGraph
protocols.relax.FastRelax: CMD: repack  -1392.51  0  0  0.30745
protocols.relax.FastRelax: CMD: scale:fa_rep  -1337.02  0  0  0.31955
protocols.relax.FastRelax: CMD: min  -1451.42  0  0  0.31955
protocols.relax.FastRelax: CMD: coord_cst_weight  -1451.42  0  0  0.31955
protocols.relax.FastRelax: CMD: scale:fa_rep  -486.622  0  0  0.55
core.pack.task: Packer task: initialize from command line()
core.pack.pack_rotamers: built 17936 rotamers at 1168 positions.
core.pack.interaction_graph.interaction_graph_factory: Instantiating DensePDInteractionGraph
protocols.relax.FastRelax: CMD: repack  -488.624  0  0  0.55
protocols.relax.FastRelax: CMD: min  -645.606  0  0  0.55
protocols.relax.FastRelax: MRP: 2  -645.606  -645.606  0  0
protocols.relax.FastRelax: CMD: accept_to_best  -645.606  0  0  0.55
protocols.relax.FastRelax: CMD: endrepeat  -645.606  0  0  0.55
protocols.relax.FastRelax: CMD: coord_cst_weight  -645.606  0  0  0.55
protocols.relax.FastRelax: CMD: scale:fa_rep  -2650.89  0  0  0.022
core.pack.task: Packer task: initialize from command line()
core.pack.pack_rotamers: built 22179 rotamers at 1168 positions.
core.pack.interaction_graph.interaction_graph_factory: Instantiating DensePDInteractionGraph
protocols.relax.FastRelax: CMD: repack  -2843.1  0  0  0.022
protocols.relax.FastRelax: CMD: scale:fa_rep  -2798.06  0  0  0.02805
protocols.relax.FastRelax: CMD: min  -2798.18  0  0  0.02805
protocols.relax.FastRelax: CMD: coord_cst_weight  -2798.18  0  0  0.02805
protocols.relax.FastRelax: CMD: scale:fa_rep  -1922.16  0  0  0.14575
core.pack.task: Packer task: initialize from command line()
core.pack.pack_rotamers: built 19756 rotamers at 1168 positions.
core.pack.interaction_graph.interaction_graph_factory: Instantiating DensePDInteractionGraph
protocols.relax.FastRelax: CMD: repack  -2158.25  0  0  0.14575
protocols.relax.FastRelax: CMD: scale:fa_rep  -2118.84  0  0  0.154
protocols.relax.FastRelax: CMD: min  -2119.01  0  0  0.154
protocols.relax.FastRelax: CMD: coord_cst_weight  -2119.01  0  0  0.154
protocols.relax.FastRelax: CMD: scale:fa_rep  -1385.95  0  0  0.30745
core.pack.task: Packer task: initialize from command line()
core.pack.pack_rotamers: built 18692 rotamers at 1168 positions.
core.pack.interaction_graph.interaction_graph_factory: Instantiating DensePDInteractionGraph
protocols.relax.FastRelax: CMD: repack  -1415.76  0  0  0.30745
protocols.relax.FastRelax: CMD: scale:fa_rep  -1361.05  0  0  0.31955
protocols.relax.FastRelax: CMD: min  -1457.4  0  0  0.31955
protocols.relax.FastRelax: CMD: coord_cst_weight  -1457.4  0  0  0.31955
protocols.relax.FastRelax: CMD: scale:fa_rep  -494.699  0  0  0.55
core.pack.task: Packer task: initialize from command line()
core.pack.pack_rotamers: built 17936 rotamers at 1168 positions.
core.pack.interaction_graph.interaction_graph_factory: Instantiating DensePDInteractionGraph
protocols.relax.FastRelax: CMD: repack  -497.318  0  0  0.55
protocols.relax.FastRelax: CMD: min  -642.637  0  0  0.55
protocols.relax.FastRelax: MRP: 3  -642.637  -645.606  0  0
protocols.relax.FastRelax: CMD: accept_to_best  -642.637  0  0  0.55
protocols.relax.FastRelax: CMD: endrepeat  -642.637  0  0  0.55
protocols.relax.FastRelax: CMD: coord_cst_weight  -642.637  0  0  0.55
protocols.relax.FastRelax: CMD: scale:fa_rep  -2651.95  0  0  0.022
core.pack.task: Packer task: initialize from command line()
core.pack.pack_rotamers: built 22179 rotamers at 1168 positions.
core.pack.interaction_graph.interaction_graph_factory: Instantiating DensePDInteractionGraph
protocols.relax.FastRelax: CMD: repack  -2838.22  0  0  0.022
protocols.relax.FastRelax: CMD: scale:fa_rep  -2795.31  0  0  0.02805
protocols.relax.FastRelax: CMD: min  -2795.38  0  0  0.02805
protocols.relax.FastRelax: CMD: coord_cst_weight  -2795.38  0  0  0.02805
protocols.relax.FastRelax: CMD: scale:fa_rep  -1960.74  0  0  0.14575
core.pack.task: Packer task: initialize from command line()
core.pack.pack_rotamers: built 19756 rotamers at 1168 positions.
core.pack.interaction_graph.interaction_graph_factory: Instantiating DensePDInteractionGraph
protocols.relax.FastRelax: CMD: repack  -2152.88  0  0  0.14575
protocols.relax.FastRelax: CMD: scale:fa_rep  -2112.95  0  0  0.154
protocols.relax.FastRelax: CMD: min  -2113.15  0  0  0.154
protocols.relax.FastRelax: CMD: coord_cst_weight  -2113.15  0  0  0.154
protocols.relax.FastRelax: CMD: scale:fa_rep  -1370.6  0  0  0.30745
core.pack.task: Packer task: initialize from command line()
core.pack.pack_rotamers: built 18692 rotamers at 1168 positions.
core.pack.interaction_graph.interaction_graph_factory: Instantiating DensePDInteractionGraph
protocols.relax.FastRelax: CMD: repack  -1413.44  0  0  0.30745
protocols.relax.FastRelax: CMD: scale:fa_rep  -1358.59  0  0  0.31955
protocols.relax.FastRelax: CMD: min  -1456.06  0  0  0.31955
protocols.relax.FastRelax: CMD: coord_cst_weight  -1456.06  0  0  0.31955
protocols.relax.FastRelax: CMD: scale:fa_rep  -491.274  0  0  0.55
core.pack.task: Packer task: initialize from command line()
core.pack.pack_rotamers: built 17936 rotamers at 1168 positions.
core.pack.interaction_graph.interaction_graph_factory: Instantiating DensePDInteractionGraph
protocols.relax.FastRelax: CMD: repack  -489.677  0  0  0.55
protocols.relax.FastRelax: CMD: min  -636.663  0  0  0.55
protocols.relax.FastRelax: MRP: 4  -636.663  -645.606  0  0
protocols.relax.FastRelax: CMD: accept_to_best  -636.663  0  0  0.55
protocols.relax.FastRelax: CMD: endrepeat  -636.663  0  0  0.55
protocols::checkpoint: Deleting checkpoints of FastRelax
```

Out[36]:

```
True
```

In [37]:

```
rstacked_G17D = pose_from_pdb('pdb/G17D_stacked.relax.pdb')
sfxn.show(rstacked_G17D)
```

```
core.import_pose.import_pose: File 'pdb/G17D_stacked.relax.pdb' automatically determined to be of type PDB
core.scoring.ScoreFunction: 
------------------------------------------------------------
 Scores                       Weight   Raw Score Wghtd.Score
------------------------------------------------------------
 fa_atr                       1.000   -9578.724   -9578.724
 fa_rep                       0.550    3797.678    2088.723
 fa_sol                       1.000    8578.284    8578.284
 fa_intra_rep                 0.005    5464.246      27.321
 fa_intra_sol_xover4          1.000    1061.806    1061.806
 lk_ball_wtd                  1.000    -536.760    -536.760
 fa_elec                      1.000   -2455.973   -2455.973
 pro_close                    1.250       2.250       2.813
 hbond_sr_bb                  1.000    -443.323    -443.323
 hbond_lr_bb                  1.000     -41.373     -41.373
 hbond_bb_sc                  1.000     -66.260     -66.260
 hbond_sc                     1.000    -632.747    -632.747
 dslf_fa13                    1.250       0.000       0.000
 omega                        0.400     181.617      72.647
 fa_dun                       0.700    1560.750    1092.525
 p_aa_pp                      0.600    -118.455     -71.073
 yhh_planarity                0.625       0.000       0.000
 ref                          1.000     266.451     266.451
 rama_prepro                  0.450     -22.609     -10.174
---------------------------------------------------
 Total weighted score:                     -645.839
```

In [38]:

```
rstacked_G17 = pose_from_pdb('pdb/G17_stacked.relax.pdb')
```

```
core.import_pose.import_pose: File 'pdb/G17_stacked.relax.pdb' automatically determined to be of type PDB
```

In [39]:

```
pyrosetta.toolbox.atom_pair_energy.print_residue_pair_energies(16, rstacked_G17D, sfxn, scrtype2, 0)
```

```
D 14  0.24261654952235784
A 15  43.46926414282957
D 16  10264.501306433676
A 17  0.06608727665710067
I 178  15.574789369846055
D 216  147.16126128963919
A 217  3.5899765984593364
D 218  3.1852009274268447
```

In [40]:

```
pyrosetta.toolbox.atom_pair_energy.print_residue_pair_energies(16, rstacked_G17, sfxn, scrtype2, 0)
```

```
D 14  0.24261654952235784
A 15  0.131362490943214
G 16  5482.113821572886
A 17  0.06608727665710067
I 178  0.2962789704325796
D 216  0.22474737445681367
G 218  3.044020583987907
```

In [41]:

```
pyrosetta.toolbox.atom_pair_energy.print_residue_pair_energies(218, rstacked_G17D, sfxn, scrtype2, 0)
```

```
D 14  150.97012519464917
A 15  4.216614225608701
D 16  3.1852009274268447
I 111  9.993639295814766
R 115  0.0009543967015073485
R 171  0.07027511380632157
D 216  0.2781909437334993
A 217  37.95421042791955
D 218  10266.898712941062
A 219  0.07296458245374839
```

In [42]:

```
pyrosetta.toolbox.atom_pair_energy.print_residue_pair_energies(218, rstacked_G17, sfxn, scrtype2, 0)
```

```
D 14  0.25547238040991416
A 15  0.00023185675645213955
G 16  3.044020583987907
I 111  0.24340100223380645
R 115  0.005786710934754413
D 216  0.2781909437334993
A 217  0.13155673079127245
G 218  5484.700420631214
A 219  0.07296458245374839
```
