# Supplementary material for: Archaeal histone-based chromatin structures regulate transcription elongation rates
Source: Commun Biol. 2024 Feb 27;7:236. doi: 10.1038/s42003-024-05928-w (PMC10899632; doi:10.1038/s42003-024-05928-w)
Supplement: Supplementary file 6 — Reporting Summary [file 42003_2024_5928_MOESM6_ESM.pdf]

nature portfolio

Corresponding author(s): Thomas Santangelo

Last updated by author(s): Feb 13, 2024

Reporting Summary

Nature Portfolio wishes to improve the reproducibility of the work that we publish. This form provides structure for consistency and transparency in reporting. For further information on Nature Portfolio policies, see our Editorial Policies and the Editorial Policy Checklist.

Statistics

For all statistical analyses, confirm that the following items are present in the figure legend, table legend, main text, or Methods section.

n/a	Confirmed	
☐	☒	The exact sample size (*n*) for each experimental group/condition, given as a discrete number and unit of measurement
☐	☒	A statement on whether measurements were taken from distinct samples or whether the same sample was measured repeatedly
☒	☐	The statistical test(s) used AND whether they are one- or two-sided<br>*Only common tests should be described solely by name; describe more complex techniques in the Methods section.*
☒	☐	A description of all covariates tested
☐	☒	A description of any assumptions or corrections, such as tests of normality and adjustment for multiple comparisons
☐	☒	A full description of the statistical parameters including central tendency (e.g. means) or other basic estimates (e.g. regression coefficient) AND variation (e.g. standard deviation) or associated estimates of uncertainty (e.g. confidence intervals)
☒	☐	For null hypothesis testing, the test statistic (e.g. *F*, *t*, *r*) with confidence intervals, effect sizes, degrees of freedom and *P* value noted<br>*Give P values as exact values whenever suitable.*
☒	☐	For Bayesian analysis, information on the choice of priors and Markov chain Monte Carlo settings
☒	☐	For hierarchical and complex designs, identification of the appropriate level for tests and full reporting of outcomes
☐	☒	Estimates of effect sizes (e.g. Cohen's *d*, Pearson's *r*), indicating how they were calculated

Our web collection on statistics for biologists contains articles on many of the points above.

Software and code

Policy information about availability of computer code

Data collection	Typhoon FLA 9500 was used to image the radio-labeled gels. ImageQuant TL 8.2.0.0 was used to analyze phoshorimages. The TFS deletion strain was confirmed via WGS using our in-house MinION, which contains the sequencing software, MinKNOW. OriginPro 3D 2022 was used to create waterfall plots detailing the intensity and length of P32 radio-labeled RNA products from the ImageQuant TL 8.2.0.0 analysis.
Data analysis	Isotopically-labeled RNA species were quantified using GE ImageQuant TL 8.2.0.0 using established techniques common to the field. PyRosetta-4 was used to analyze the free energy of the wild type and mutant structures (a Jupyter Notebook containing the code for analysis has been submitted and is available on our GitHub repository: https://github.com/tjsantangelo). RStudio 2022.07.2+576 for macOS was used to analyze the lanes and relative intensity of the radio-labeled gels and to prepare figures. IGV genome browser (2.16.1) was used to visualize WGS of the TFS deletion strain. The mapped reads are available at BioProject accession number PRJNA996631 in the NCBI BioProject database.

For manuscripts utilizing custom algorithms or software that are central to the research but not yet described in published literature, software must be made available to editors and reviewers. We strongly encourage code deposition in a community repository (e.g. GitHub). See the Nature Portfolio guidelines for submitting code & software for further information.

Data

Policy information about availability of data

All manuscripts must include a data availability statement. This statement should provide the following information, where applicable:

- Accession codes, unique identifiers, or web links for publicly available datasets
- A description of any restrictions on data availability
- For clinical datasets or third party data, please ensure that the statement adheres to our policy

The mapped WGS reads along with the reference file are publicly available through links to BioProject accession number PRJNA996631 in the NCBI BioProject database (https://www.ncbi.nlm.nih.gov/bioproject/). The code to determine the free energy of the wild type and mutant histone-based chromatin structures is available on our GitHub repository: https://github.com/tjsantangelo. All raw images used for data analysis are available upon request. Representative images are included in the current manuscript.

Research involving human participants, their data, or biological material

Policy information about studies with human participants or human data. See also policy information about sex, gender (identity/presentation), and sexual orientation and race, ethnicity and racism.

Reporting on sex and gender	N/A
Reporting on race, ethnicity, or other socially relevant groupings	N/A
Population characteristics	N/A
Recruitment	N/A
Ethics oversight	N/A

Note that full information on the approval of the study protocol must also be provided in the manuscript.

Field-specific reporting

Please select the one below that is the best fit for your research. If you are not sure, read the appropriate sections before making your selection.

☒ Life sciences ☐ Behavioural & social sciences ☐ Ecological, evolutionary & environmental sciences

For a reference copy of the document with all sections, see nature.com/documents/nr-reporting-summary-flat.pdf

Life sciences study design

All studies must disclose on these points even when the disclosure is negative.

Sample size	Transcription elongation efficiencies were reported as the standard mean from at least three independent experiments. The exact number of independent replicates is reported for each figure. Experimentation was largely in vitro. Our in vivo work is reliant on strain constructions that we pioneered and all details are available in published literature.
Data exclusions	No data points were excluded from our analyses.
Replication	Figures and legends report means and standard deviations/errors demonstrating reproducibility of our experimentation.
Randomization	Randomization was not required for the current studies. In vitro biochemistry does not necessitate randomization.
Blinding	Blinding was not necessary for the current studies. In vitro biochemistry does not require blinding.

Reporting for specific materials, systems and methods

We require information from authors about some types of materials, experimental systems and methods used in many studies. Here, indicate whether each material, system or method listed is relevant to your study. If you are not sure if a list item applies to your research, read the appropriate section before selecting a response.

Materials & experimental systems

n/a	Involved in the study	
☐	☒	Antibodies
☒	☐	Eukaryotic cell lines
☒	☐	Palaeontology and archaeology
☐	☒	Animals and other organisms
☒	☐	Clinical data
☒	☐	Dual use research of concern
☒	☐	Plants

Methods

n/a	Involved in the study	
☒	☐	ChIP-seq
☒	☐	Flow cytometry
☒	☐	MRI-based neuroimaging

Antibodies

Antibodies used	Custom polyclonal anti-HTkA antibodies were generated in two rabbits from Cocalico Biologicals, Inc. (http://www.cocalicobiologicals.com; Stevens, PA) using full-length recombinant HTkA as an antigen. Total serum from each animal was provided and used in Western blots in the current work. Pre- and post-immunization serum was tested on purified, recombinant HTkA and extracts from WT cells. The effective dilutions of each lot of HTkA-antibodies are empirically determined in house, but typically range from 1:1,000 - 1:10,000.
Validation	Pre-immune serum and post-inoculated serum were used with purified HTkA to confirm antibody specificity. Purified HTkA was used as a standard to confirm HTkA specificity in total cell lysate. Anti-HTkA antibodies were confirmed with Thermococcus kodakarensis lysates and Thermococcus kodakarensis derived and purified HTkA.

Animals and other research organisms

Policy information about studies involving animals; ARRIVE guidelines recommended for reporting animal research, and Sex and Gender in Research

Laboratory animals	Components were purified from Thermococcus kodakarensis. Strain TS559 was used for all genetic constructions.
Wild animals	No wild animals were used in this study.
Reporting on sex	N/A
Field-collected samples	No field-collected samples were used in this study.
Ethics oversight	No ethics oversight was necessary or applied to the current study.

Note that full information on the approval of the study protocol must also be provided in the manuscript.

Plants

Seed stocks	N/A
Novel plant genotypes	N/A
Authentication	N/A
